# Synthesis of CF_3_-Indazoles via Rh(III)-Catalyzed C-H [4+1] Annulation of Azobenzenes with CF_3_-Imidoyl Sulfoxonium Ylides

**DOI:** 10.3390/molecules30010183

**Published:** 2025-01-05

**Authors:** Yilong Shang, Chen Li, Guiqiu Wang, Guiwei Yao, Hongliang Wu, Xun Chen, Ruirui Zhai

**Affiliations:** Engineering Research Center of Tropical Medicine Innovation and Transformation of Ministry of Education, International Joint Research Center of Human-Machine Intelligent Collaborative for Tumor Precision Diagnosis and Treatment of Hainan Province, Hainan Provincial Key Laboratory of Research and Development on Tropical Herbs, School of Pharmaceutical Sciences, Hainan Medical University, Haikou 571199, China

**Keywords:** Rh(III) catalyst, C-H activation, CF_3_-indazoles, azobenzenes, CF_3_-imidoyl sulfoxonium ylides

## Abstract

An efficient Rh(III)-catalyzed C-H activation of azobenzenes and subsequent [4+1] cascade annulation with CF_3_-imidoyl sulfoxonium ylides was developed, yielding diverse CF_3_-indazoles. This protocol featured easily available starting materials, excellent functional group tolerance and high efficiency. Moreover, the antitumor activities of selected CF_3_-indazoles against human cancer cell lines were also studied, and the results indicated that several compounds displayed considerable antiproliferative activities.

## 1. Introduction

**Figure 1:**
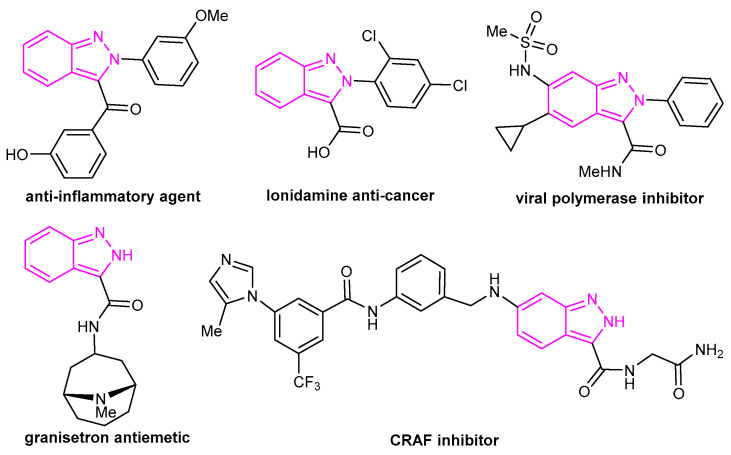
Selected bioactive molecules containing indazole motifs.

Indazoles have been identified as important skeletons found widely in natural products, drugs and agrochemicals, with a broad spectrum of biological activities, such as anti-cancer activity, viral polymerase inhibition, antiemetic and anti-inflammatory activity [1,2] (Figure 1). Thus, numerous efforts have been made for the construction of such skeletons. Directing group-assisted transition metal-catalyzed C-H bond activation/cyclization is regarded as a powerful approach for the assembly of structurally diverse molecules [3,4,5]. In this regard, azobenzenes have been used as versatile directing substrates and applied in transition metal-catalyzed C-H functionalization with various coupling partners for the synthesis of indazoles. For example, Ellman [6], Wang [7] and Kim [8] independently reported outstanding work on Co(III)-, Re(I)- or Rh(III)-catalyzed C-H bond activation and cascade annulation of azobenzenes with aldehydes to construct indazoles. Xi’s group [9] also described a Rh(III)-catalyzed C-H coupling/annulation between azobenzenes and alkenes employing Cu(OAc)_2_ as an oxidant, leading to indazoles. Moreover, Cui and co-workers [10] demonstrated an efficient synthesis of indazoles via Rh(III)-catalyzed C-H [4+1] cyclization of azobenzenes with *α*-Cl ketones. Despite these great achievements, there is great demand for the exploration of other novel and effective coupling partners to synthesize multifarious heterocycles.

**Scheme 1:**
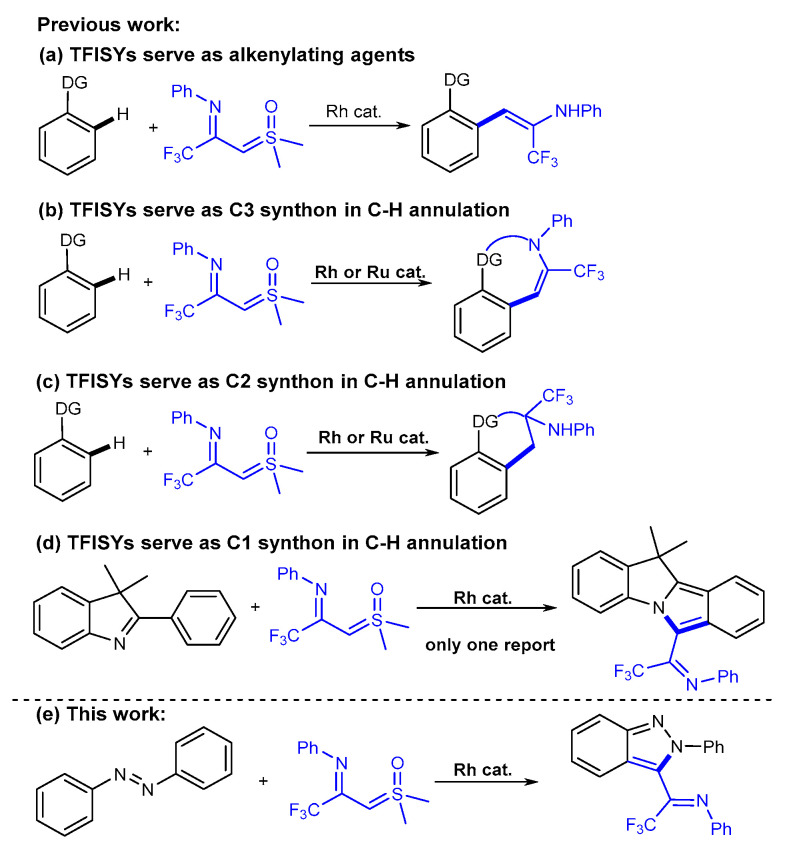
Transition metal-catalyzed C-H functionalization with TFISYs.

CF_3_-heterocycles serve as privileged frameworks that show promising application in drug development, since introducing the CF_3_ group into molecular structures could improve their pharmacological activities [11,12,13]. Recent decades have witnessed the flourishing development of the construction of CF_3_-heterocycles from various CF_3_-containing building blocks [14,15,16,17,18]. Among them, CF_3_-imidoyl sulfoxonium ylides (TFISYs), featuring considerable stability and high activity as metal carbene precursors with multifunctional synthons, have been broadly applied in transition metal-catalyzed C-H functionalization of different directing substrates for producing a series of CF_3_-heterocycles. For example, Wu’s [19] and Chen’s [20] groups employed TFISYs as alkenylating agents to achieve a Rh(III)-catalyzed C-H alkenylation of *N*-pyrimidylindoles and quinolin-8-carboxaldehydes (Scheme 1a). Moreover, Cheng [21], Wu [22,23,24] and Li [25] reported the synthesis of valuable CF_3_-heterocycles via transition metal-catalyzed C-H [n+3] annulation by using TFISYs as C3 synthons, in which cascade annulation was triggered by the attack of enamine nitrogen on an electrophilic directing group (Scheme 1b). An alternative application of TFISYs in C-H functionalization focused on the C-H [n+2] annulation induced by the addition of a nucleophilic directing group to the imine moiety of TFISYs. In this respect, Wu [26], Fan [27,28] and our group [29] independently found Rh(III)- or Ru(II)-catalyzed C-H [n+2] annulation with TFISYs assisted by different directing groups, leading to a number of CF_3_-tethered heterocycles (Scheme 1c). Moreover, Wu and co-workers [30] applied TFISYs as a C1 synthon in a Rh(III)-catalyzed C-H [4+1] annulation reaction, resulting in the formation of CF_3_-substituted isoindolo [2,1-*a*]indoles (Scheme 1d). To the best of our knowledge, there is only one successful report on the C-H [n+1] annulation by using TFISYs as C1 synthon. Motivated by the aforementioned groundbreaking works on TFISYs and our ongoing interest in the synthesis of CF_3_-heterocycles [29,31,32,33,34,35]. Herein, we report an efficient Rh(III)-catalyzed C-H [4+1] annulation reaction of azobenzenes with TFISYs, yielding diverse CF_3_-indazoles (Scheme 1e).

## 2. Results and Discussion

We commenced our investigation by using azobenzene (**1a**) and TFISY (**2a**) as the model substrates (Table 1). Encouragingly, the desired product **3a** was isolated in a 27% yield in the presence of [Cp*RhCl_2_]_2_, HOAc and AgOAc in DCE at 80 °C. Subsequently, several solvents such as MeCN, TFE, HFIP, DMF, THF and dioxane were tested (entries 2–7), among which the yield of **3a** increased to 50% with HFIP as the reaction solvent. Other commonly used metal catalysts in the C-H activation reactions, such as CoCp*(CO)I_2_, Pd(OAc)_2_, [Cp*IrCl_2_]_2_ and [Ru(p-cymene)Cl_2_]_2_, were proven to be inert or produce a low yield in this transformation (entries 8–11). The product **3a** was not obtained when the reaction was performed in the absence of a catalyst (entry 12). Subsequently, the reaction was conducted in the absence of HOAc or by using NaOAc instead of HOAc as the additive (entries 13–14), but these conditions did not improve the reaction yields. Then, we changed AgOAc to other oxidants, including Ag_2_CO_3_, Cu(OAc)_2_·H_2_O, K_2_S_2_O_8_, PhI(OAc)_2_ and O_2_ (entries 15–19), and Cu(OAc)_2_·H_2_O was found to give the best result, resulting in a 58% yield of the product **3a**. The effect of reaction temperature on this transformation was also investigated. Increasing the reaction temperature to 100 °C could lead to a slightly improved yield of 65%, while lowering the reaction temperature to 60 °C generated a decreased yield of 43% (entries 20–21). Variation of the amount of Cu(OAc)_2_·H_2_O revealed that the product **3a** could be increased to a 76% yield when 3.0 equiv of Cu(OAc)_2_·H_2_O was employed (entry 23). Reducing the loading of [Cp*RhCl_2_]_2_ catalyst from 5 mol% to 2 mol% could provide the product **3a** with a 71% yield (entry 24).

After the optimized conditions were established, we turned our attention toward examining the scope and limitation of this [4+1] annulation reaction. As shown in Table 2, TFISYs containing electron-donating groups (Me and OMe) as well as electron-withdrawing groups (F, Cl, Br, NO_2_) at the *para*-, *meta*- or *ortho*-position of benzene ring could smoothly react with **1a** to afford the corresponding annulation products **3a**–**3l** at 65–88% yields. Substrate derived from naphthalene also performed well in this catalytic system and delivered the desired product **3m** in a 76% yield. Moreover, other fluorinated (C_2_F_5_, C_3_F_7_) imidoyl sulfoxonium ylides were also allowed for this transformation to assemble the target products **3n** and **3o** at good to excellent yields. Next, the scope and generality of several azobenzenes were also studied under optimized conditions using **2b** as the coupling partner. A series of *para*-substituted azobenzenes were compatible with this reaction to produce the corresponding products **3p**–**3t** in 56–81% yields. *Meta* methyl- and chlorine-substituted azobenzenes could provide the single products **3u** (86%) and **3v** (88%) with excellent regioselectivity. Moreover, azobenzenes bearing fluorine or methyl at the *ortho*-position were well tolerated to assemble the products **3w** and **3x** in 71% and 83% yields, respectively, indicating the high steric tolerance in this transformation.

**Scheme 2:**
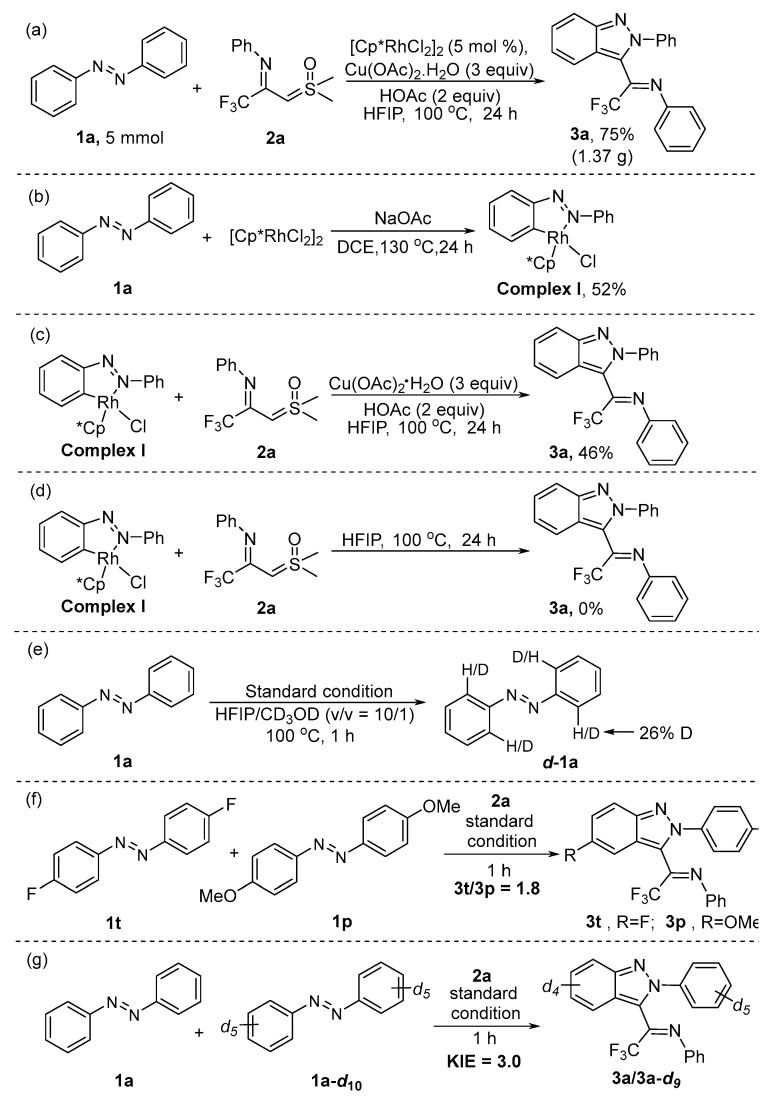
Preliminary mechanism studies.

To demonstrate the synthetic utility of this protocol, we performed an annulation reaction of **1a** with **2a** on a 5 mmol scale, and we found that the product **3a** was isolated in a 75% yield (Scheme 2a). Furthermore, we investigated the possible mechanism of this reaction by conducting several control experiments as shown in Scheme 2. Firstly, the five-membered rhodacycle complex **I** was prepared by the reaction of azobenzene **1a** with [Cp*RhCl_2_]_2_ and NaOAc in DCE at 130 °C for 24 h (Scheme 2b). Then, we used the prepared complex **I** to react with **2a** under the standard condition without [Cp*RhCl_2_]_2_, and the desired product **3a** was formed in a 46% yield (Scheme 2c), thus implying the occurrence of C-H activation in this reaction. We also performed the reaction of complex **I** with **2a** without any additive and oxidant, and we found that the desired product **3a** was not observed, highlighting the important role of Cu(OAc)_2_·H_2_O and HOAc in the success of this reaction (Scheme 2d). Moreover, a deuterium exchange of azobenzene **1a** was performed with CD_3_OD under standard condition, and 26% deuterium incorporation at the *ortho*-position of **1a** was afforded, implying that the C-H bond activation process might be reversible (Scheme 2e). Subsequently, an intermolecular competition experiment was conducted for exploring the electronic preference of this transformation. The result (**3t**/**3p** = 1.8:1) suggested that the C-H activation presumably occurs through a concerted metalation-deprotonation (CMD) process (Scheme 2f) [36]. Finally, a kinetic isotopic effect (KIE) experiment was also performed, and a value of 3.0 implied that the C-H bond cleavage was likely to be the rate-determining step of this transformation (Scheme 2g).

**Scheme 3:**
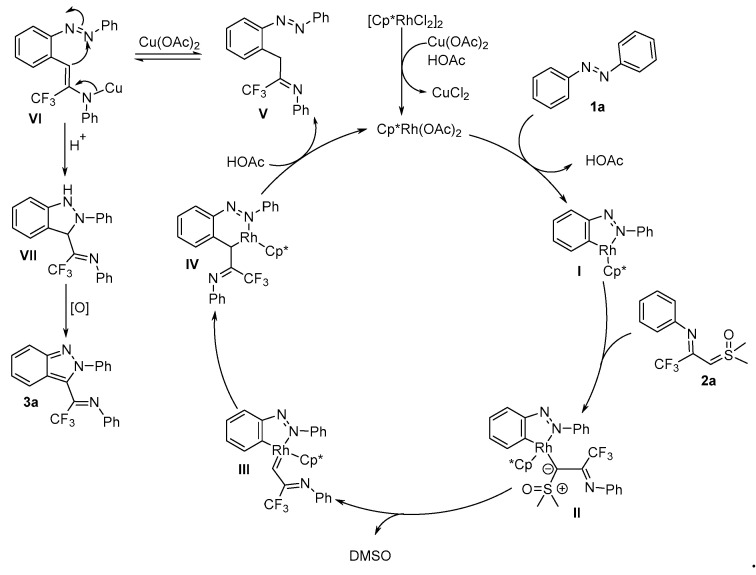
Proposed reaction mechanism.

Based on our experimental results and the previous literature on C-H functionalization with TFISYs [19,20,21,22,23,24,25,26,27,28,29,30], a plausible catalytic mechanism of this reaction is shown in Scheme 3. Initially, an active [Cp*RhCl_2_]_2_ catalyst derived from ligand exchange was coordinated with **1a** to produce the five-membered rhodacycle intermediate **I** via a C-H bond activation process. Then, the coordination of intermediate **I** with **2a** afforded the intermediate **II**, followed by the α-elimination of DMSO to deliver a Rh-carbene species intermediate **III**. Subsequently, a six-membered rhodacycle **IV** was formed through migration insertion, which underwent a protonation process to generate the intermediate **V** with regeneration of the active [Cp*Rh] catalyst. Finally, the intermediate **V** proceeded by intramolecular cyclization and oxidation under Cu(OAc)_2_·H_2_O to give the desired product **3a**.

Finally, the preliminary antitumor activities of selected CF_3_-containing indazoles against three human cancer cell lines (A549, Hela and HepG2) were studied by Cell Counting Kit-8 assay, with Gefitinib as the reference drug. As shown in Table 3, most of the compounds displayed some degree of growth inhibition against these cancer cells. For example, the inhibitory efficiency (10.2 μM) of compound **3g** against Hela cells was superior to that of Gefitinib. Compounds **3a** and **3l** exhibited equivalent cytotoxicity to that of Gefitinib against A549 cells. In addition, several compounds, such as **3g** (2.1 μM), **3m** (5.7 μM) and **3t** (4.3 μM), were found to display strong growth inhibition against HepG2 cells. These results showed that CF_3_-containing indazoles may be developed as antitumor lead compounds for drug development.

## 3. Materials and Methods

### 3.1. General Information

All reactions were carried out in flame-dried hermetic tubes with magnetic stirring. Unless otherwise noted, materials were purchased from commercial suppliers (Alfa, TCI, Sigma-Aldrich, Shanghai, China), and used without further purification. Purifications of reaction products were carried out by flash chromatography using silica gel (40–63 mm) (Qingdao Haiyang Chemical Corporation Limited, Qingdao, China) ^1^H NMR, ^13^C NMR and ^19^F NMR spectra were measured on a 400 MHz Bruker spectrometer (Bruker, Karlsruhe, Germany). The transform spectrometer was operated at 400 MHz for ^1^H NMR and 101 MHz for ^13^C NMR. ^1^H NMR chemical shifts are reported relative to TMS and were referenced via residual proton resonances of the corresponding deuterated solvent (CDCl_3_: 7.26 ppm), whereas ^13^C NMR spectra are reported relative to TMS via the carbon signals of the deuterated solvent (CDCl_3_: 77.16 ppm). All melting points were determined on XT4B melting point apparatus and uncorrected (Beijing Science Instrument Dianguang Instrument Factory, Beijing, China). High-resolution mass spectra (HRMS) were measured with an ESI source using a time-of-flight (TOF) detector (Agilent, Santa Clara, CA, USA). CF_3_-imidoyl sulfoxonium ylides were synthesized according to the previously reported procedure [37,38,39,40,41]. Azobenzenes were synthesized according to the previously reported procedure [42].

### 3.2. The General Procedure

A pressure tube was charged with azobenzenes (0.2 mmol, 1 equiv), CF_3_-imidoyl sulfoxonium ylides (0.3 mmol, 1.5 equiv), [Cp*RhCl_2_]_2_ (5 mol%), Cu(OAc)_2_·H_2_O (3 equiv), HOAc (2 equiv) and HFIP (2 mL). Then, the mixture was stirred at 100 °C under an air atmosphere for 24 h. After being cooled to ambient temperature, the mixture was filtered through a pad of Celite and concentrated under reduced pressure. The residue was purified by flash chromatography on silica gel using ethyl acetate/petroleum ether as an eluent to provide the desired products.

### 3.3. Characterization Data of Novel Compounds

(*Z*)-2,2,2-trifluoro-*N*-phenyl-1-(2-phenyl-2*H*-indazol-3-yl)ethan-1-imine (**3a**): brown solid, 55.7 mg, 76% yield, mp 83.9–85.7 °C, ^1^H NMR (400 MHz, CDCl_3_) δ: 7.81 (d, *J* = 9.7 Hz, 2H), 7.44–7.38 (m, 1H), 7.36–7.29 (m, 2H), 7.28–7.23 (m, 2H), 7.02–6.95 (m, 3H), 6.92 (t, *J* = 7.4 Hz, 2H), 6.15–6.08 (m, 2H); ^13^C NMR (101 MHz, CDCl_3_) δ: 148.93, 147.56 (q, C-F, *^2^J_C-F_* = 36.4 Hz), 145.80, 139.14, 129.27, 129.00, 128.86, 127.38, 126.85, 124.87, 124.38, 124.20, 123.22, 120.72, 119.97 (q, C-F, *^1^J_C-F_* = 279.8 Hz), 119.76 (d, C-F, *^3^J_C-F_* = 3.0 Hz), 118.68; ^19^F NMR (376 MHz, CDCl_3_) δ: −68.87; HRMS (ESI) calcd for C_21_H_15_F_3_N_3_ (M + H)^+^: 366.1213, found 366.1211.

(*Z*)-2,2,2-trifluoro-1-(2-phenyl-2*H*-indazol-3-yl)-*N*-(p-tolyl)ethan-1-imine (**3b**): yellow solid, 66.7 mg, 88% yield, mp 82.2–84.7 °C, ^1^H NMR (400 MHz, CDCl_3_) δ: 7.76–7.70 (m, 2H), 7.37–7.31 (m, 1H), 7.29–7.15 (m, 4H), 6.98–6.91 (m, 2H), 6.67 (d, *J* = 7.8 Hz, 2H), 6.02–5.96 (m, 2H), 2.13 (s, 3H); ^13^C NMR (101 MHz, CDCl_3_) δ: 148.97, 146.63 (q, C-F, *^2^J_C-F_* = 36.4 Hz), 143.32, 139.18, 137.16, 129.44, 129.16, 128.98, 127.35, 125.55 (q, C-F, *^1^J_C-F_* = 279.8 Hz), 124.74, 124.39, 124.06, 123.58, 121.05, 119.82 (d, C-F, *^3^J_C-F_* = 2.0 Hz), 118.64, 21.08; ^19^F NMR (376 MHz, CDCl_3_) δ: −68.68; HRMS (ESI) calcd for C_22_H_17_F_3_N_3_ (M + H)^+^: 380.1310, found 380.1369.

(*Z*)-2,2,2-trifluoro-*N*-(4-methoxyphenyl)-1-(2-phenyl-2*H*-indazol-3-yl)ethan-1-imine (**3c**): brown oil, 51.4 mg, 65% yield, ^1^H NMR (400 MHz, CDCl_3_) δ: 7.75 (dt, *J* = 8.8, 1.0 Hz, 1H), 7.71 (dd, *J* = 8.6, 1.0 Hz, 1H), 7.34 (ddd, *J* = 8.7, 6.6, 1.1 Hz, 1H), 7.28–7.17 (m, 4H), 7.01–6.97 (m, 2H), 6.44–6.40 (m, 2H), 6.13–6.09 (m, 2H), 3.62 (s, 3H); ^13^C NMR (101 MHz, CDCl_3_) δ: 158.72, 148.76, 144.74 (q, C-F, *^2^J_C-F_* = 36.4 Hz), 138.87, 138.50, 128.84, 128.69, 127.08, 124.39, 124.01, 123.50, 123.48, 123.12, 119.83 (q, C-F, *^1^J_C-F_* = 279.8 Hz), 119.53 (d, C-F, *^3^J_C-F_* = 2.0 Hz), 118.31, 113.83, 55.21; ^19^F NMR (376 MHz, CDCl_3_) δ: −68.71; HRMS (ESI) calcd for C_22_H_17_F_3_N_3_O (M + H)^+^: 396.1319, found 396.1320.

(*Z*)-*N*-(4-chlorophenyl)-2,2,2-trifluoro-1-(2-phenyl-2*H*-indazol-3-yl)ethan-1-imine (**3d**): brown solid, 52.7 mg, 66% yield, mp 94.7–96.3 °C, ^1^H NMR (400 MHz, CDCl_3_) δ: 7.82–7.66 (m, 2H), 7.39–7.33 (m, 1H), 7.32–7.18 (m, 4H), 7.01–6.94 (m, 2H), 6.86–6.80 (m, 2H), 6.00–5.94 (m, 2H); ^13^C NMR (101 MHz, CDCl_3_) δ: 149.01, 148.29 (q, C-F, *^2^J_C-F_* = 36.4 Hz), 144.31, 139.07, 132.62, 129.42, 129.32, 129.16, 129.01, 127.54, 126.07, 125.17, 124.31, 124.19, 122.70, 122.08, 119.88 (q, C-F, *^1^J_C-F_* = 280.8 Hz), 119.65 (d, C-F, *^3^J_C-F_* = 3.0 Hz), 118.81; ^19^F NMR (376 MHz, CDCl_3_) δ: −68.91; HRMS (ESI) calcd for C_21_H_14_Cl F_3_N_3_ (M + H)^+^: 400.0823, found 400.0823.

(*Z*)-*N*-(4-bromophenyl)-2,2,2-trifluoro-1-(2-phenyl-2*H*-indazol-3-yl)ethan-1-imine (**3e**): yellow solid, 69.1 mg, 78% yield, mp 119.7–121.7 °C, ^1^H NMR (400 MHz, CDCl_3_) δ: 7.78–7.70 (m, 2H), 7.38–7.33 (m, 1H), 7.32–7.17 (m, 4H), 7.00–6.94 (m, 4H), 5.93–5.87 (m, 2H); ^13^C NMR (101 MHz, CDCl_3_) δ: 148.99, 148.30 (q, C-F, *^2^J_C-F_* = 37.4 Hz), 144.77, 139.05, 131.96, 129.41, 129.14, 127.52, 125.17, 124.30, 124.19, 122.64, 122.30, 120.50, 119.88 (q, C-F, *^1^J_C-F_* = 279.8 Hz), 119.62 (d, C-F, *^3^J_C-F_* = 3 Hz), 118.80; ^19^F NMR (376 MHz, CDCl_3_) δ: −68.81; HRMS (ESI) calcd for C_21_H_14_BrF_3_N_3_ (M + H)^+^: 444.0318, found 444.0315.

(*Z*)-2,2,2-trifluoro-*N*-(4-fluorophenyl)-1-(2-phenyl-2*H*-indazol-3-yl)ethan-1-imine (**3f**): yellow solid, 59.0 mg, 72% yield, mp 147.4–151.3 °C, ^1^H NMR (400 MHz, CDCl_3_) δ 7.77–7.65 (m, 4H), 7.38–7.30 (m, 2H), 7.30–7.14 (m, 3H), 6.95 (d, *J* = 7.8 Hz, 2H), 6.07 (d, *J* = 9.0 Hz, 2H); ^13^C NMR (101 MHz, CDCl_3_) δ 151.21, 150.57 (q, C-F, *^2^J_C-F_* = 37.4 Hz), 148.95, 145.44, 138.91, 129.69, 129.40, 127.69, 125.69, 124.42, 124.18, 121.77, 120.96, 120.50, 119.57 (q, C-F, ^1^*J_C-F_* = 280.8 Hz), 119.35 (d, C-F, *^3^J_C-F_* = 3.0 Hz), 118.95, 118.18; ^19^F NMR (376 MHz, CDCl_3_) δ −68.70; HRMS (ESI) calcd for C_21_H_14_F_3_N_4_O_2_ (M + H)^+^: 411.1064, found 411.1070.

(*Z*)-2,2,2-trifluoro-*N*-(4-fluorophenyl)-1-(2-phenyl-2*H*-indazol-3-yl)ethan-1-imine (**3g**): yellow solid, 62.0 mg, 81% yield, mp 79.9–81.6 °C, ^1^H NMR (400 MHz, CDCl_3_) δ: 7.00–6.89 (m, 2H), 6.57–6.51 (m, 1H), 6.49–6.36 (m, 4H), 6.20–6.14 (m, 2H), 5.80–5.70 (m, 2H), 5.27–5.17 (m, 2H); ^13^C NMR (101 MHz, CDCl_3_) δ: 162.91, 160.44, 149.35, 147.93 (q, C-F, *^2^J_C-F_* = 37.4 Hz), 142.23, 139.40, 129.66, 129.41, 127.81, 125.37, 124.56, 124.41, 123.16, 123.07, 120.27 (q, C-F, *^1^J_C-F_* = 278.8 Hz), 119.98, 119.08, 116.13, 116.01; ^19^F NMR (376 MHz, CDCl_3_) δ: −68.85, −114.35; HRMS (ESI) calcd for C_21_H_14_F_4_N_3_ (M + H)^+^: 384.1119, found 384.1118.

(*Z*)-2,2,2-trifluoro-1-(2-phenyl-2*H*-indazol-3-yl)-*N*-(o-tolyl)ethan-1-imine (**3h**): brown solid, 66.0 mg, 87% yield, mp 91.2–94.6 °C, ^1^H NMR (400 MHz, CDCl_3_) δ: 8.12–7.66 (m, 2H), 7.37–7.23 (m, 3H), 7.23–7.14 (m, 2H), 6.89–6.77 (m, 4H), 6.61 (t, *J* = 6.8 Hz, 1H), 5.72 (d, *J* = 8.0 Hz, 1H), 1.52 (s, 3H); ^13^C NMR (101 MHz, CDCl_3_) δ: 148.89, 146.17 (q, C-F, *^2^J_C-F_* = 37.4 Hz), 144.16, 139.36, 133.59, 130.51, 129.30, 129.21, 127.38, 127.34, 125.95, 124.77, 124.69, 124.27, 123.65, 119.96 (q, C-F, *^1^J_C-F_* = 279.8 Hz), 119.78 (d, C-F, *^3^J_C-F_* = 3 Hz), 118.65, 117.63, 17.12; ^19^F NMR (376 MHz, CDCl_3_) δ: -68.79; HRMS (ESI) calcd for C_22_H_17_F_3_N_3_ (M + H)^+^: 380.1370, found 380.1367.

(*Z*)-2,2,2-trifluoro-*N*-(2-methoxyphenyl)-1-(2-phenyl-2*H*-indazol-3-yl)ethan-1-imine (**3i**): yellow solid, 61.6 mg, 78% yield, mp 118.1–120.2 °C, ^1^H NMR (400 MHz, CDCl_3_) δ: 7.80–7.67 (m, 2H), 7.34–7.26 (m, 2H), 7.26–7.14 (m, 3H), 7.01 (dd, *J* = 7.6, 2.1 Hz, 2H), 6.90–6.84 (m, 1H), 6.49–6.36 (m, 2H), 5.93 (dd, *J* = 8.2, 1.7 Hz, 1H), 3.07 (s, 3H); ^13^C NMR (101 MHz, CDCl_3_) δ: 149.64, 148.84, 148.07 (q, C-F, *^2^J_C-F_* = 36.4 Hz), 139.31, 134.87, 129.23, 128.73, 127.85, 127.16, 126.02, 124.47, 124.24, 123.45, 121.20, 120.37, 120.10 (d, C-F, *^3^J_C-F_* = 3 Hz), 119.88 (q, C-F, *^1^J_C-F_* = 279.8 Hz), 118.39, 110.78, 55.75; ^19^F NMR (376 MHz, CDCl_3_) δ: −68.19; HRMS (ESI) calcd for C_22_H_17_F_3_N_3_O (M + H)^+^: 396.1319, found 396.1317.

(*Z*)-*N*-(2-bromophenyl)-2,2,2-trifluoro-1-(2-phenyl-2*H*-indazol-3-yl)ethan-1-imine (**3j**): yellow solid, 62.0 mg, 70% yield, mp 101.8–102.4 °C, ^1^H NMR (400 MHz, CDCl_3_) δ: 7.75 (t, *J* = 7.9 Hz, 2H), 7.35 (ddd, *J* = 10.7, 7.1, 2.0 Hz, 2H), 7.30–7.16 (m, 4H), 6.96–6.91 (m, 2H), 6.78 (dtd, *J* = 18.0, 7.5, 1.7 Hz, 2H), 5.81 (d, *J* = 7.7 Hz, 1H).; ^13^C NMR (101 MHz, CDCl_3_) δ: 148.88, 148.40 (q, C-F, *^2^J_C-F_* = 37.4 Hz), 144.14, 139.08, 133.22, 129.66, 129.59, 128.14, 127.50, 127.42, 125.05, 124.22, 124.16, 122.63, 119.71 (q, C-F, *^1^J_C-F_* = 280.8 Hz), 119.69 (d, C-F, *^3^J_C-F_* = 3.0 Hz), 119.34, 119.28, 118.69; ^19^F NMR (376 MHz, CDCl_3_) δ: −68.55; HRMS (ESI) calcd for C_21_H_14_BrF_3_N_3_ (M + H)^+^: 444.0318, found 444.0315.

(*Z*)-2,2,2-trifluoro-1-(2-phenyl-2*H*-indazol-3-yl)-*N*-(m-tolyl)ethan-1-imine (**3k**): yellow solid, 66.7 mg, 88% yield, mp 73.9–75.8 °C, ^1^H NMR (400 MHz, CDCl_3_) δ: 6.97–6.90 (m, 2H), 6.53 (ddd, *J* = 8.9, 6.7, 1.0 Hz, 1H), 6.49–6.35 (m, 4H), 6.15–6.09 (m, 2H), 5.94–5.88 (m, 2H), 1.14 (s, 3H); ^13^C NMR (101 MHz, CDCl_3_) δ: 148.90, 147.49 (q, C-F, *^2^J_C-F_* = 37.4 Hz), 145.86, 139.13, 138.84, 129.18, 128.81, 128.53, 127.54, 127.34, 124.80, 124.45, 124.22, 123.44, 122.22, 119.98 (q, C-F, *^1^J_C-F_* = 279.8 Hz), 119.79, 118.64, 116.51, 21.22; ^19^F NMR (376 MHz, CDCl_3_) δ: −68.87; HRMS (ESI) calcd for C_22_H_17_F_3_N_3_ (M + H)^+^: 380.1370, found 380.1367.

(*Z*)-*N*-(3-chlorophenyl)-2,2,2-trifluoro-1-(2-phenyl-2*H*-indazol-3-yl)ethan-1-imine (**3l**): yellow solid, 69.4 mg, 87% yield, mp 74.0–77.4 °C, ^1^H NMR (400 MHz, CDCl_3_) δ: 7.74 (d, *J* = 9.4 Hz, 2H), 7.39–7.30 (m, 2H), 7.30–7.13 (m, 3H), 7.00–6.84 (m, 3H), 6.75 (t, *J* = 8.0 Hz, 1H), 6.02 (t, *J* = 2.0 Hz, 1H), 5.81 (d, *J* = 7.4 Hz, 1H); ^13^C NMR (101 MHz, CDCl_3_) δ: 149.08 (q, C-F, *^2^J_C-F_* = 36.4 Hz), 147.03, 138.87, 134.76, 129.71, 129.58, 129.46, 129.23, 127.44, 126.55, 125.16, 124.25, 122.64, 121.74, 119.76 (q, C-F, *^1^J_C-F_* =280.8 Hz), 119.56, 118.76, 117.12; ^19^F NMR (376 MHz, CDCl_3_) δ: −68.86; HRMS (ESI) calcd for C_21_H_14_ClF_3_N_3_ (M + H)^+^: 400.0823, found 400.0822.

(*Z*)-2,2,2-trifluoro-*N*-(naphthalen-2-yl)-1-(2-phenyl-2*H*-indazol-3-yl)ethan-1-imine (**3m**): brown solid, 63.1 mg, 76% yield, mp 84.4–86.3 °C, ^1^H NMR (400 MHz, CDCl_3_) δ: 7.83–7.76 (m, 1H), 7.73 (d, *J* = 8.8 Hz, 1H), 7.59 (d, *J* = 11.0 Hz, 1H), 7.38–7.23 (m, 6H), 7.20–7.14 (m, 1H), 7.01–6.94 (m, 2H), 6.80–6.72 (m, 2H), 6.56 (d, *J* = 2.1 Hz, 1H), 6.13 (dd, *J* = 8.7, 2.1 Hz, 1H); ^13^C NMR (101 MHz, CDCl_3_) δ: 149.00, 147.80 (q, C-F, *^2^J_C-F_* = 37.4 Hz), 143.34, 139.18, 133.39, 131.90, 129.17, 129.01, 128.70, 128.37, 127.69, 127.46, 126.63, 126.47, 125.01, 124.30, 124.12, 123.49, 120.21, 120.10 (q, C-F, *^1^J_C-F_* = 279.8 Hz), 119.84 (d, C-F, *^3^J_C-F_* = 2.0 Hz), 118.91, 118.74; ^19^F NMR (376 MHz, CDCl_3_) δ: −68.65; HRMS (ESI) calcd for C_25_H_17_F_3_N_3_ (M + H)^+^: 416.1370, found 416.1368.

(*Z*)-2,2,3,3,3-pentafluoro-*N*-phenyl-1-(2-phenyl-2*H*-indazol-3-yl)propan-1-imine (**3n**): yellow solid, 67.2 mg, 81% yield, mp 86.5–82.9 °C, ^1^H NMR (400 MHz, CDCl_3_) δ: 7.74 (d, *J* = 8.7 Hz, 1H), 7.70 (d, *J* = 9.5 Hz, 1H), 7.33 (ddd, *J* = 8.7, 6.7, 1.1 Hz, 1H), 7.30–7.14 (m, 4H), 6.98–6.85 (m, 5H), 6.16–6.05 (m, 2H); ^13^C NMR (101 MHz, CDCl_3_) δ: 148.90, 148.36 (q, C-F, *J* = 36.4 Hz), 145.82, 139.25, 129.22, 129.04, 128.89, 127.39, 127.13, 124.86, 124.39, 124.03, 123.44, 120.92, 119.02 (q, C-F, *J* = 279.8 Hz), 119.84, 119.79, 118.64; ^19^F NMR (376 MHz, CDCl_3_) δ; −79.83, −111.54, −111.66; HRMS (ESI) calcd for C_22_H_15_F_5_N_3_ (M + H)^+^: 416.1181, found 416.1177.

(*Z*)-2,2,3,3,4,4,4-heptafluoro-*N*-phenyl-1-(2-phenyl-2*H*-indazol-3-yl)butan-1-imine (**3o**): yellow solid, 77.6 mg, 81% yield, mp 65.1–66.7 °C, ^1^H NMR (400 MHz, CDCl_3_) δ: 7.74 (d, *J* = 8.8 Hz, 1H), 7.61 (d, *J* = 8.6 Hz, 1H), 7.37–7.27 (m, 2H), 7.27–7.14 (m, 3H), 7.08–7.00 (m, 2H), 6.99–6.89 (m, 3H), 6.30–6.15 (m, 2H); ^13^C (101 MHz, CDCl_3_) δ: 148.59, 145.54, 139.12, 128.96, 128.79, 128.65, 127.06, 126.97, 124.53, 124.09, 123.47, 123.35, 120.69, 119.39, 118.82, 118.33, 109.06, 112.60; ^19^F NMR (376 MHz, CDCl_3_) δ: −79.28 (t, *J* = 10.1 Hz), −108.37, −108.84, −123.23, −123.38; HRMS (ESI) calcd for C_23_H_15_F_7_N_3_ (M + H)^+^: 446.1149, found 446.1151.

(*Z*)-2,2,2-trifluoro-1-(5-methoxy-2-(4-methoxyphenyl)-2*H*-indazol-3-yl)-*N*-(p-tolyl)ethan-1-imine (**3p**): yellow solid, 44.0 mg, 50% yield, mp 157.9–159.5 °C, ^1^H NMR (400 MHz, CDCl_3_) δ: 7.61 (d, *J* = 9.3 Hz, 1H), 7.02 (dd, *J* = 9.4, 2.3 Hz, 1H), 6.87–6.81 (m, 3H), 6.76–6.66 (m, 4H), 6.10 (d, *J* = 8.3 Hz, 2H), 3.79 (d, *J* = 23.4 Hz, 6H), 2.16 (s, 3H); ^13^C NMR (101 MHz, CDCl_3_) δ: 159.81, 157.09, 146.86 (q, C-F, *^2^J_C-F_* = 37.4 Hz), 145.49, 143.47, 137.06, 132.48, 129.36, 125.40, 124.23, 122.48, 122.22, 121.15, 120.13 (q, C-F, *^1^J_C-F_* = 279.8 Hz), 119.80, 114.18, 95.70, 55.68, 55.50, 21.06; ^19^F NMR (376 MHz, CDCl_3_) δ: −68.81; HRMS (ESI) calcd for C_24_H_21_F_3_N_3_O_2_(M + H)^+^: 440.1581, found 440.1579.

(*Z*)-2,2,2-trifluoro-1-(5-methyl-2-(p-tolyl)-2*H*-indazol-3-yl)-*N*-(p-tolyl)ethan-1-imine (**3q**): yellow solid, 66.0 mg, 81% yield, mp 112.7–114.0 °C, ^1^H NMR (400 MHz, CDCl_3_) δ: 6.47 (d, *J* = 9.7 Hz, 1H), 6.29 (s, 1H), 6.06–5.98 (m, 1H), 5.83 (d, *J* = 8.2 Hz, 2H), 5.65 (d, *J* = 8.4 Hz, 2H), 5.53 (d, *J* = 8.2 Hz, 2H), 4.88 (d, *J* = 8.4 Hz, 2H), 1.27 (s, 3H), 1.14 (s, 3H), 1.00 (s, 3H); ^13^C NMR (101 MHz, CDCl_3_) δ: 147.82, 146.98 (q, C-F, *^2^J_C-F_* = 37.4 Hz), 143.44, 138.95, 137.05, 136.86, 134.35, 130.18, 129.61, 129.33, 124.38, 124.11, 122.58, 121.15, 120.06 (q, C-F, *^1^J_C-F_* = 279.8 Hz), 118.18, 117.79 (d, C-F, *^3^J_C-F_* = 2.0 Hz), 22.22, 21.24, 21.10; ^19^F NMR (376 MHz, CDCl_3_) δ: −68.80; HRMS (ESI) calcd for C_24_H_21_F_3_N_3_(M + H)^+^: 408.1683, found 408.1686.

(*Z*)-1-(5-chloro-2-(4-chlorophenyl)-2*H*-indazol-3-yl)-2,2,2-trifluoro-*N*-(p-tolyl)ethan-1-imine (**3r**): yellow solid, 50.1 mg, 56% yield, mp 125.2–127.9 °C, ^1^H NMR (400 MHz, CDCl_3_) δ: 6.94–6.81 (m, 2H), 6.48 (dd, *J* = 9.2, 1.9 Hz, 1H), 6.39–6.33 (m, 2H), 6.09–6.00 (m, 2H), 5.92 (d, *J* = 8.2 Hz, 2H), 5.20 (d, *J* = 6.4 Hz, 2H), 1.36 (s, 3H); ^13^C NMR (101 MHz, CDCl_3_) δ: 147.40, 145.43 (q, C-F, *^2^J_C-F_* = 37.4 Hz), 143.00, 137.71, 137.34, 135.38, 130.96, 129.58, 129.36, 129.23, 125.46, 124.38, 123.34, 121.09, 120.17, 119.90 (q, C-F, *^1^J_C-F_* = 279.8 Hz), 118.60 (d, C-F, *^3^J_C-F_* = 3.0 Hz), 21.12; ^19^F NMR (376 MHz, CDCl_3_) δ: -68.84; HRMS (ESI) calcd for C_22_H_15_Cl_2_F_3_N_3_(M + H)^+^: 448.0590, found 448.0586.

(*Z*)-1-(5-bromo-2-(4-bromophenyl)-2*H*-indazol-3-yl)-2,2,2-trifluoro-*N*-(p-tolyl)ethan-1-imine (**3s**): yellow solid, 70.9 mg, 66% yield, mp 121.0–123.2 °C, ^1^H NMR (400 MHz, CDCl_3_) δ: 7.91 (s, 1H), 7.61 (dd, *J* = 9.3, 3.7 Hz, 1H), 7.42 (dd, *J* = 9.2, 2.9 Hz, 1H), 7.36–7.16 (m, 2H), 6.76 (ddd, *J* = 22.0, 8.5, 3.3 Hz, 4H), 6.01 (dd, *J* = 8.5, 3.5 Hz, 2H), 2.17 (d, *J* = 3.7 Hz, 3H); ^13^C NMR (101 MHz, CDCl_3_) δ: 147.49, 145.46 (q, C-F, *^2^J_C-F_* = 37.4 Hz), 142.99, 137.79, 137.74, 132.36, 131.53, 129.60, 125.72, 125.10, 123.42, 123.12, 122.00 (d, C-F, *^3^J_C-F_* = 2.0 Hz), 121.09, 120.33, 119.86 (q, C-F, *^1^J_C-F_* = 279.8 Hz), 118.95, 21.14; ^19^F NMR (376 MHz, CDCl_3_) δ: −68.82; HRMS (ESI) calcd for C_22_H_15_Br_2_F_3_N_3_(M + H)^+^: 537.9559, found 537.9558.

(*Z*)-2,2,2-trifluoro-1-(5-fluoro-2-(4-fluorophenyl)-2*H*-indazol-3-yl)-*N*-(p-tolyl)ethan-1-imine (**3t**): yellow solid, 64.8 mg, 78% yield, mp 125.4–127.9 °C, ^1^H NMR (400 MHz, CDCl_3_) δ: 7.71 (dd, *J* = 9.4, 4.7 Hz, 1H), 7.31 (d, *J* = 8.4 Hz, 1H), 7.18–7.12 (m, 1H), 6.88 (d, *J* = 6.4 Hz, 4H), 6.73 (d, *J* = 8.2 Hz, 2H), 6.02 (d, *J* = 8.4 Hz, 2H), 2.16 (s, 3H); ^13^C NMR (101 MHz, CDCl_3_) δ: 164.03, 161.44, 158.91, 146.35, 145.73 (q, C-F, *^2^J_C-F_* = 36.4 Hz), 143.12, 137.58, 135.16, 129.57, 126.11, 123.95, 123.60, 121.11, 120.87, 119.99 (q, C-F, *^1^J_C-F_* = 279.8 Hz), 119.52, 119.23, 116.18, 102.77, 21.10; ^19^F NMR (376 MHz, CDCl_3_) δ: −68.96, −111.28, −114.77; HRMS (ESI) calcd for C_22_H_15_F_5_N_3_(M + H)^+^: 416.1181, found 416.1181.

(*Z*)-2,2,2-trifluoro-1-(6-methyl-2-(m-tolyl)-2*H*-indazol-3-yl)-*N*-(p-tolyl)ethan-1-imine (**3u**): brown solid, 70.0 mg, 86% yield, mp 85.1–87.3 °C, ^1^H NMR (400 MHz, CDCl_3_) δ: 7.70 (d, *J* = 8.7 Hz, 1H), 7.55 (s, 1H), 7.20–7.10 (m, 3H), 6.88 (d, *J* = 5.8 Hz, 1H), 6.76 (d, *J* = 6.8 Hz, 2H), 6.63 (s, 1H), 6.05 (d, *J* = 6.5 Hz, 2H), 2.51 (s, 3H), 2.23 (s, 6H); ^13^C NMR (101 MHz, CDCl_3_) δ: 149.53, 147.24 (q, C-F, *^2^J_C-F_* = 36.4 Hz), 143.44, 139.51, 139.06, 137.36, 136.86, 129.49, 129.35, 128.72, 127.65, 125.26, 123.36, 122.47, 121.20, 121.06, 120.04 (q, C-F, *^1^J_C-F_* = 279.8 Hz), 119.31, 116.82, 22.26, 21.26, 21.06; ^19^F NMR (376 MHz, CDCl_3_) δ: −68.92; HRMS (ESI) calcd for C_24_H_21_F_3_N_3_ (M + H)^+^: 408.1683, found 408.1684.

(*Z*)-1-(6-chloro-2-(3-chlorophenyl)-2*H*-indazol-3-yl)-2,2,2-trifluoro-*N*-(p-tolyl)ethan-1-imine (**3v**): brown solid, 78.7 mg, 88% yield, mp 143.3–147.7 °C, ^1^H NMR (400 MHz, CDCl_3_) δ: 7.73–7.67 (m, 2H), 7.25 (dd, *J* = 8.3, 1.7 Hz, 1H), 7.21–7.15 (m, 2H), 6.93 (d, *J* = 8.0 Hz, 1H), 6.74 (d, *J* = 8.4 Hz, 2H), 6.67 (t, *J* = 1.9 Hz, 1H), 5.95 (d, *J* = 8.3 Hz, 2H), 2.19 (s, 3H); ^13^C NMR (101 MHz, CDCl_3_) δ: 149.27, 145.59 (q, C-F, *^2^J_C-F_* = 36.4 Hz), 142.95, 139.66, 137.93, 135.36, 133.75, 129.95, 129.70, 129.30, 126.69, 125.24, 124.32, 122.46, 122.01, 121.19, 120.86, 119.84 (q, C-F, *^1^J_C-F_* = 279.8 Hz), 117.59, 20.31; ^19^F NMR (376 MHz, CDCl_3_) δ: −68.88; HRMS (ESI) calcd for C_22_H_15_Cl_2_F_3_N_3_(M + H)^+^: 448.0590, found 448.0593.

(*Z*)-2,2,2-trifluoro-1-(7-fluoro-2-(2-fluorophenyl)-2*H*-indazol-3-yl)-*N*-(p-tolyl)ethan-1-imine (**3w**): brown solid, 58.9 mg, 71% yield, mp 100.4–103.2 °C, ^1^H NMR (400 MHz, CDCl_3_) δ: 7.51 (d, *J* = 7.0 Hz, 1H), 7.26 (dd, *J* = 13.3, 7.6 Hz, 1H), 7.19–7.11 (m, 1H), 7.07–6.96 (m, 2H), 6.89 (t, *J* = 7.8 Hz, 1H), 6.74 (dd, *J* = 14.5, 7.0 Hz, 3H), 6.07 (dd, *J* = 8.4, 1.9 Hz, 2H), 2.15 (s, 3H); ^13^C NMR (101 MHz, CDCl_3_) δ: 156.69, 154.32 (d, *J* = 29.3 Hz), 151.88, 146.07 (q, C-F, *^2^J_C-F_* = 36.4 Hz), 143.30, 140.68 (d, *J* = 17.2 Hz), 137.43, 131.10 (d, *J* = 8.1 Hz), 129.69, 127.79, 127.22 (d, *J* = 11.1 Hz), 126.41, 126.05, 124.96 (dd, *J* = 18.2 Hz), 120.81, 119.60 (q, C-F, *^2^J_C-F_* = 279.8 Hz), 116.62 (d, *J* = 20.2 Hz), 115.76, 110.48 (d, *J* = 16.2 Hz), 21.08; ^19^F NMR (376 MHz, CDCl_3_) δ: −68.04, −123.04, −127.09; HRMS (ESI) calcd for C_22_H_14_F_5_N_3_Na (M + Na)^+^: 438.1001, found 438.1005.

(*Z*)-2,2,2-trifluoro-1-(7-methyl-2-(o-tolyl)-2*H*-indazol-3-yl)-*N*-(p-tolyl)ethan-1-imine (**3x**): brown solid, 67.6 mg, 83% yield, mp 116.6–118.0 °C, ^1^H NMR (400 MHz, CDCl_3_) δ: 7.54 (d, *J* = 9.2 Hz, 1H), 7.24–7.15 (m, 1H), 7.15–7.03 (m, 4H), 6.77 (d, *J* = 8.1 Hz, 3H), 6.17 (d, *J* = 8.2 Hz, 2H), 2.56 (s, 3H), 2.15 (s, 3H), 1.53 (s, 3H); ^13^C NMR (101 MHz, CDCl_3_) δ: 148.97, 147.52 (q, C-F, *^2^J_C-F_* = 36.4 Hz), 143.90, 138.25, 137.04, 135.56, 131.67, 131.07, 129.69, 129.56, 128.95, 126.30, 126.24, 126.15, 124.86, 123.72, 121.20, 120.79, 118.86 (q, C-F, *^1^J_C-F_* = 279.8 Hz), 117.08, 21.05, 17.35, 17.16; ^19^F NMR (376 MHz, CDCl_3_) δ: −68.61; HRMS (ESI) calcd for C_24_H_21_F_3_N_3_ (M + H)^+^: 408.1683, found 408.1684.

## 4. Conclusions

In conclusion, we successfully developed a novel and efficient Rh(III)-catalyzed C-H [4+1] annulation of azobenzenes with CF_3_-imidoyl sulfoxonium ylides via C-H bond activation, synthesizing various CF_3_-containing indazoles. This transformation was characterized by easily available starting materials, good tolerance of functional groups and excellent regioselectivity. Moreover, in vitro cytotoxicities of selected products were investigated against three kinds of human cancer cells (A549, Hela and HepG2), and several products exhibited excellent antitumor activities.

## Figures and Tables

**Table 1:**
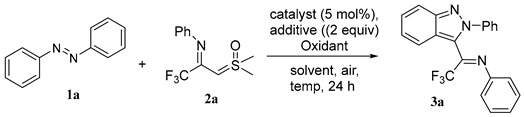
Optimization of reaction conditions ^[a]^.

Entry	Catalyst	Additive	Oxidant	Solvent	Yield ^[b]^
1	[Cp*RhCl_2_]_2_	HOAc	AgOAc	DCE	27
2	[Cp*RhCl_2_]_2_	HOAc	AgOAc	MeCN	21
3	[Cp*RhCl_2_]_2_	HOAc	AgOAc	TFE	45
4	[Cp*RhCl_2_]_2_	HOAc	AgOAc	HFIP	50
5	[Cp*RhCl_2_]_2_	HOAc	AgOAc	DMF	0
6	[Cp*RhCl_2_]_2_	HOAc	AgOAc	THF	0
7	[Cp*RhCl_2_]_2_	HOAc	AgOAc	Dioxane	0
8	Cp*Co(CO)I_2_	HOAc	AgOAc	HFIP	0
9	Pd(OAc)_2_	HOAc	AgOAc	HFIP	0
10	[Cp*IrCl_2_]_2_	HOAc	AgOAc	HFIP	0
11	[Ru(*p*-cymene)Cl_2_]_2_	HOAc	AgOAc	HFIP	12
12	none	HOAc	AgOAc	HFIP	0
13	[Cp*RhCl_2_]_2_	none	AgOAc	HFIP	42
14	[Cp*RhCl_2_]_2_	NaOAc	AgOAc	HFIP	36
15	[Cp*RhCl_2_]_2_	HOAc	Ag_2_CO_3_	HFIP	54
16	[Cp*RhCl_2_]_2_	HOAc	Cu(OAc)_2_·H_2_O	HFIP	58
17	[Cp*RhCl_2_]_2_	HOAc	K_2_S_2_O_8_	HFIP	0
18	[Cp*RhCl_2_]_2_	HOAc	PhI(OAc)_2_	HFIP	0
19	[Cp*RhCl_2_]_2_	HOAc	O_2_	HFIP	0
20	[Cp*RhCl_2_]_2_	HOAc	Cu(OAc)_2_·H_2_O	HFIP	43 ^[c]^
21	[Cp*RhCl_2_]_2_	HOAc	Cu(OAc)_2_·H_2_O	HFIP	65 ^[d]^
22	[Cp*RhCl_2_]_2_	HOAc	Cu(OAc)_2_·H_2_O	HFIP	57 ^[e]^
23	[Cp*RhCl_2_]_2_	HOAc	Cu(OAc)_2_·H_2_O	HFIP	76 ^[f]^
24	[Cp*RhCl_2_]_2_	HOAc	Cu(OAc)_2_·H_2_O	HFIP	71 ^[g]^

^[a]^ Reaction conditions: **1a** (0.2 mmol), **2a** (0.3 mmol), catalyst (5 mol%), oxidant (2 equiv), additive (2 equiv), solvent (2 mL), air, 80 °C, 24 h; ^[b]^ isolated yield; ^[c]^ at 60 °C; ^[d]^ at 100 °C; ^[e]^ 1 equiv of oxidant; ^[f]^ 3 equiv of oxidant; ^[g]^ 2 mol% [Cp*RhCl_2_]_2_.

**Table 2:** Substrate scope ^[a,b]^.

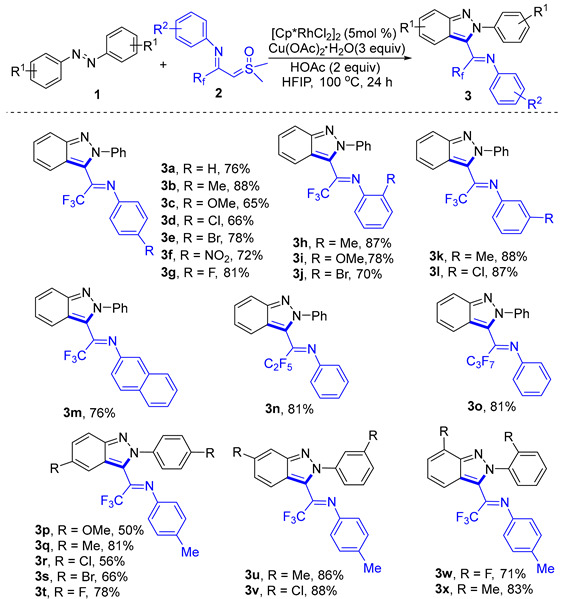
^[a]^ Reaction conditions: 1 (0.2 mmol), 2 (0.3 mmol), [Cp*RhCl_2_]_2_ (5 mol%), HOAc (2 equiv), Cu(OAc)_2_·H_2_O (3 equiv), HFIP (2 mL), air, 100 °C, 24 h; ^[b]^ isolated yield.

**Table 3:** Evaluation of antitumor activities of selected compounds.

Compound	IC_50_ (μM)		
	A549	Hela	HepG2
**3a**	12.8	26.8	51.2
**3b**	29.4	57.3	37.3
**3g**	25.4	10.2	2.1
**3i**	34.0	42.6	81.2
**3l**	12.3	79.8	11.6
**3m**	20.3	87.6	5.7
**3o**	19.9	40.3	53.0
**3p**	39.4	107.0	100.2
**3s**	41.2	42.6	89.5
**3t**	63.8	30.0	4.3
**3v**	51.2	100.5	25.7
**3y**	55.7	164.0	22.4
**Gefitinib**	12.8	12.8	2.3

## Data Availability

The data underlying this study are available in the published article and its Supporting Information.

## Supplementary Materials

The following supporting information can be downloaded at https://www.mdpi.com/article/10.3390/molecules30010183/s1: detailed experimental procedures, characterization data of all compounds, cytotoxic activity evaluation, NMR spectra and HRMS spectra for all compounds.
