# Supplementary material for: Synthesis of CF3-Indazoles via Rh(III)-Catalyzed C-H [4+1] Annulation of Azobenzenes with CF3-Imidoyl Sulfoxonium Ylides
Source: Molecules. 2025 Jan 5;30(1):183. doi: 10.3390/molecules30010183 (PMC11720978; doi:10.3390/molecules30010183)
Supplement: Supplementary file 1 [file molecules-30-00183-s001.zip › molecules-3397154-supplementary.pdf]

Supporting Information

Synthesis of CF_3-Indazoles via Rh(III)-Catalyzed C-H [4+1] Annulation of Azobenzenes with CF_3-Imidoyl Sulfoxonium Ylides

Yilong Shang,† Chen Li,† Guiqiu Wang, Guiwei Yao, Hongliang Wu, Xun Chen* and Ruirui Zhai*

Engineering Research Center of Tropical Medicine Innovation and Transformation of Ministry of Education, International Joint Research Center of Human-machine Intelligent Collaborative for Tumor Precision Diagnosis and Treatment of Hainan Province, Hainan Provincial Key Laboratory of Research and Development on Tropical Herbs, School of Pharmaceutical Sciences, Hainan Medical University, Haikou 571199, China.

*Corresponding author: chenxun@muhn.edu.cn; zhairuirui@muhn.edu.cn

†These authors contributed equally to this paper

Table of Contents

1.Gram-Scale reaction for the synthesis of **3a** 2

2. Mechanism studies 2

3. CytotoxicActivity Evaluation 5

4. References 5

5. ^{1}H NMR, ^{13}C NMR and ^{19}F NMR spectrum for all isolated compounds 6

6. HRMS spectrum for all unknown compounds 43

1.Gram-Scale reaction for the synthesis of 3a

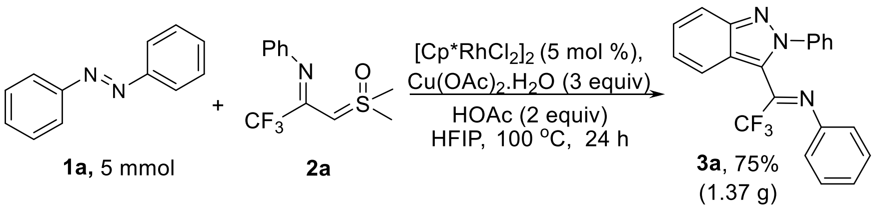

A Schlenk flask was charged with azobenzene **1a** (0.91 g, 5 mmol), CF_3-imidoyl sulfoxonium ylide **2a** (1.97g, 7.5 mmol), $[Cp^*RhCl_2]_2$ (5 mol%), $Cu(OAc)_2{\cdot}H_2O$ (3 equiv), HOAc (2 equiv) and HFIP (15 mL).Then the mixture was stirred at 100 ^oC under air atmosphere for 24 h. After being cooled to ambient temperature, the mixture was filtered through a pad of Celite and concentrated under reduced pressure. The residue was purified by flash chromatography on silica gel using ethyl acetate/petroleum ether as eluent to afford the desired product **3a** (1.37 g, 75%).

2. Mechanism studies

2.1 The preparation of complex I[1]

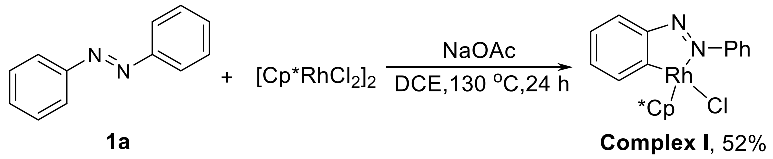

A pressure tube was charged with azobenzene **1a** (36.4mg, 0.2 mmol), $[Cp^*RhCl_2]_2$ (60 mg, 0.1 mmol), NaOAc (32.8 mg, 0.4 mmol) and DCE (1 mL). Then the mixturewas stirred at 130 ^oC under air atmosphere for 24 h. After being cooled to ambient temperature, the mixture were filtered through a pad of Celite and concentrated under reduced pressure. The residue was purified by flash chromatography on silica gel using ethyl acetate/petroleum ether as eluent to afford the five-membered cyclorhodium complex **I** (47.1 mg, 52%).

Complex **I**: ^1H NMR (400 MHz, $CDCl_3$) δ: 8.18 (dd, *J* = 7.7, 1.5 Hz, 1H), 8.04 – 7.98 (m, 2H), 7.88 (dd, *J* = 7.6, 1.3 Hz, 1H), 7.53 – 7.46 (m, 3H), 7.29 (td, *J* = 7.4, 1.5 Hz, 1H), 7.22 (td, *J* = 7.4, 1.3 Hz, 1H), 1.49 (s, 15H); ^{13}C NMR (101 MHz, $CDCl_3$) δ: 193.50, 178.61, 165.53, 158.94, 151.43, 143.78, 137.11, 137.00, 130.58, 123.55, 122.87, 122.02, 119.14, 96.08, 96.02, 9.26; HRMS (ESI) calcd for $C_{22}H_{25}ClN_2Rh$ $(M + H)^+$: 455.0756, found 455.0758.

2.2 The transformation of complex I

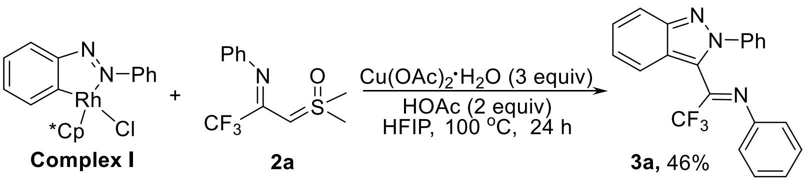

A pressure tube was charged with complex species **I** (90.6 mg, 0.2 mmol), CF_3-imidoyl sulfoxonium ylide **2a** (78.9 mg, 0.3 mmol), $Cu(OAc)_2{\cdot}H_2O$ (119.8 mg, 0.6 mmol), HOAc (24 mg, 0.2 mmol) and HFIP (2mL).Then the mixture was stirred at 100 ^oC under air atmosphere for 24 h. After being cooled to ambient temperature, the mixture were filtered through a pad of Celite and concentrated under reduced pressure. The residue was purified by flash chromatography on silica gel using ethyl acetate/petroleum ether as eluent to afford the desired product **3a** in 46% yield.

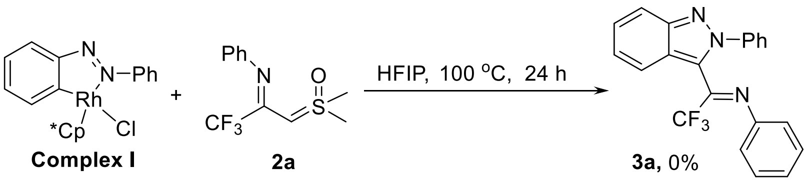

A pressure tube was charged with complex species **I** (90.6 mg, 0.2 mmol), CF_3-imidoyl sulfoxonium ylide **2a** (78.9 mg, 0.3 mmol), and HFIP (2mL).Then the mixture was stirred at 100 °C under air atmosphere for 24 h. The desired product **3a** was not observed.

2.3 H/D exchange experiments

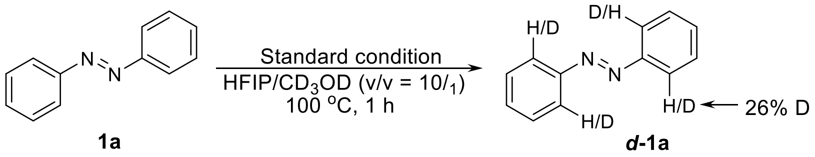

A pressure tube was charged with azobenzene **1a** (0.2 mmol), CD_3OD (0.2mL), $[Cp^*RhCl_2]_2$ (5 mol%), $Cu(OAc)_2 \cdot H_2O$ (3 equiv), HOAc (2 equiv) and HFIP (1 mL). Then the mixture was stirred at 100 °C under air atmosphere for 1h. After being cooled to ambient temperature, the mixture were filtered through a pad of Celite and concentrated under reduced pressure. The residue was purified by flash chromatography on silica gel using ethyl acetate/petroleum ether as eluent to afford the product ***d*-1a**. The deuterium incorporation of ***d*-1a** was determined to be 26% by the ^1H NMR analysis (**Figure S1**).

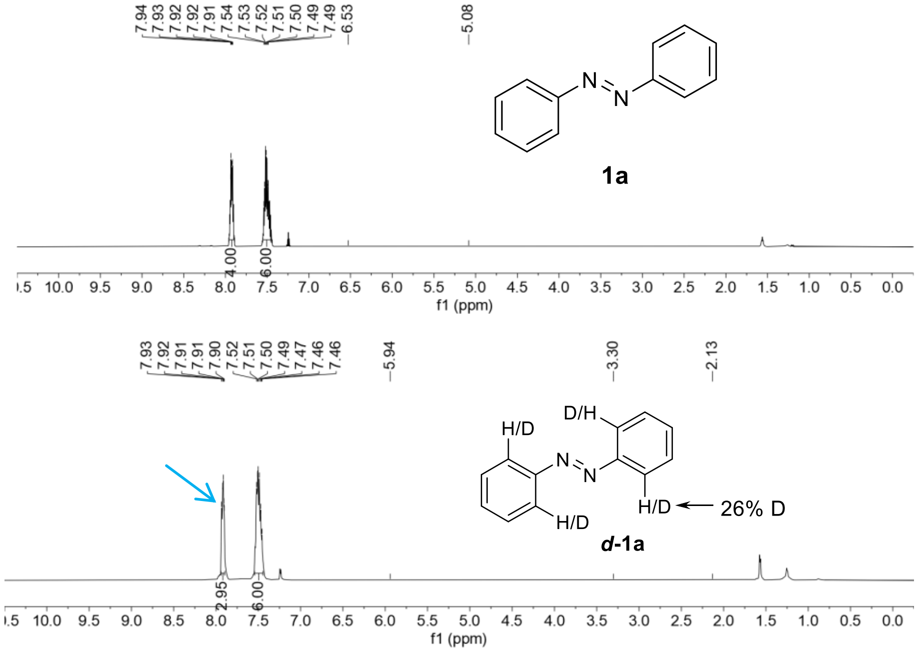

Figure S1. The ^1H NMR of **1a** and ***d*-1a**

2.4 Competition experiments

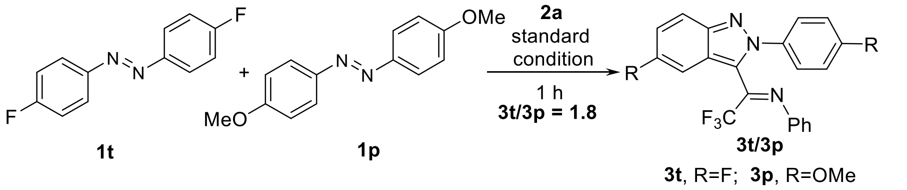

A pressure tube was charged with 4-fluoro disubstituted azobenzene **1t** (0.10 mmol),

4-methoxy disubstituted azobenzene1p **1p** (0.10 mmol), CF_3-imidoyl sulfoxonium ylide **2a** (0.3 mmol), $[Cp^*RhCl_2]_2$ (5 mol%), $Cu(OAc)_2·H_2O$ (3 equiv), HOAc (2 equiv) and HFIP (2 mL) . Then the mixture was stirred at 100 °C under air atmosphere for 1h. After being cooled to ambient temperature, the mixture were filtered through a pad of Celite and concentrated under reduced pressure. The residue was purified by flash chromatography to afford the mixture of **3t/3p**, and the ratio of **3t/3p** (**3t/3p** = 1.8:1) was determined by the ^{1}H NMR analysis (**Figure S2**).

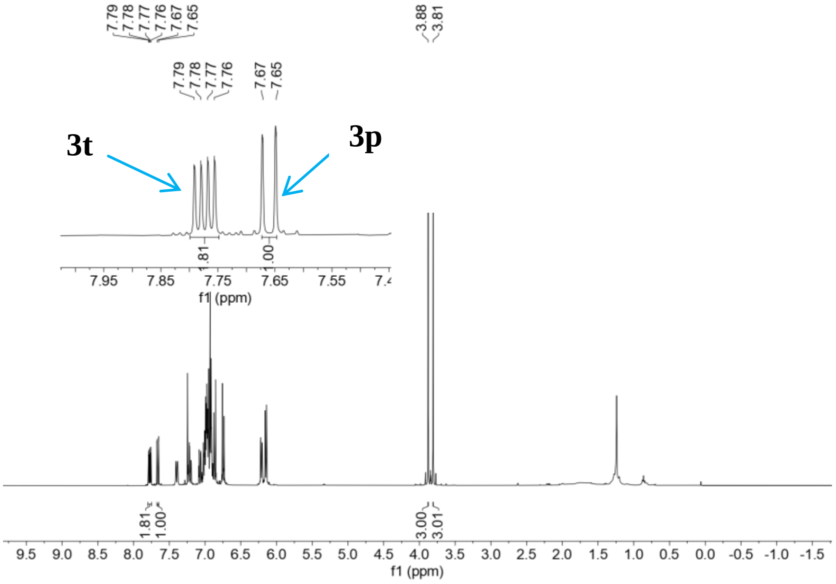

Figure S2. The ^{1}H NMR of **3t/3p** mixture

2.5 Kinetic isotope effect study

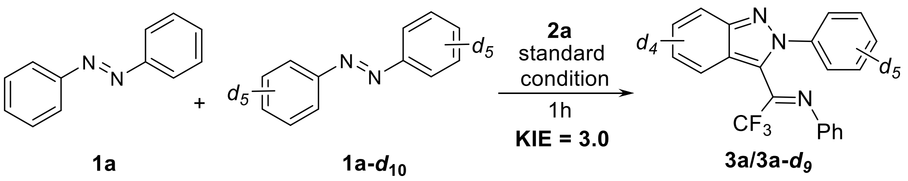

A pressure tube was charged with **1a** (0.10 mmol), **1a-*d*$_{10}$** (0.10 mmol), CF_3-imidoyl sulfoxonium ylide **2a** (0.3 mmol), $[Cp^*RhCl_2]_2$ (5 mol%), $Cu(OAc)_2·H_2O$ (3 equiv), HOAc (2 equiv) and HFIP (2 mL). Then the mixture was stirred at 100 °C under air atmosphere for 1 h. After being cooled to ambient temperature, the solvent was removed under reduced pressure. The residue was purified by flash chromatography on silica gel using ethyl acetate/petroleum ether as eluent to afford a mixture of product **3a** and **3a-d$_9$**, which was further analyzed by ^{1}H NMR spectrum. The ^{1}H NMR data indicated a kinetic isotope effect (KIE) of 3.0 (**Figure S3**).

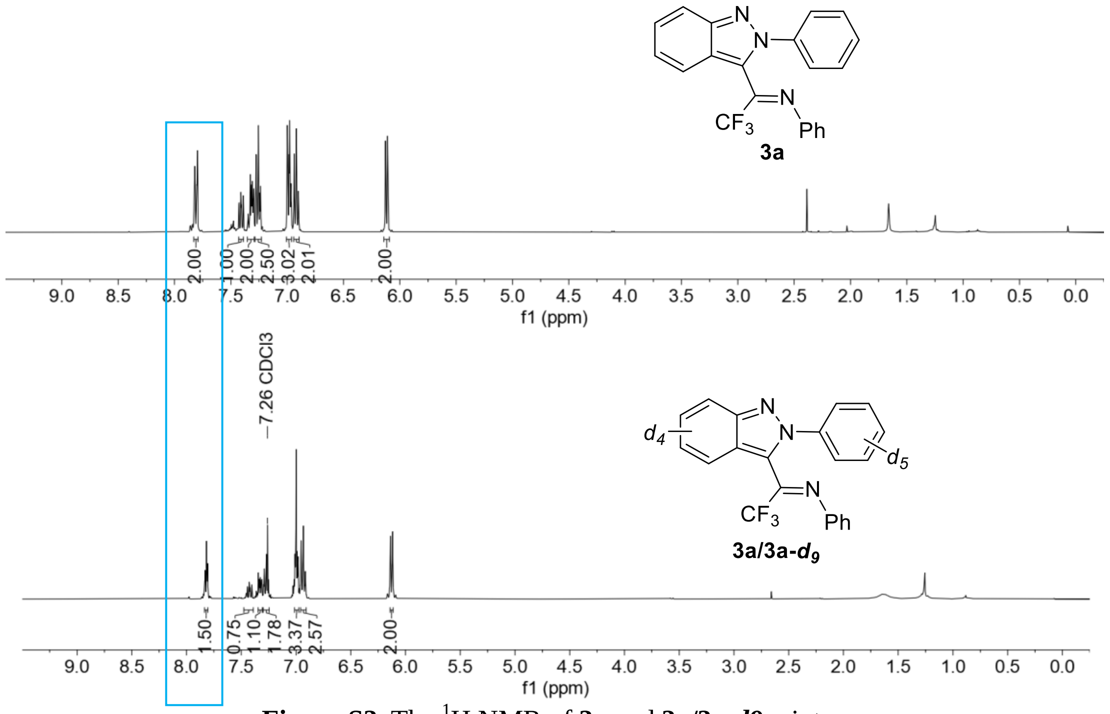

Figure S3. The ^{1}H NMR of **3a** and **3a/3a-*d9*** mixture

3. CytotoxicActivity Evaluation

The in vitro cytotoxicity of selected compound against A549 cells (human lung adenocarcinoma cell line), HepG2 cells (human liver carcinoma cell line) and Hela cells (human cervical carcinoma cell line) were evaluated by Cell Counting Kit-8 assay (CCK-8) with Gefitinib as the positive control. Dilute A549, HepG2 or Hela cell suspensions in growth medium to desired density and 100 μL were taken to 96-well plate. These cell lines were cultured with standard methods in DMEM medium containing 20% FBS (Gibco, CA, USA). The tested compounds were dissolved in DMSO and then diluted with 20% FBS DMEM medium to prepare the tested compounds with different concentration gradients. The exponentially growing cells were seeded on 96-well plates (5×103 cells per well) and incubated for 24h. Subsequently, cells were treated with designated concentrations of compounds for 24 h. Then, 100 μL mixed solution (V_{CCK-8}: V_{DMEM} = 1:10) were added to each well, the plates were incubated at 37°C for an additional 3-5 h, and then, absorbance was read at 450 nm. Cell inhibition was calculated using the following formula: Cell inhibition rate = (control OD value - test OD)/(control OD value - blank OD value) ×100%. The half maximal inhibitory concentration (IC_{50}) values were determined using GraphPad Prism 5 software (Graph Pad, La Jolla, CA, USA).

4. References

[1] Cai S.; Lin S.; Yi X.; Xi C. *J. Org. Chem.* **2017**, *82*, 512-520.

5. ^{1}H NMR, ^{13}C NMR and ^{19}F NMR spectrum for all isolated compounds

(1) ^{1}H NMR, ^{13}C NMR and ^{19}F NMR spectrum of **3a** (using $CDCl_3$ as solvent)

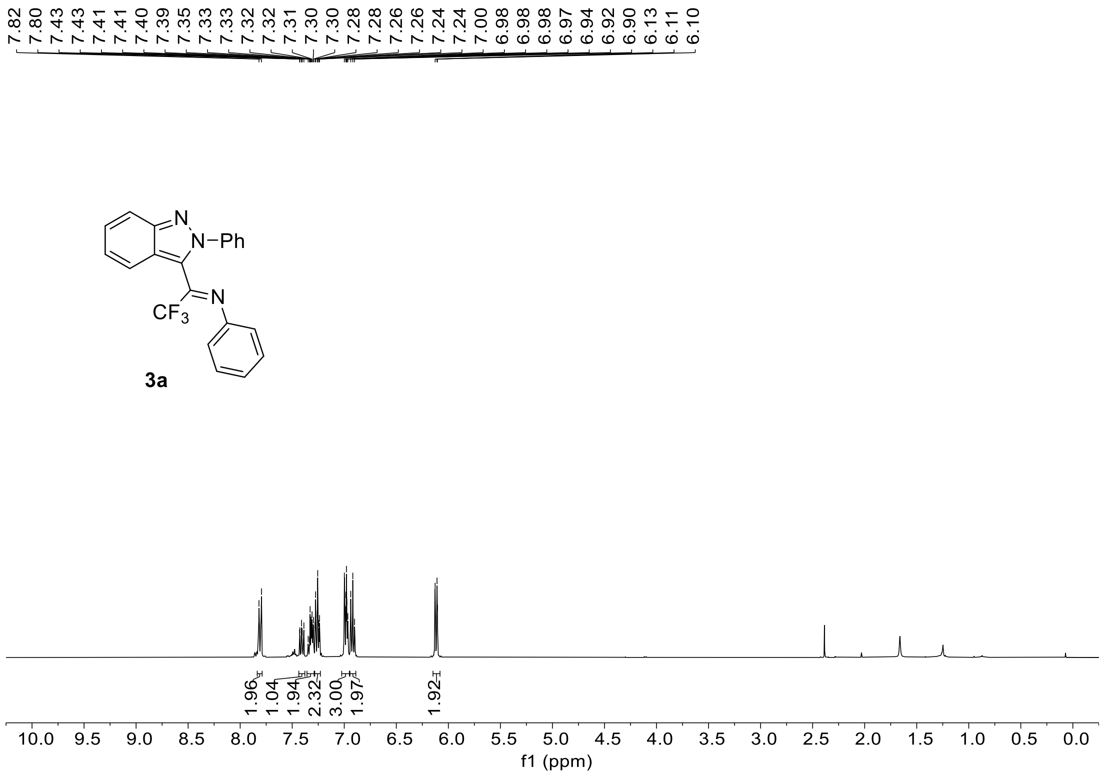

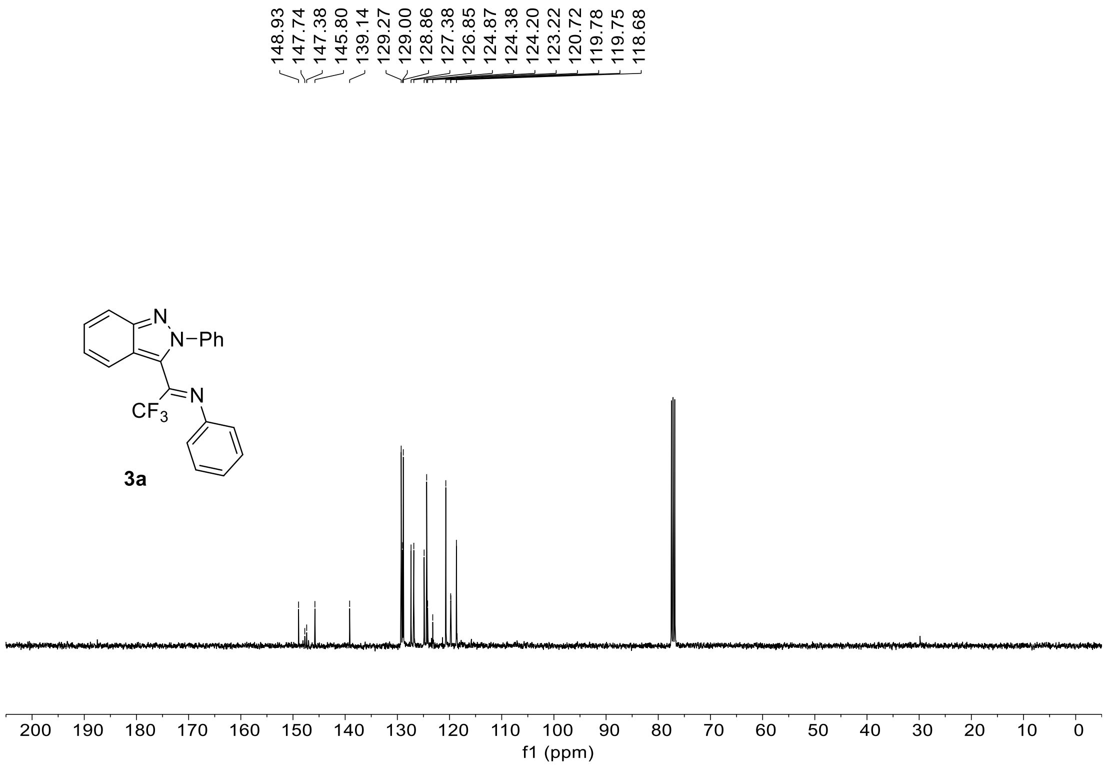

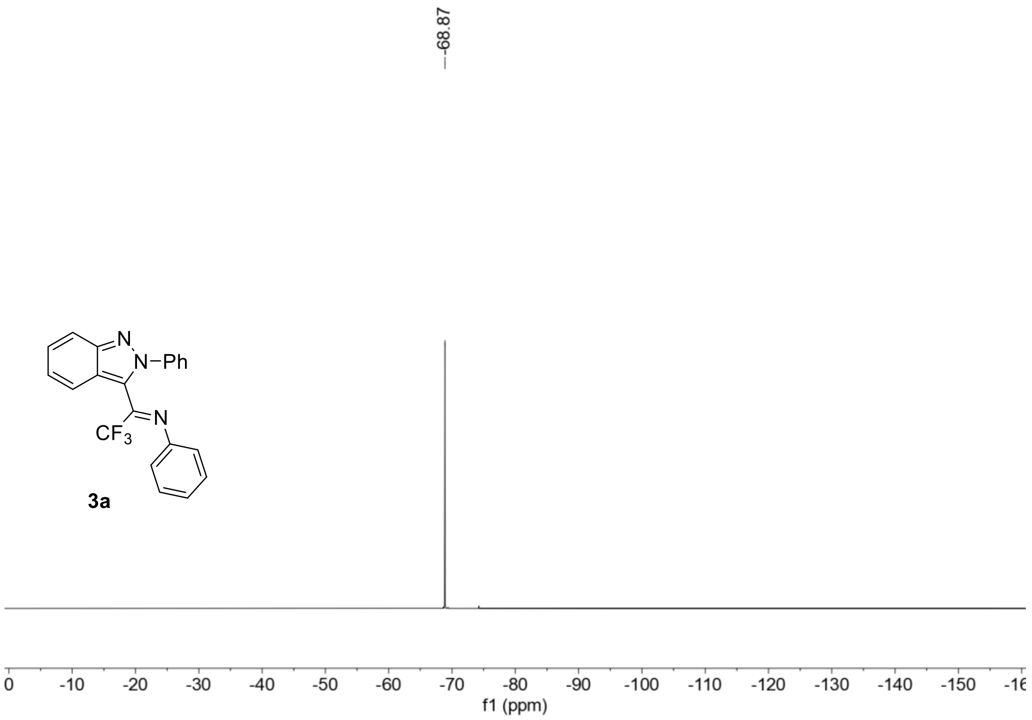

(2) ^{1}H NMR, ^{13}C NMR and ^{19}F NMR spectrum of **3b** (using $CDCl_3$ as solvent)

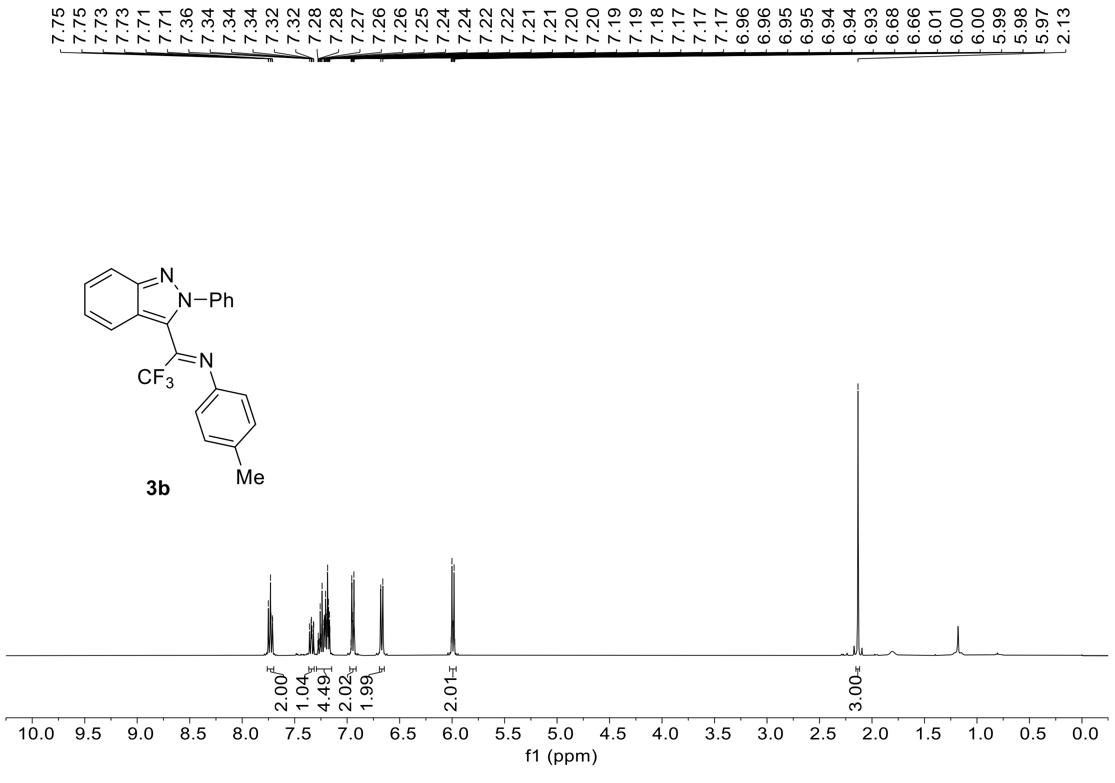

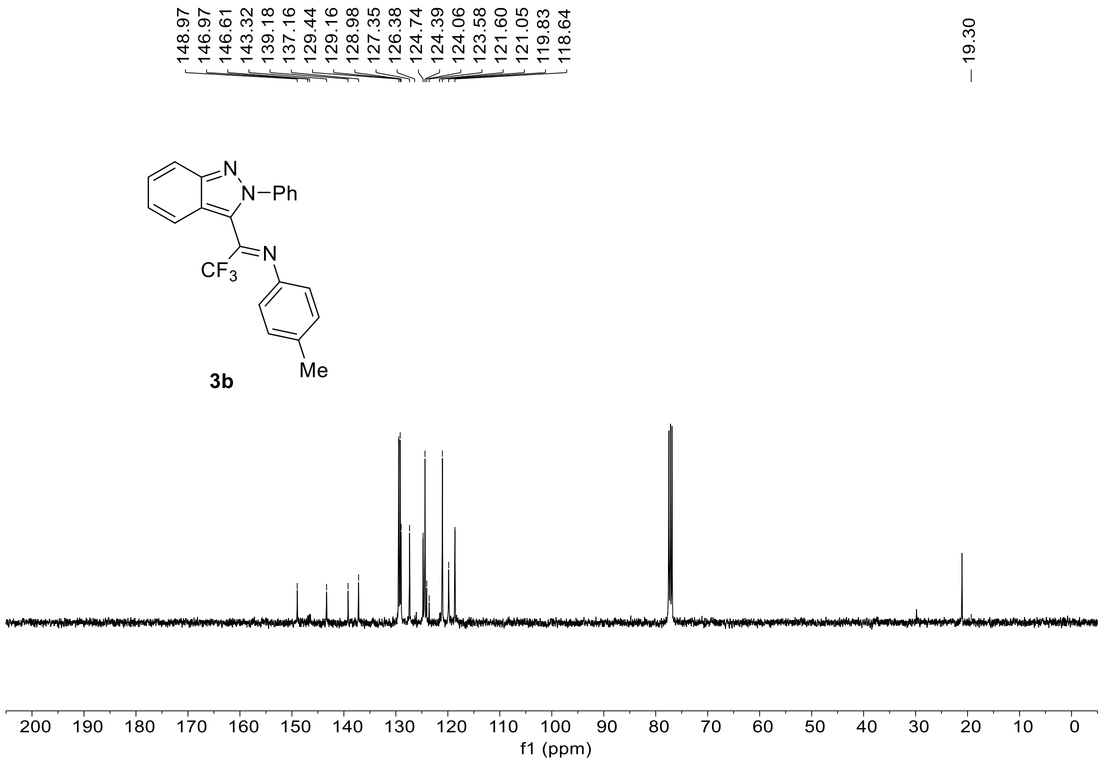
148.97
146.97
146.61
143.32
139.18
137.16
129.44
129.16
128.98
127.35
126.38
124.74
124.39
124.06
123.58
121.60
121.05
119.83
118.64
19.30
N
N−Ph
CF3
N
Me
3b
200 190 180 170 160 150 140 130 120 110 100 90 80 70 60 50 40 30 20 10 0
f1 (ppm)

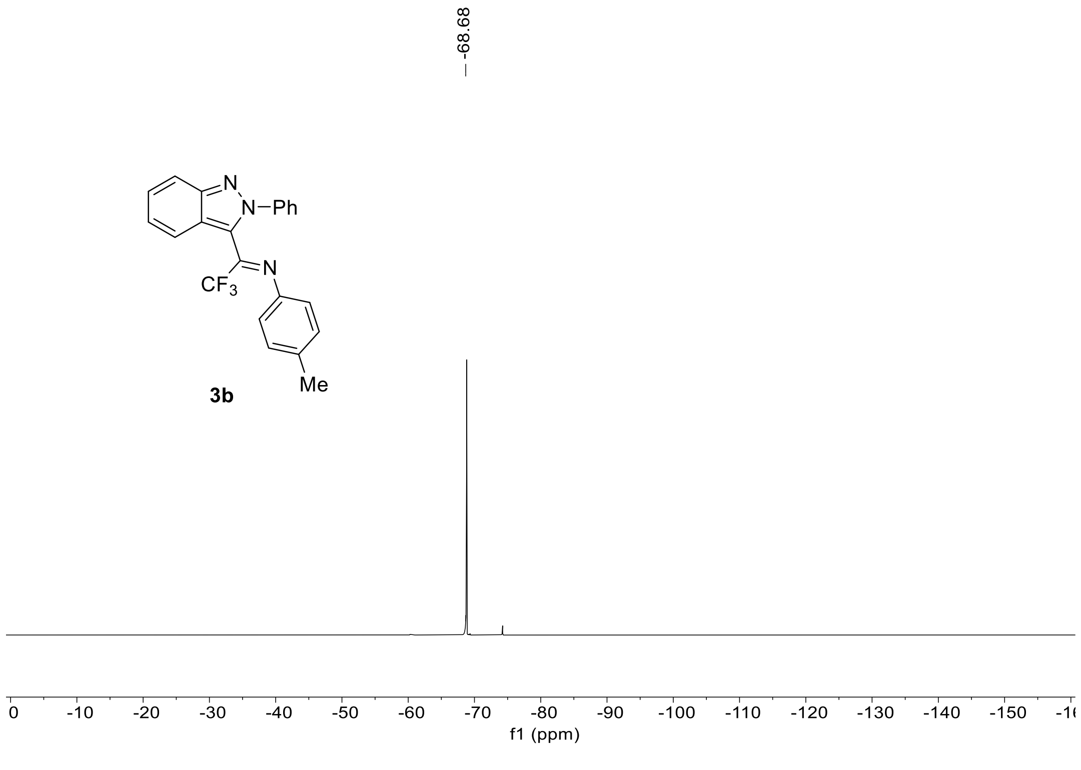
-68.68
N
N−Ph
CF3
N
Me
3b
0 -10 -20 -30 -40 -50 -60 -70 -80 -90 -100 -110 -120 -130 -140 -150
f1 (ppm)

(3) ^{1}H NMR, ^{13}C NMR and ^{19}F NMR spectrum of **3c** (using $CDCl_3$ as solvent)

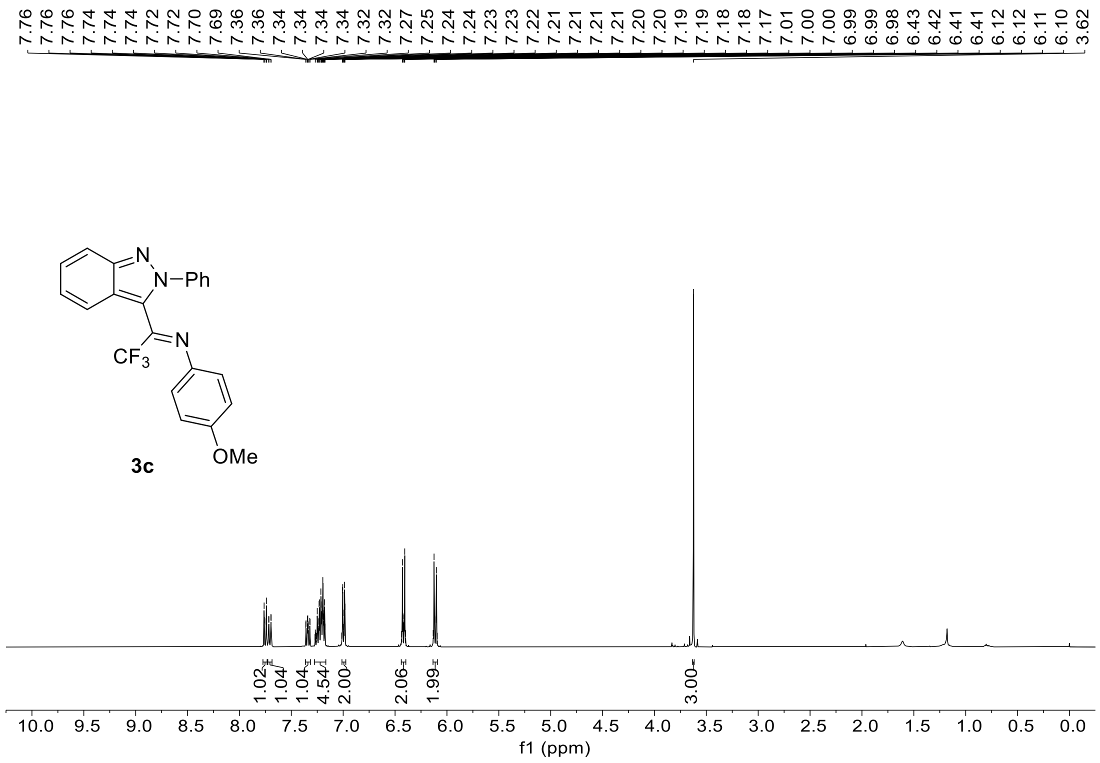

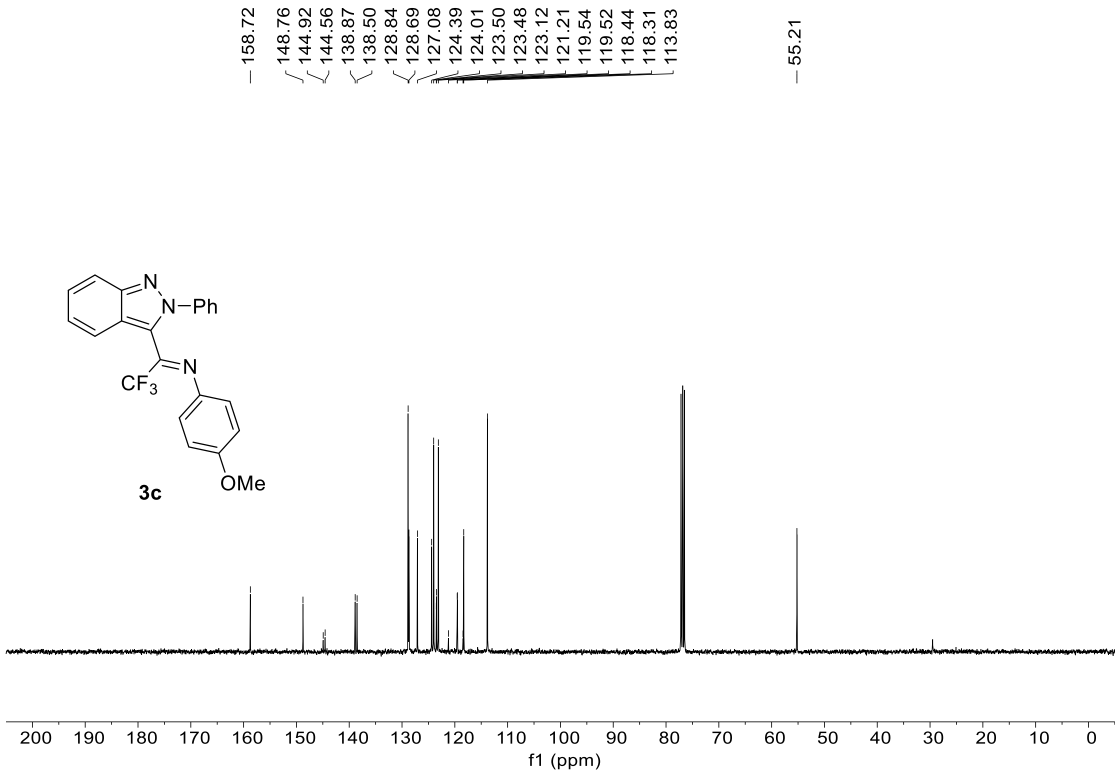

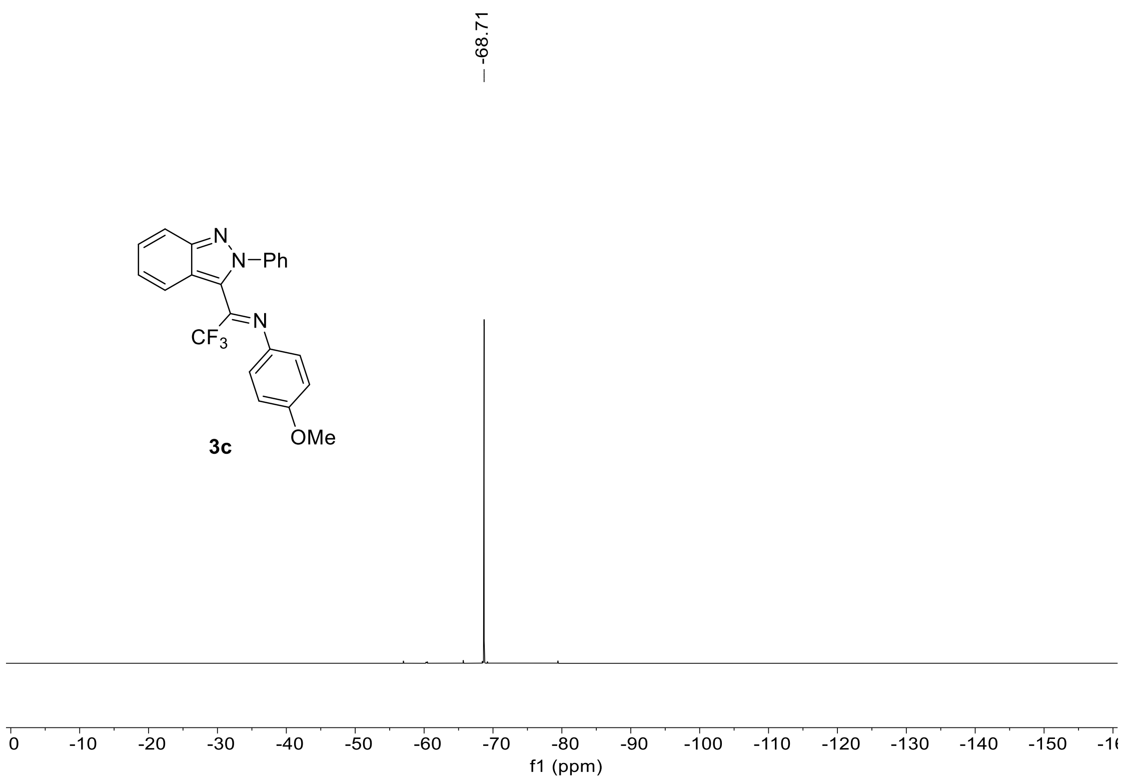

(4) ^{1}H NMR, ^{13}C NMR and ^{19}F NMR spectrum of **3d** (using $CDCl_3$ as solvent)

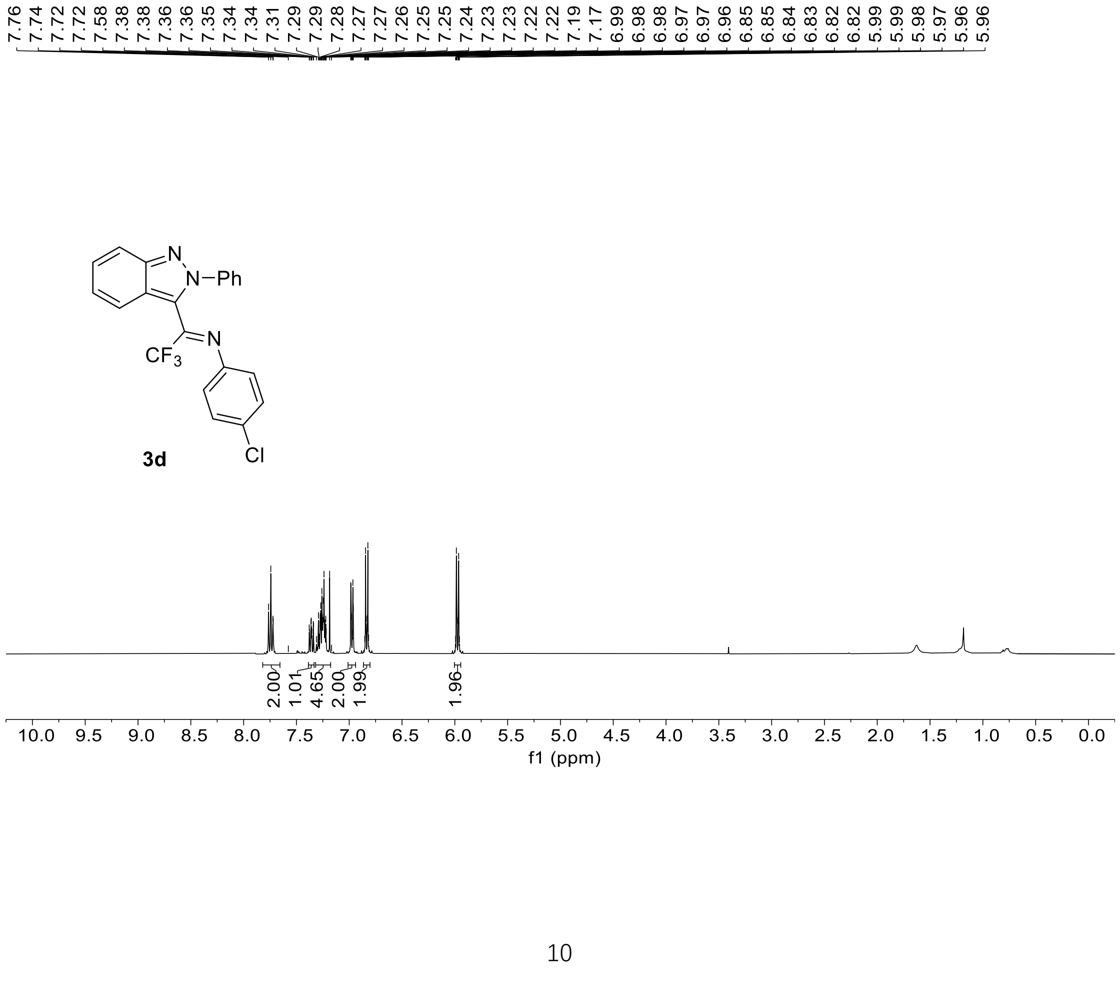

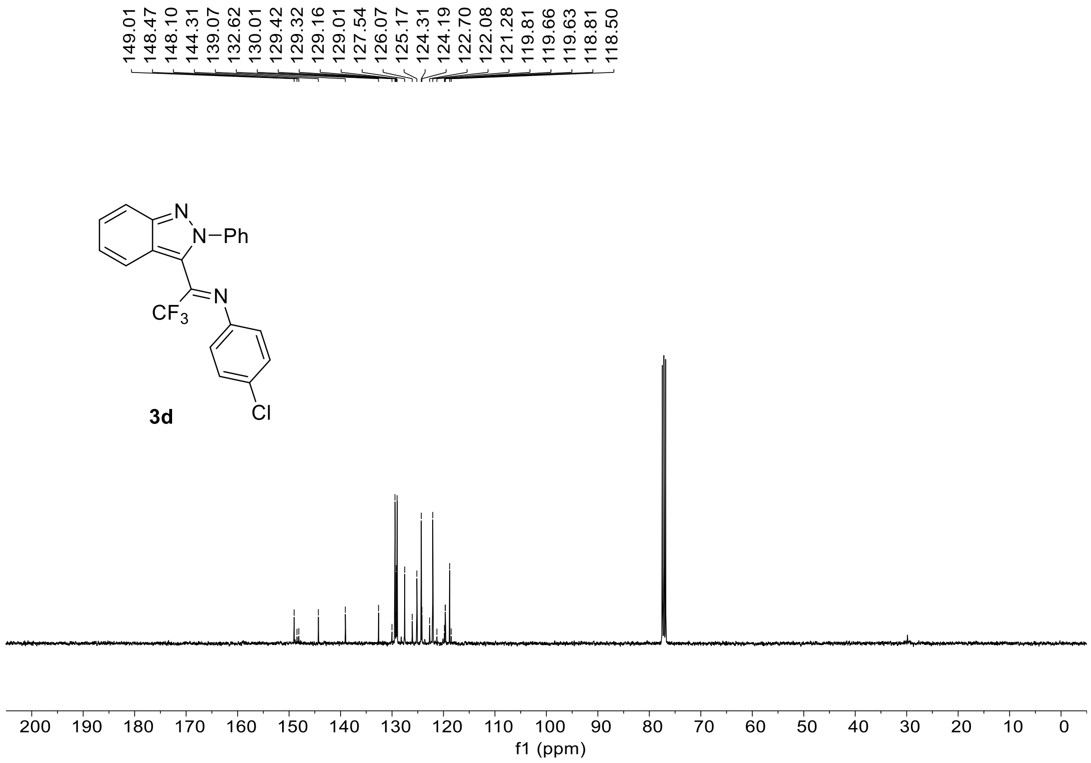
149.01
148.47
148.10
144.31
139.07
132.62
130.01
129.42
129.32
129.16
129.01
127.54
126.07
125.17
124.31
124.19
122.70
122.08
121.28
119.81
119.66
119.63
118.81
118.50
N
N−Ph
N
CF3
Cl
200 190 180 170 160 150 140 130 120 110 100 90 80 70 60 50 40 30 20 10 0
f1 (ppm)

3d

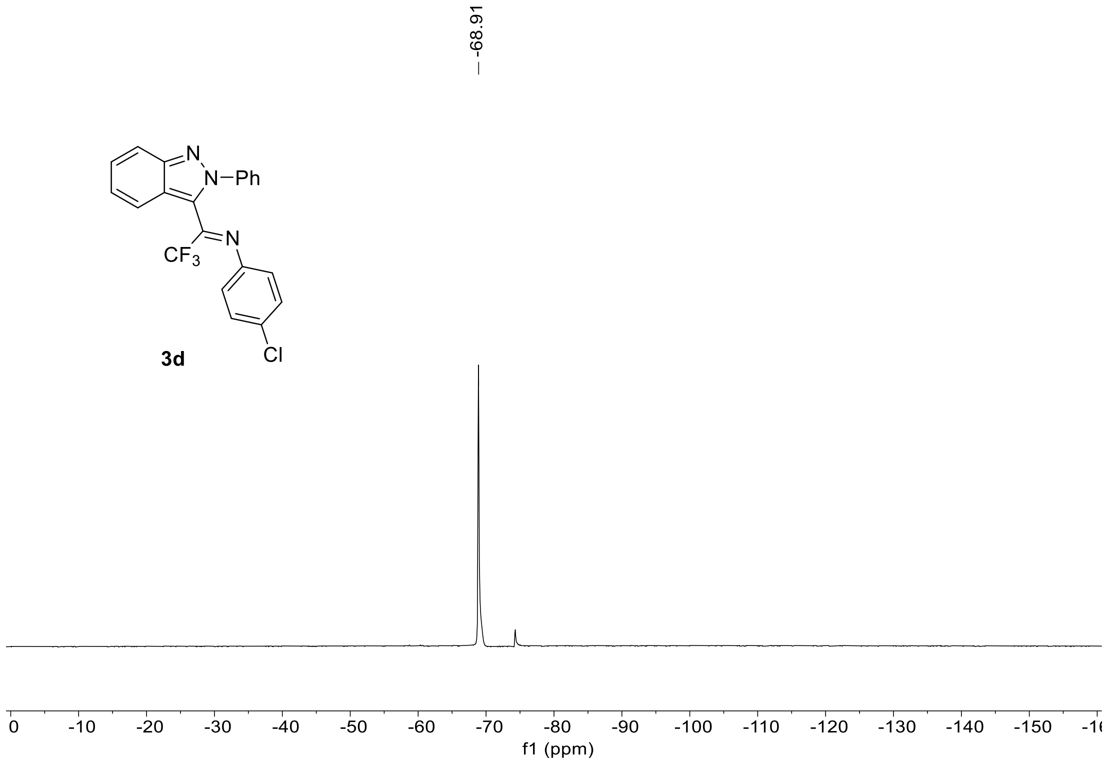
-68.91
N
N−Ph
N
CF3
Cl
0 -10 -20 -30 -40 -50 -60 -70 -80 -90 -100 -110 -120 -130 -140 -150
f1 (ppm)

3d

(5) ^{1}H NMR, ^{13}C NMR and ^{19}F NMR spectrum of **3e** (using $CDCl_3$ as solvent)

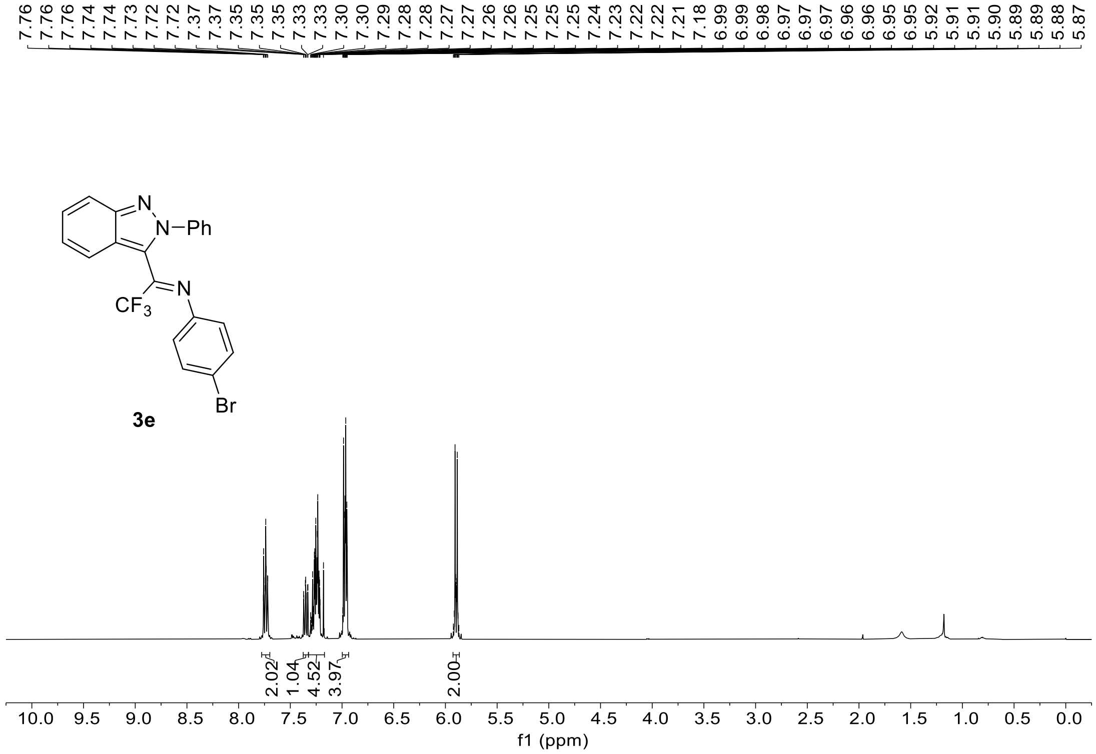

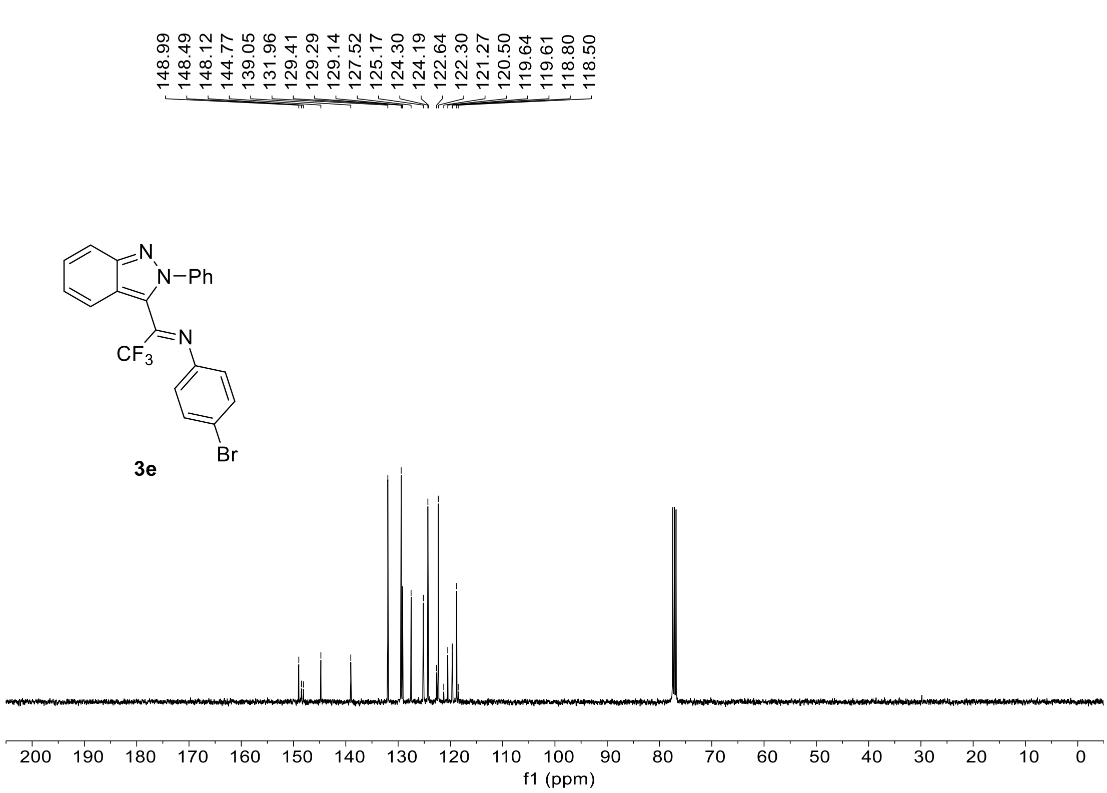

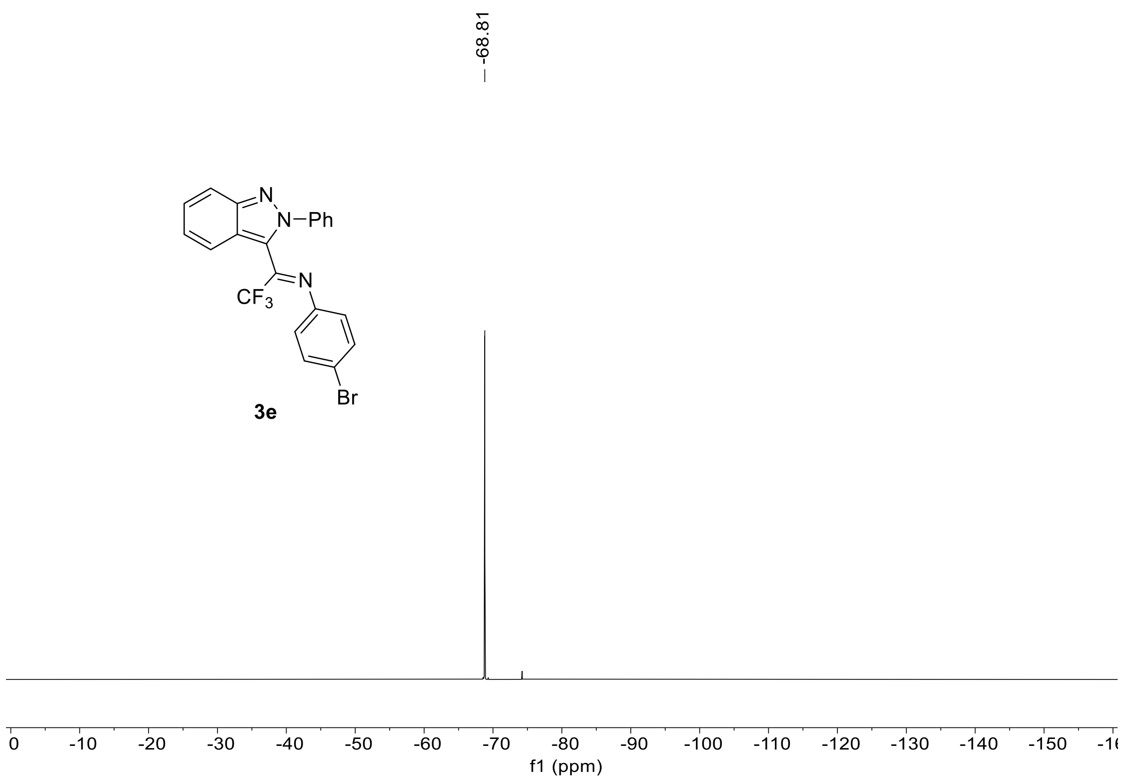

(6) ^{1}H NMR, ^{13}C NMR and ^{19}F NMR spectrum of **3f** (using $CDCl_3$ as solvent)

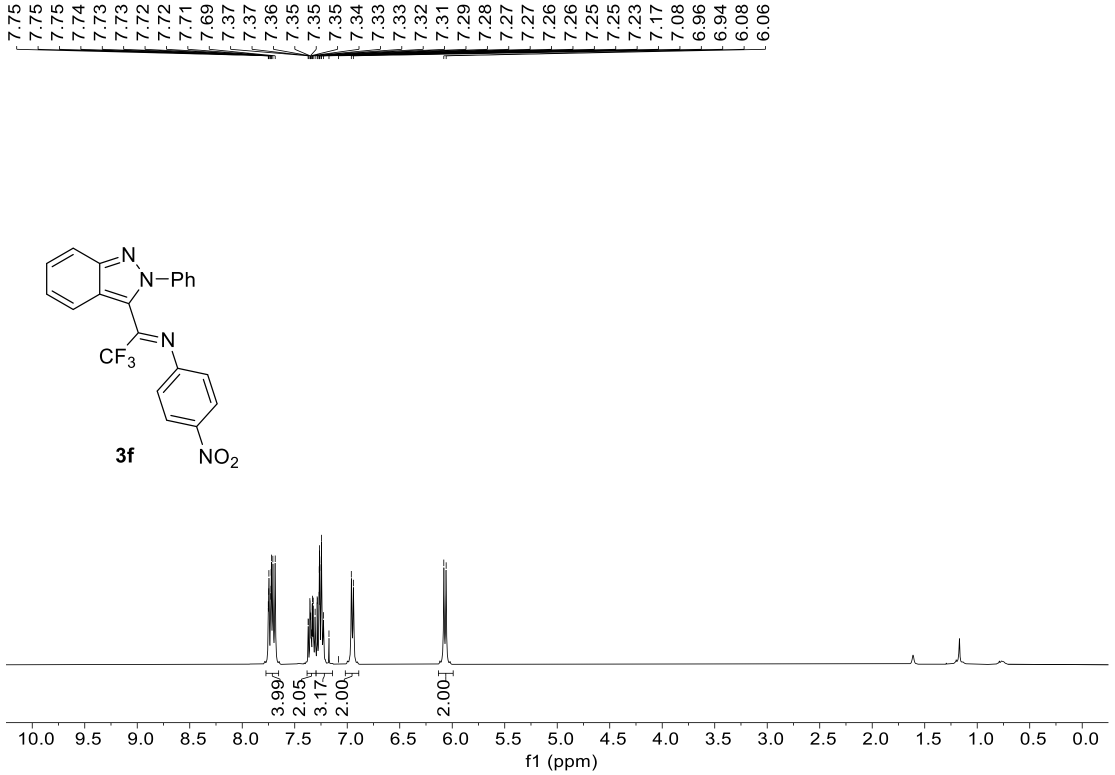

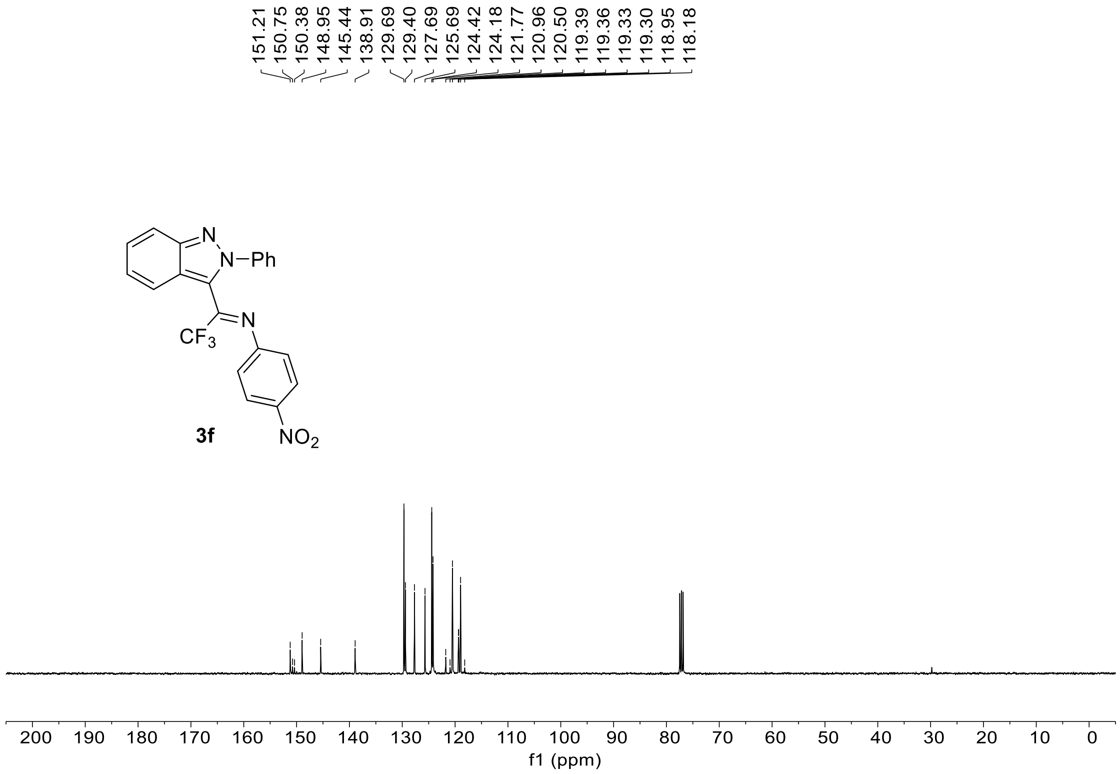
151.21
150.75
150.38
148.95
145.44
138.91
129.69
129.40
127.69
125.69
124.42
124.18
121.77
120.96
120.50
119.39
119.36
119.33
119.30
118.95
118.18
N
N−Ph
CF3
N
3f
NO2
200 190 180 170 160 150 140 130 120 110 100 90 80 70 60 50 40 30 20 10 0
f1 (ppm)

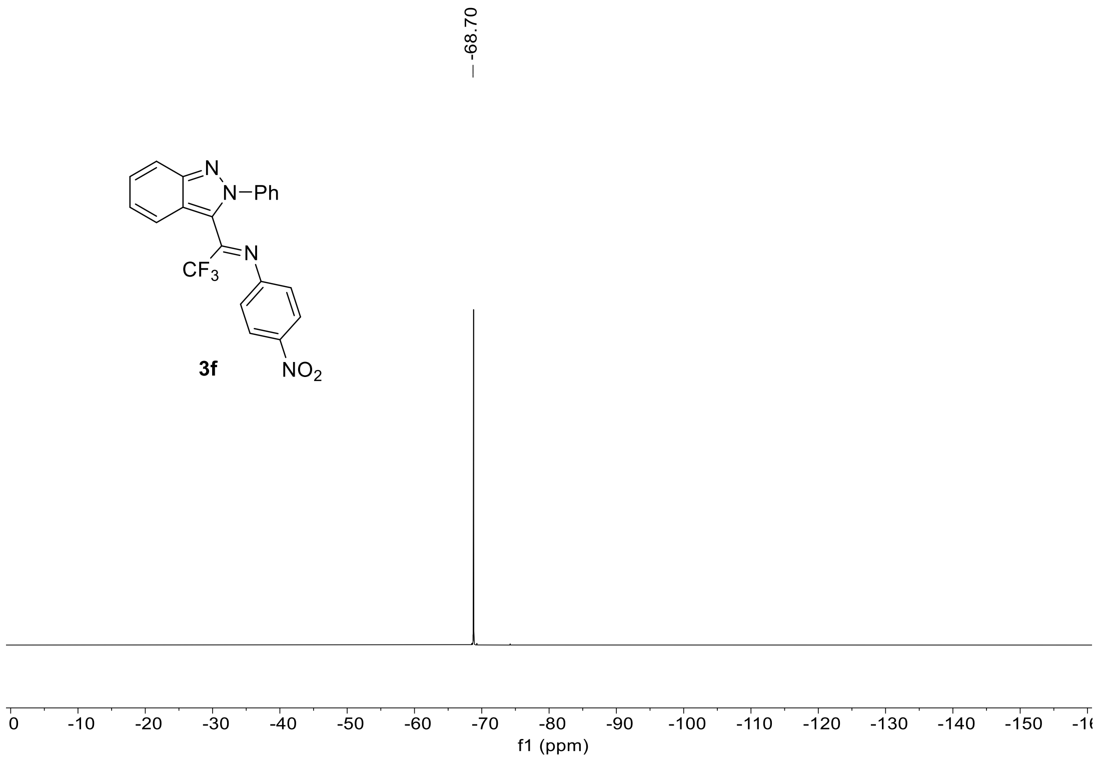
-68.70
N
N−Ph
CF3
N
3f
NO2
0 -10 -20 -30 -40 -50 -60 -70 -80 -90 -100 -110 -120 -130 -140 -150
f1 (ppm)

(7) ^{1}H NMR, ^{13}C NMR and ^{19}F NMR spectrum of **3g** (using $CDCl_3$ as solvent)

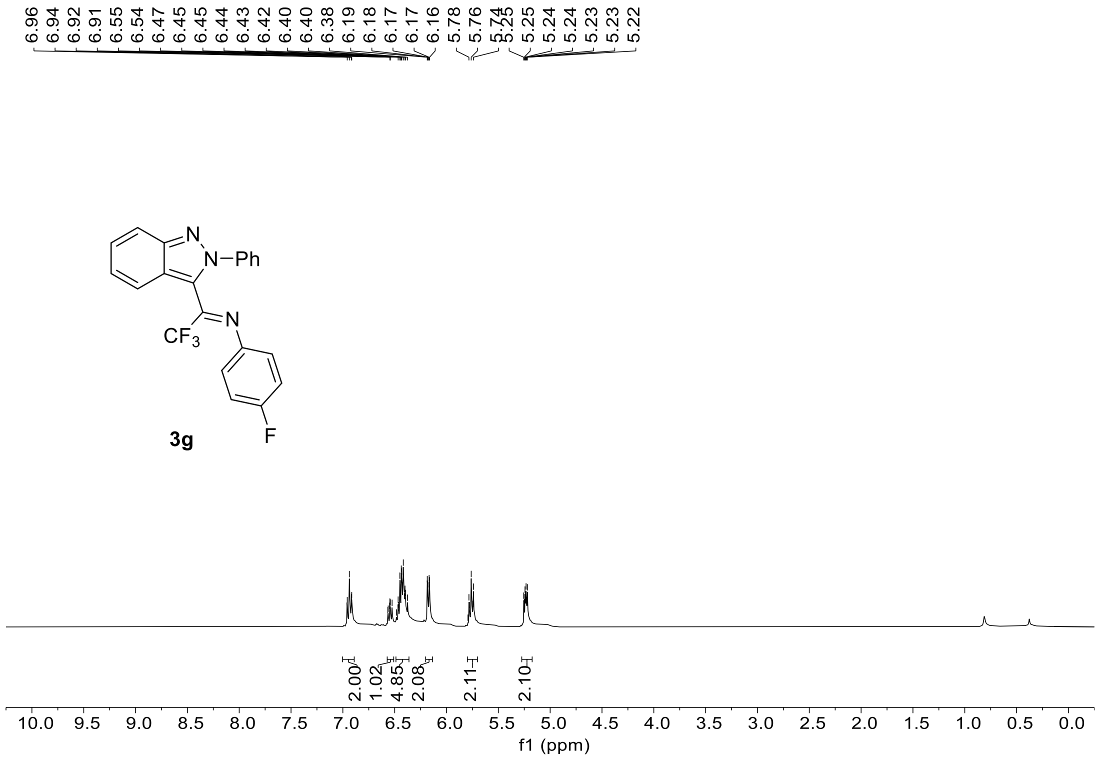

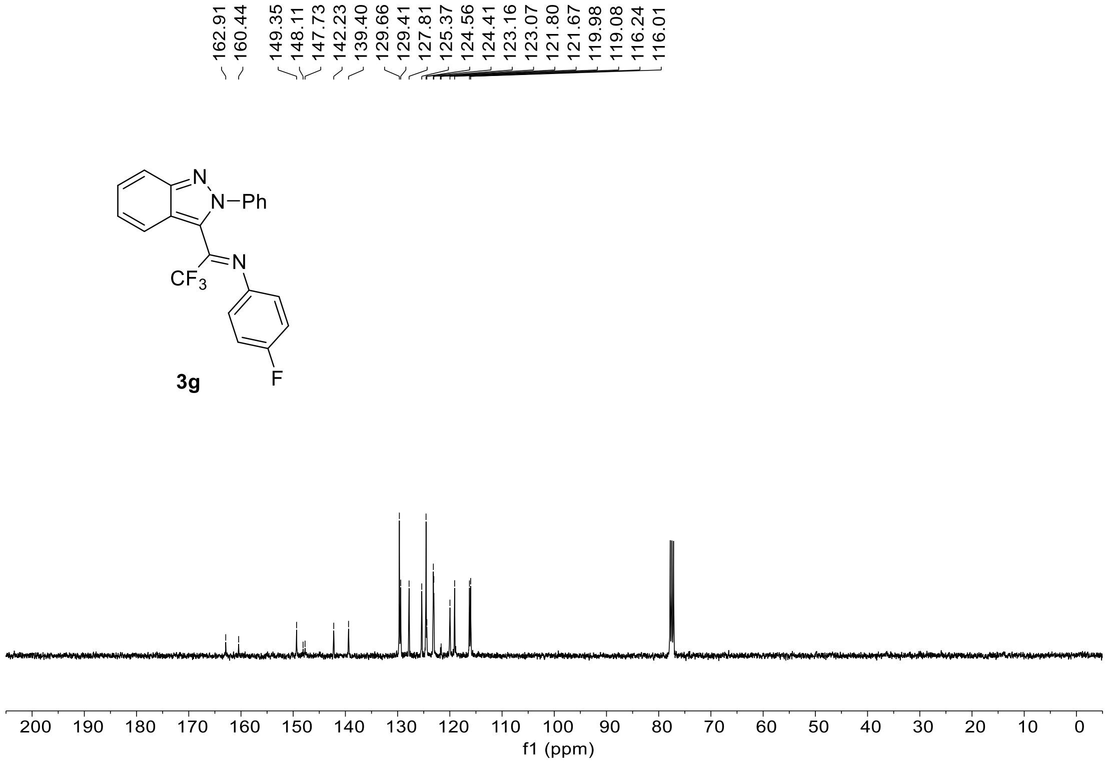

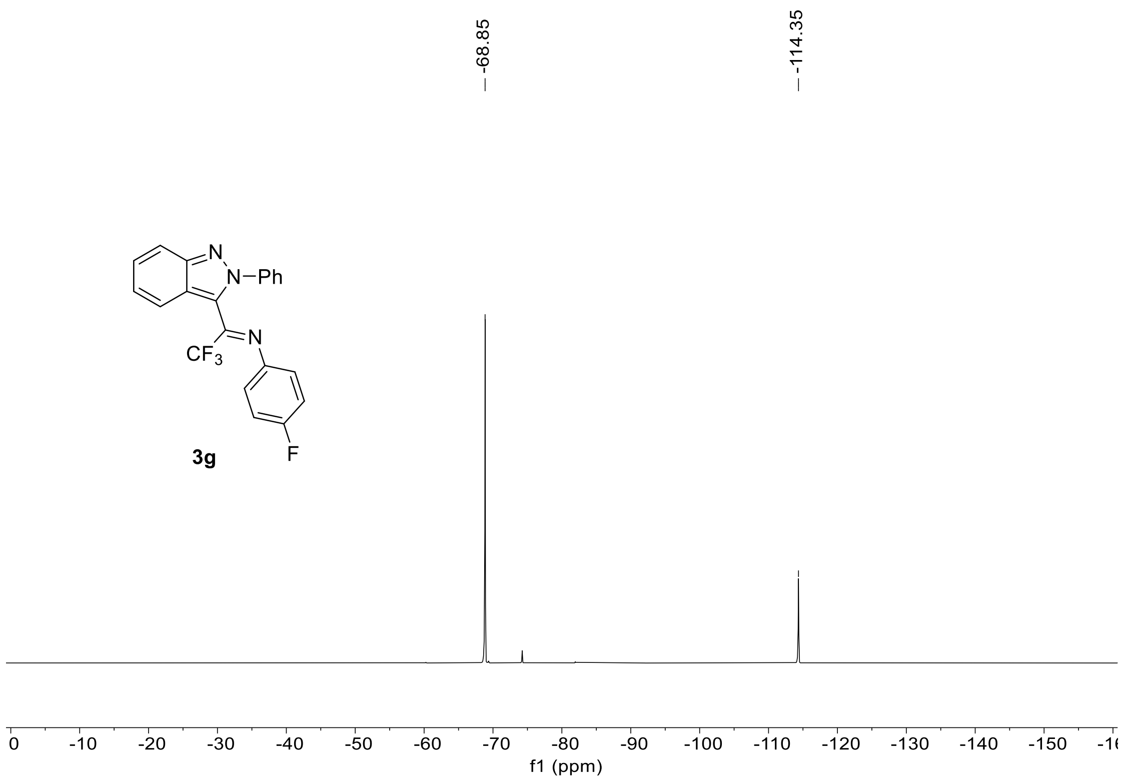

(8) ^{1}H NMR, ^{13}C NMR and ^{19}F NMR spectrum of **3h** (using $CDCl_3$ as solvent)

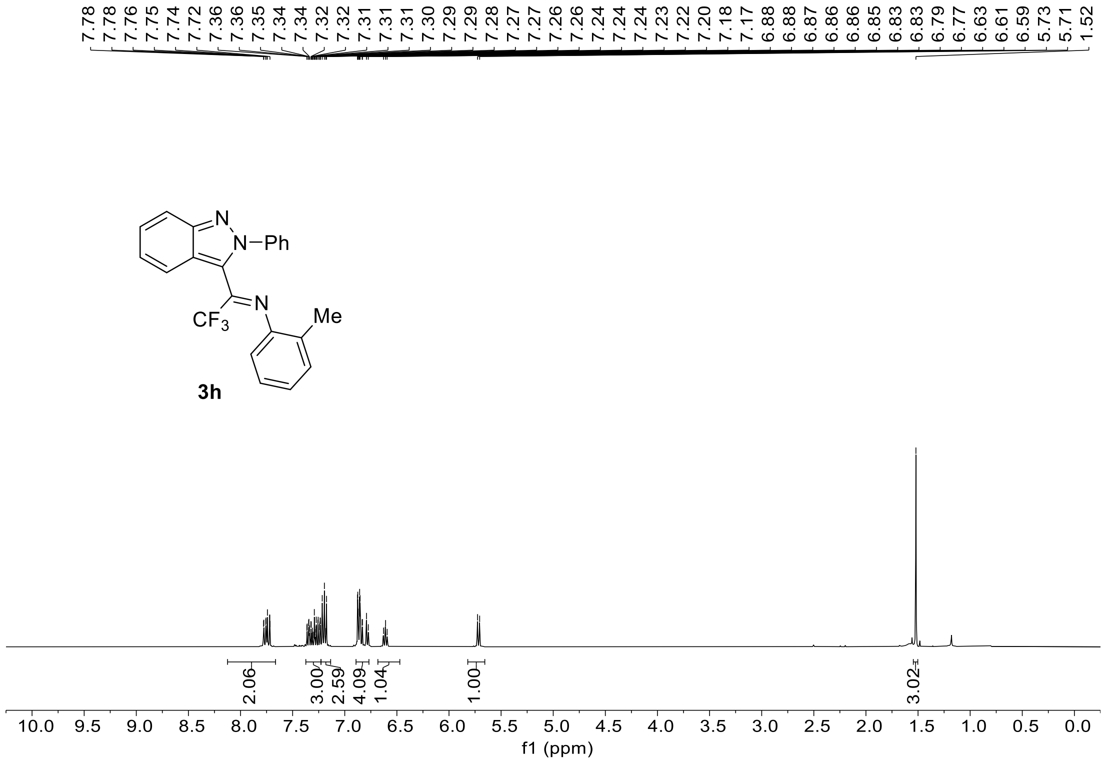

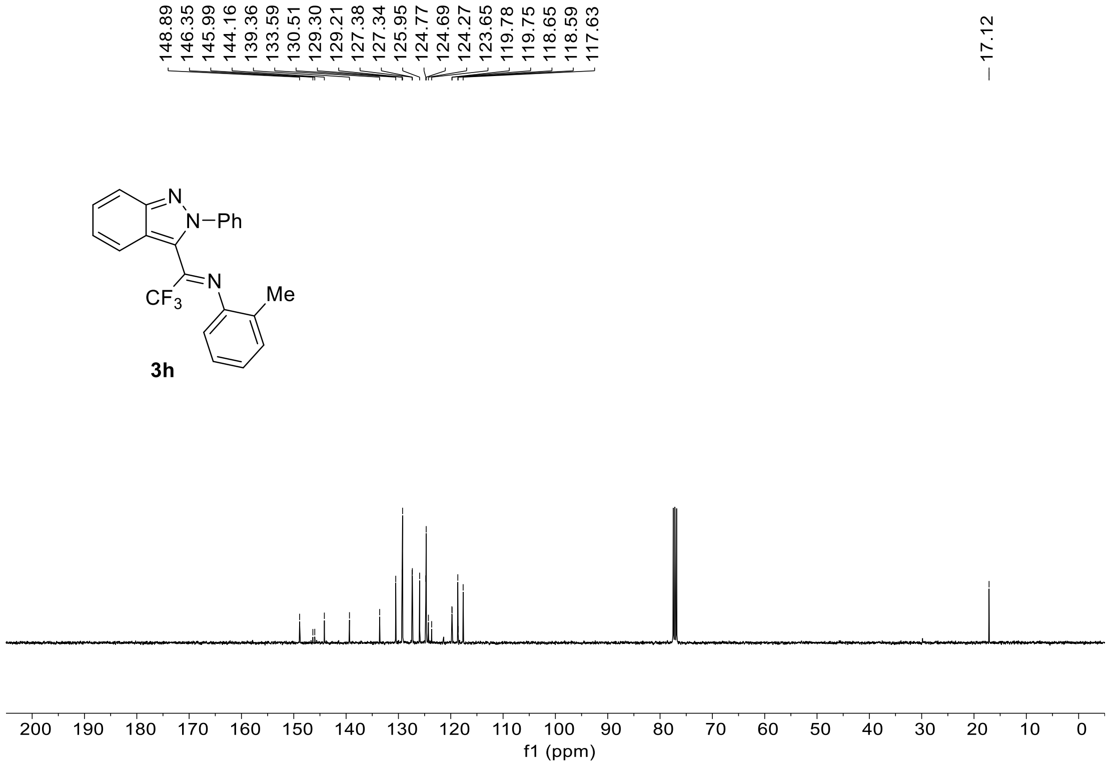
148.89
146.35
145.99
144.16
139.36
133.59
130.51
129.30
129.21
127.38
127.34
125.95
124.77
124.69
124.27
123.65
119.78
119.75
118.65
118.59
117.63
17.12
N
N−Ph
CF3
N
Me
3h
200 190 180 170 160 150 140 130 120 110 100 90 80 70 60 50 40 30 20 10 0
f1 (ppm)

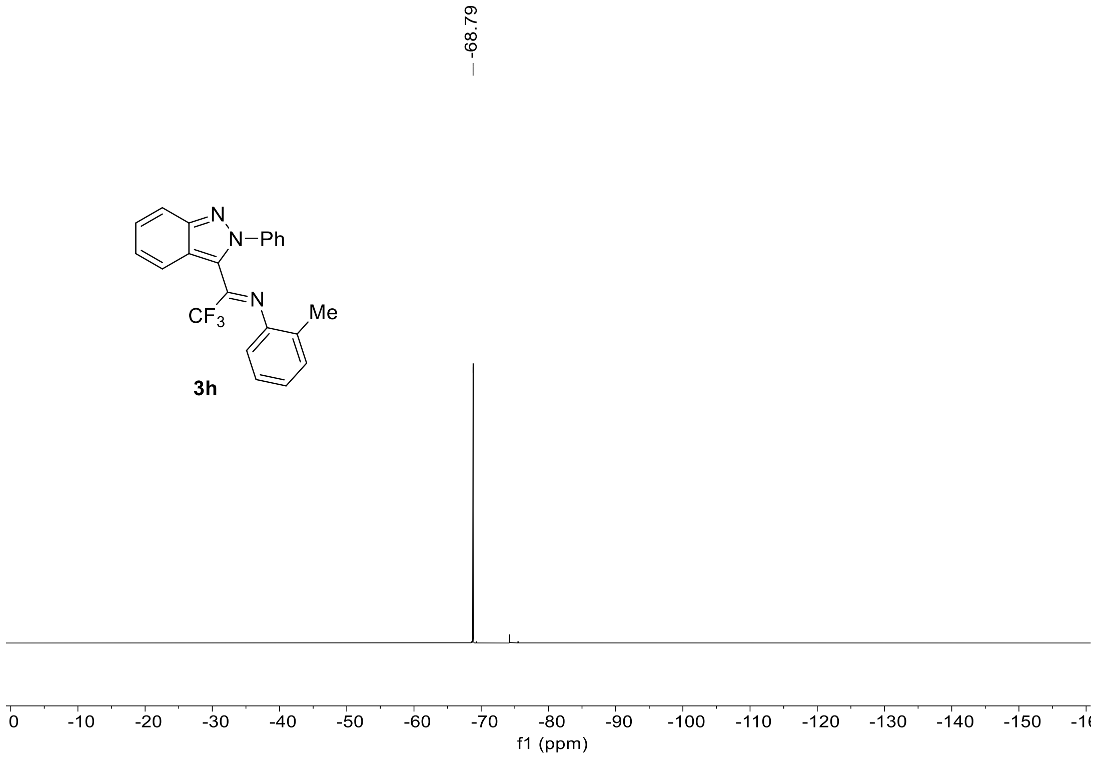
-68.79
N
N−Ph
CF3
N
Me
3h
0 -10 -20 -30 -40 -50 -60 -70 -80 -90 -100 -110 -120 -130 -140 -150
f1 (ppm)

(9) ^{1}H NMR, ^{13}C NMR and ^{19}F NMR spectrum of **3i** (using $CDCl_3$ as solvent)

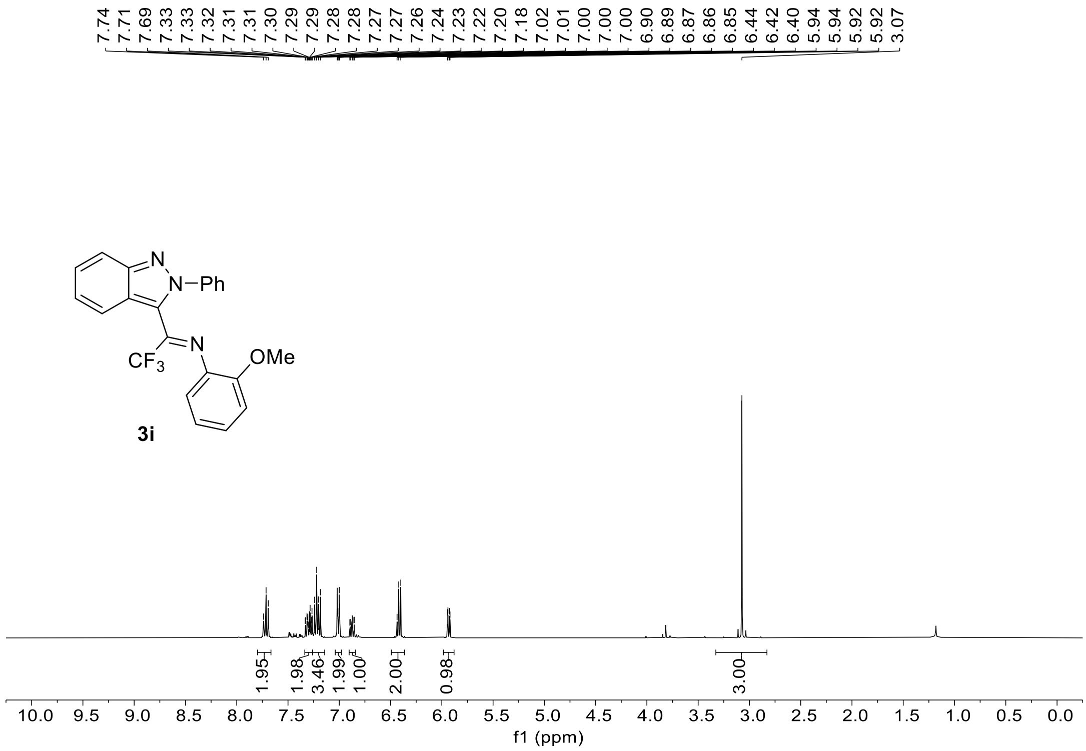

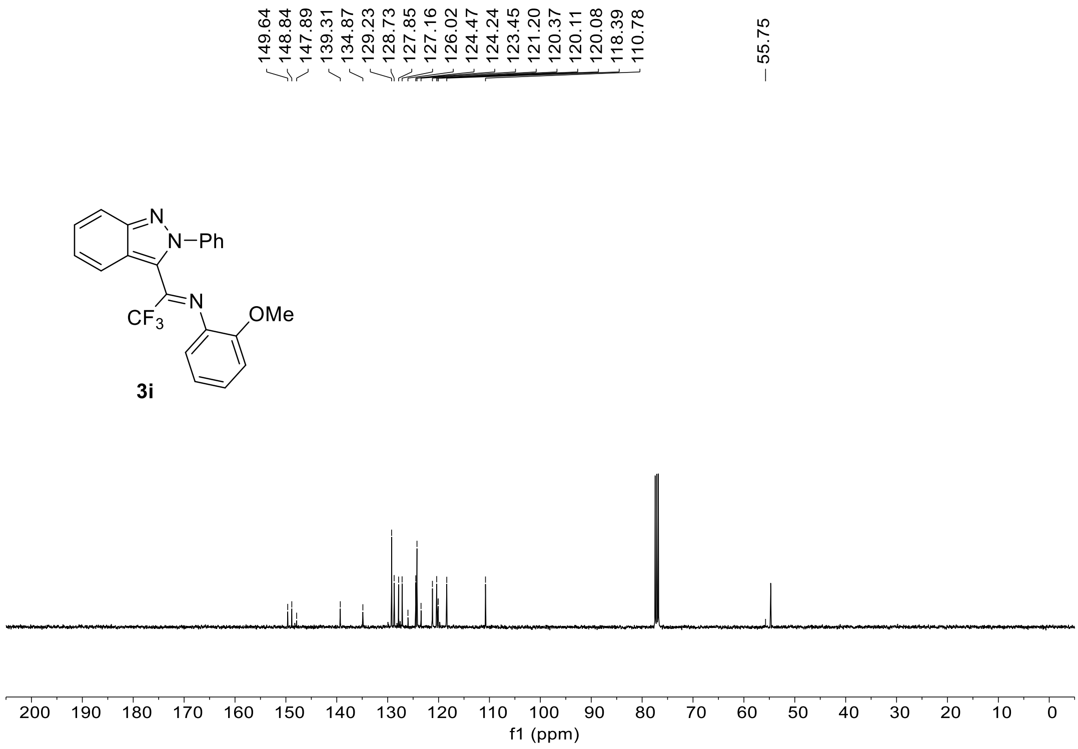

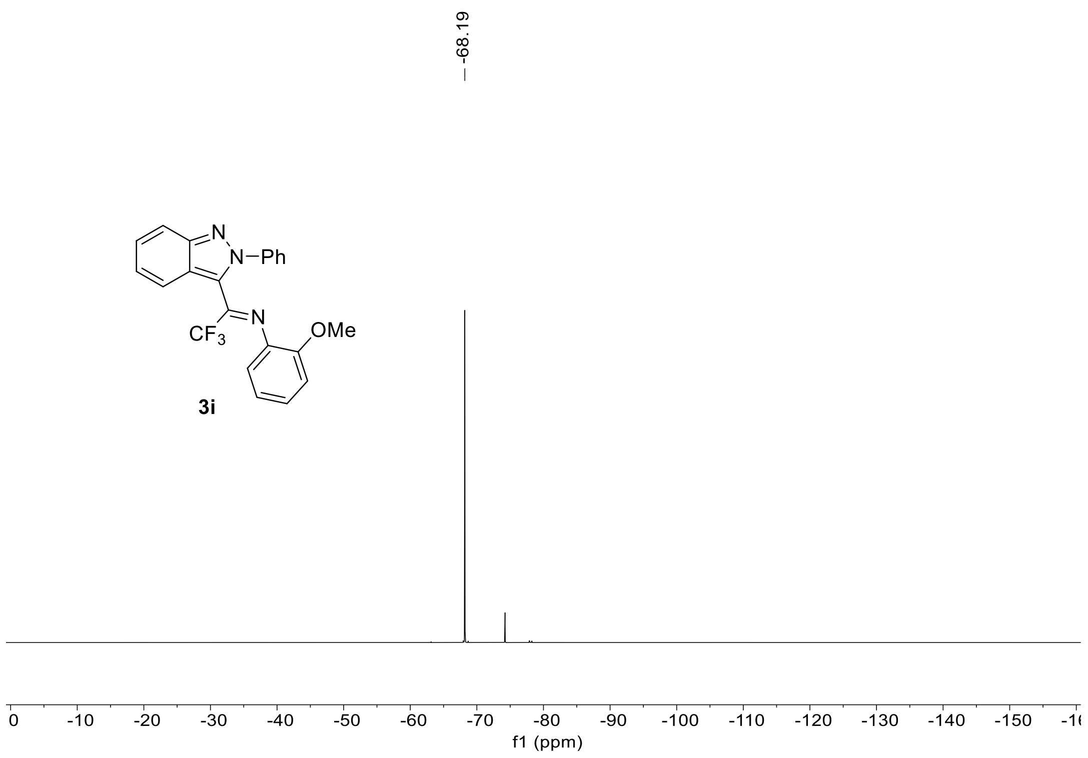

(10) ^{1}H NMR, ^{13}C NMR and ^{19}F NMR spectrum of **3j** (using $CDCl_3$ as solvent)

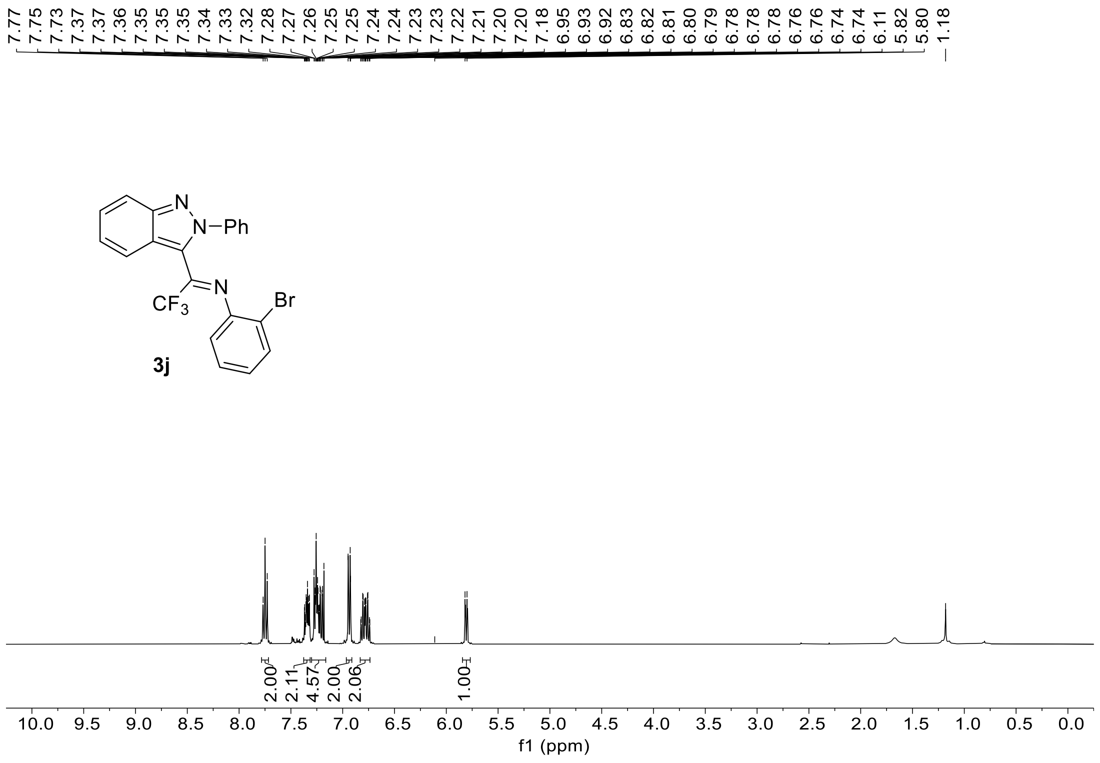

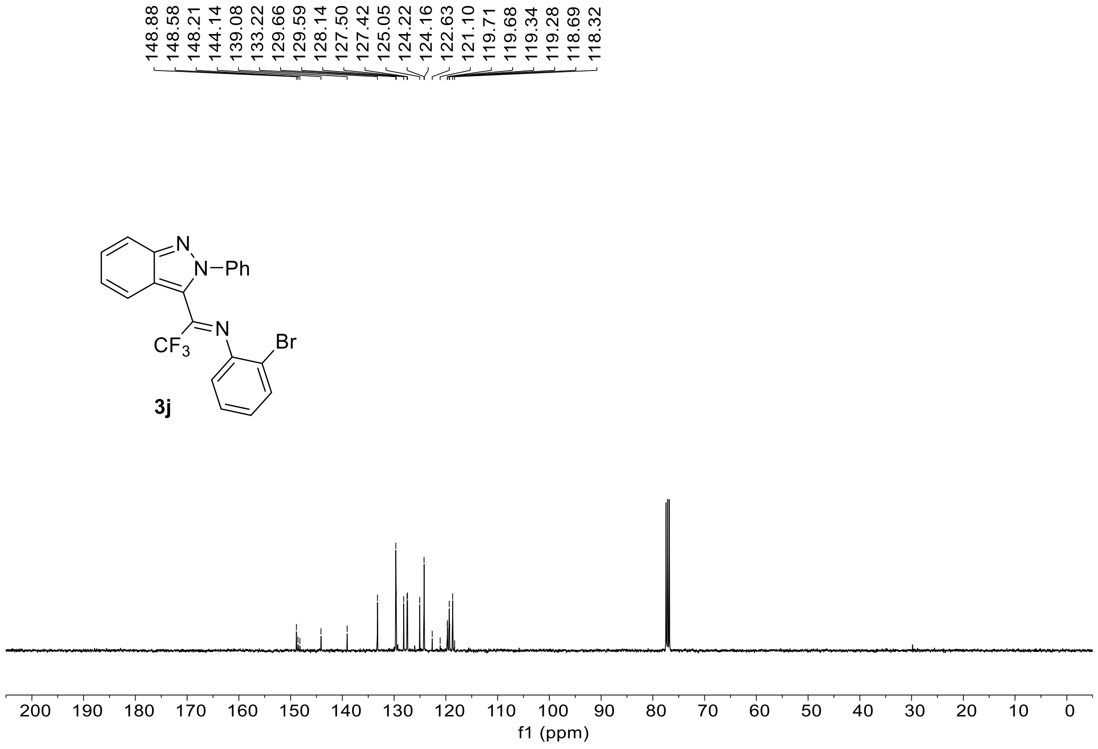
148.88
148.58
148.21
144.14
139.08
133.22
129.66
129.59
128.14
127.50
127.42
125.05
124.22
124.16
122.63
121.10
119.71
119.68
119.34
119.28
118.69
118.32
N
N−Ph
CF3
N
Br
3j
200 190 180 170 160 150 140 130 120 110 100 90 80 70 60 50 40 30 20 10 0
f1 (ppm)

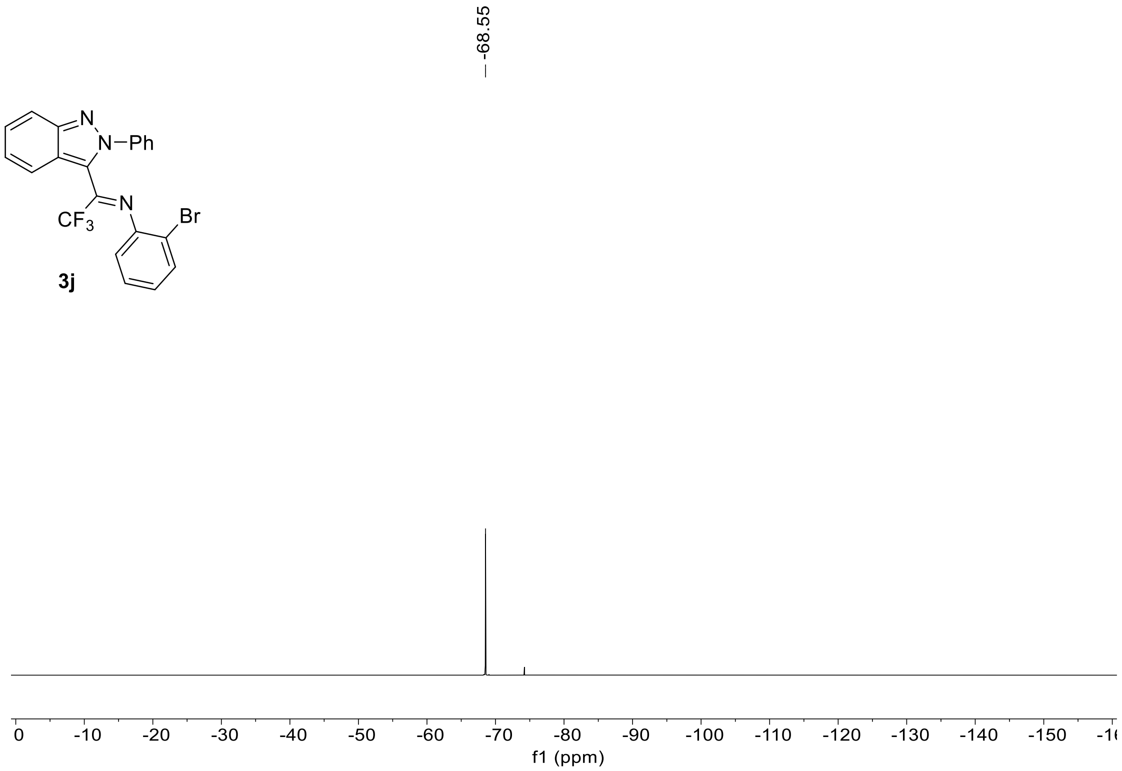
-68.55
N
N−Ph
CF3
N
Br
3j
0 -10 -20 -30 -40 -50 -60 -70 -80 -90 -100 -110 -120 -130 -140 -150
f1 (ppm)

(11) ^{1}H NMR, ^{13}C NMR and ^{19}F NMR spectrum of **3k** (using $CDCl_3$ as solvent)

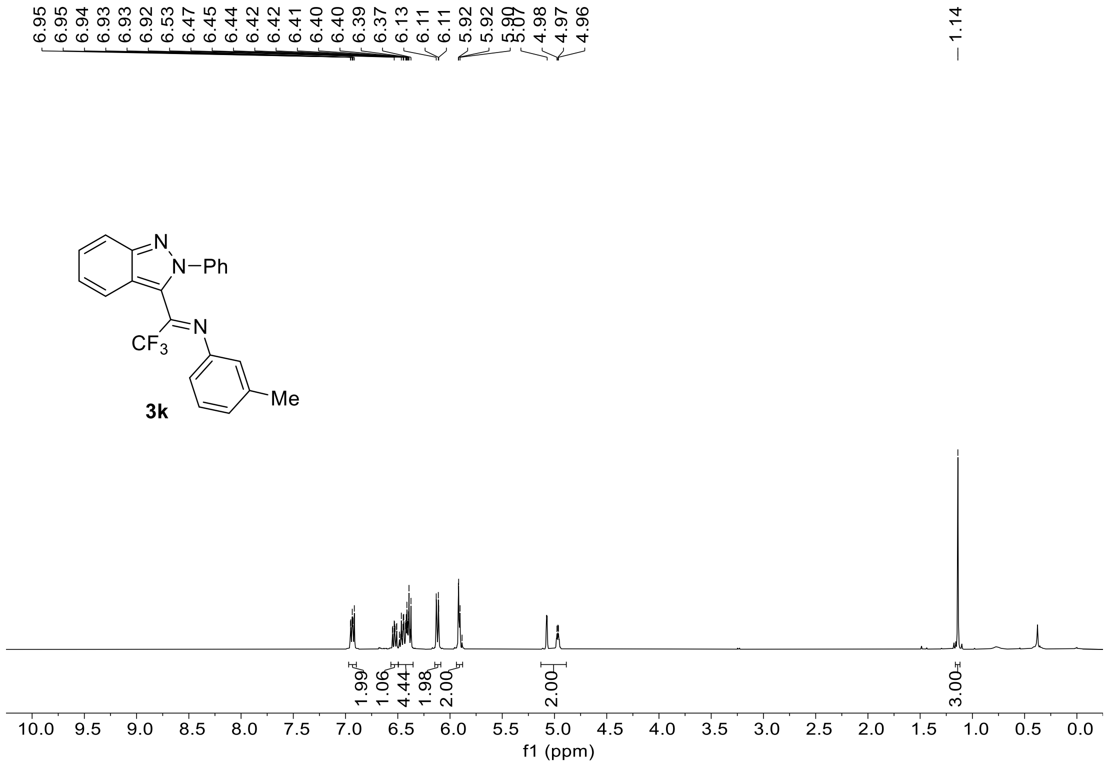

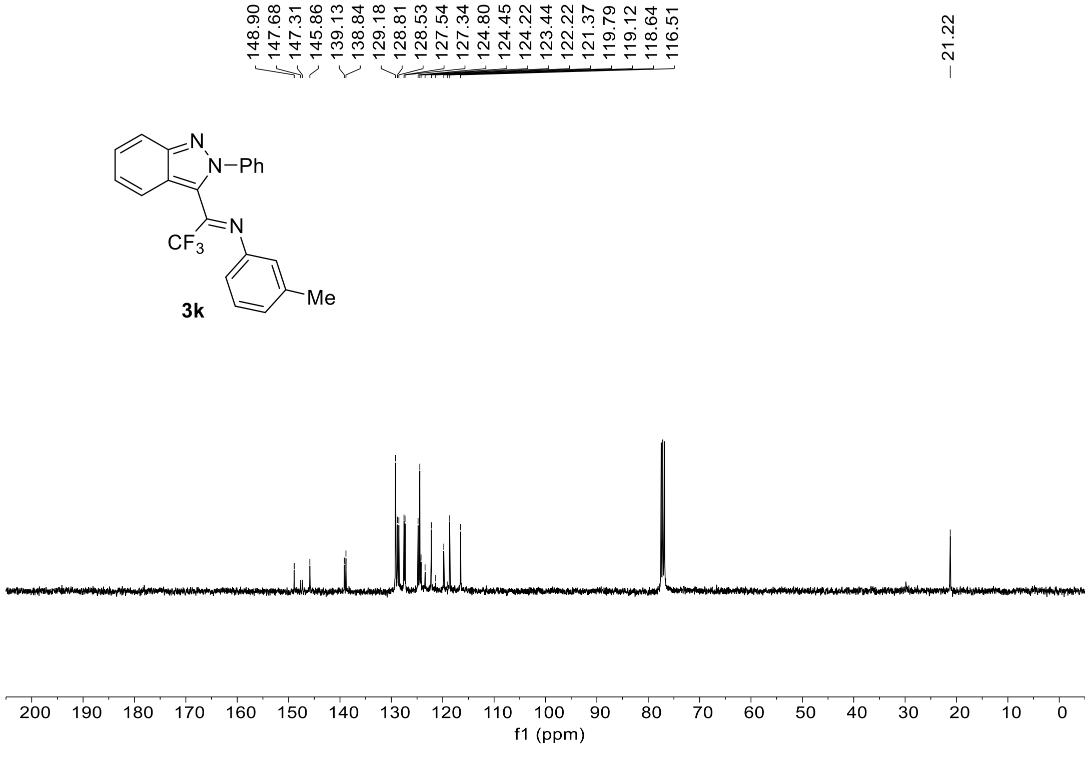

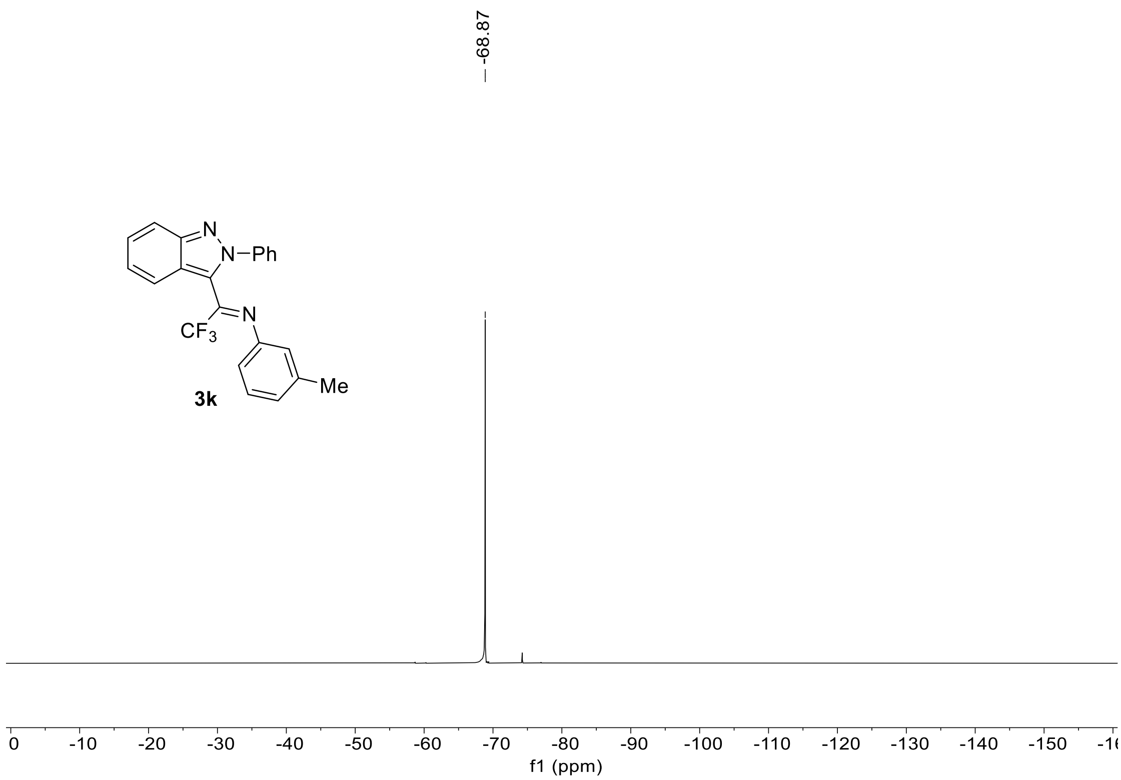

(12) ^{1}H NMR, ^{13}C NMR and ^{19}F NMR spectrum of **3l** (using $CDCl_3$ as solvent)

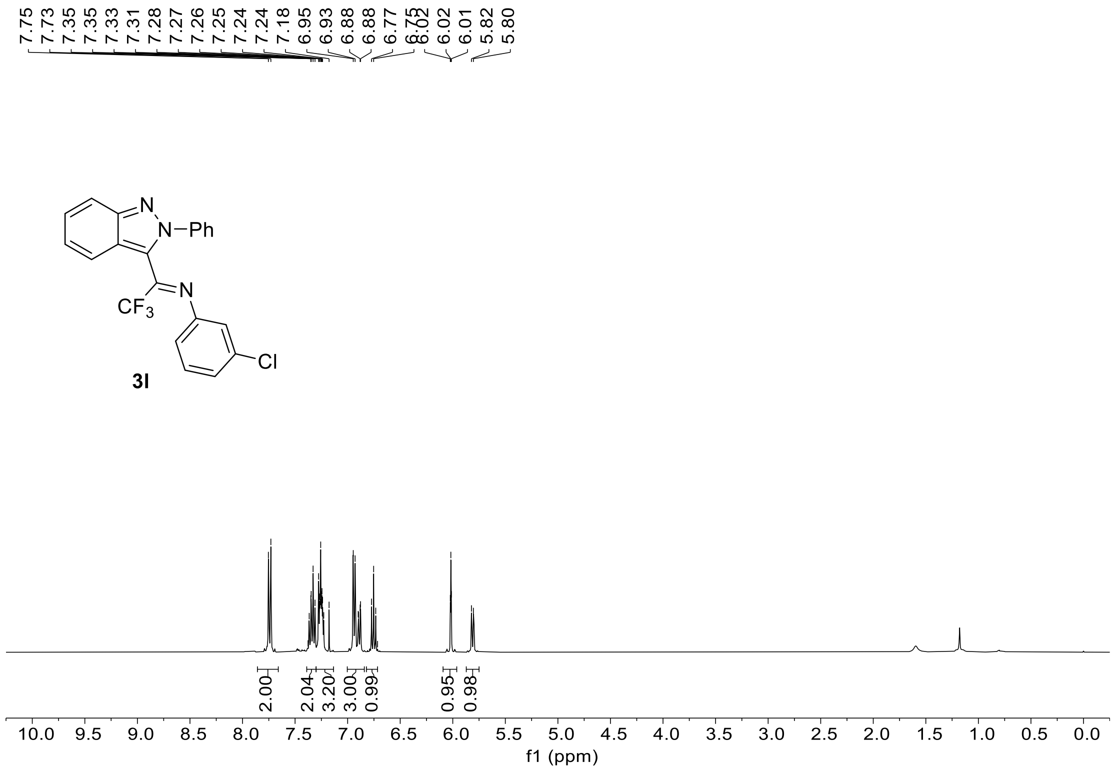

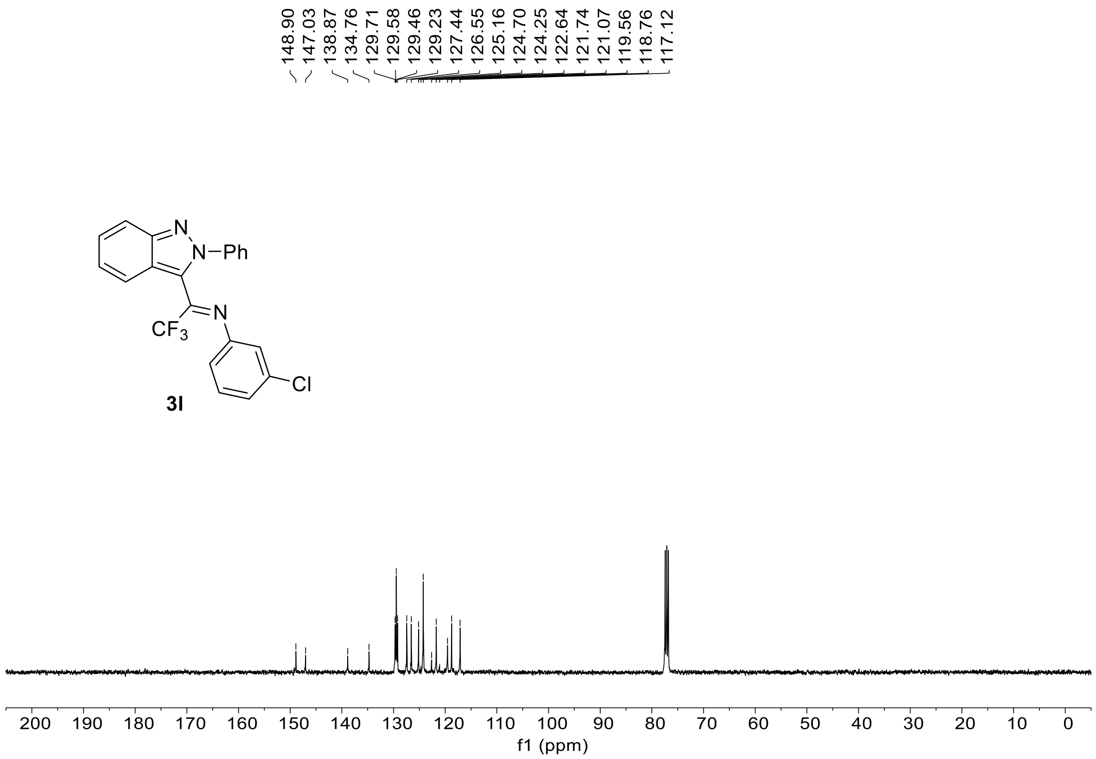
148.90
147.03
138.87
134.76
129.71
129.58
129.46
129.23
127.44
126.55
125.16
124.70
124.25
122.64
121.74
121.07
119.56
118.76
117.12
N
N−Ph
CF3
N
Cl
3l
200 190 180 170 160 150 140 130 120 110 100 90 80 70 60 50 40 30 20 10 0
f1 (ppm)

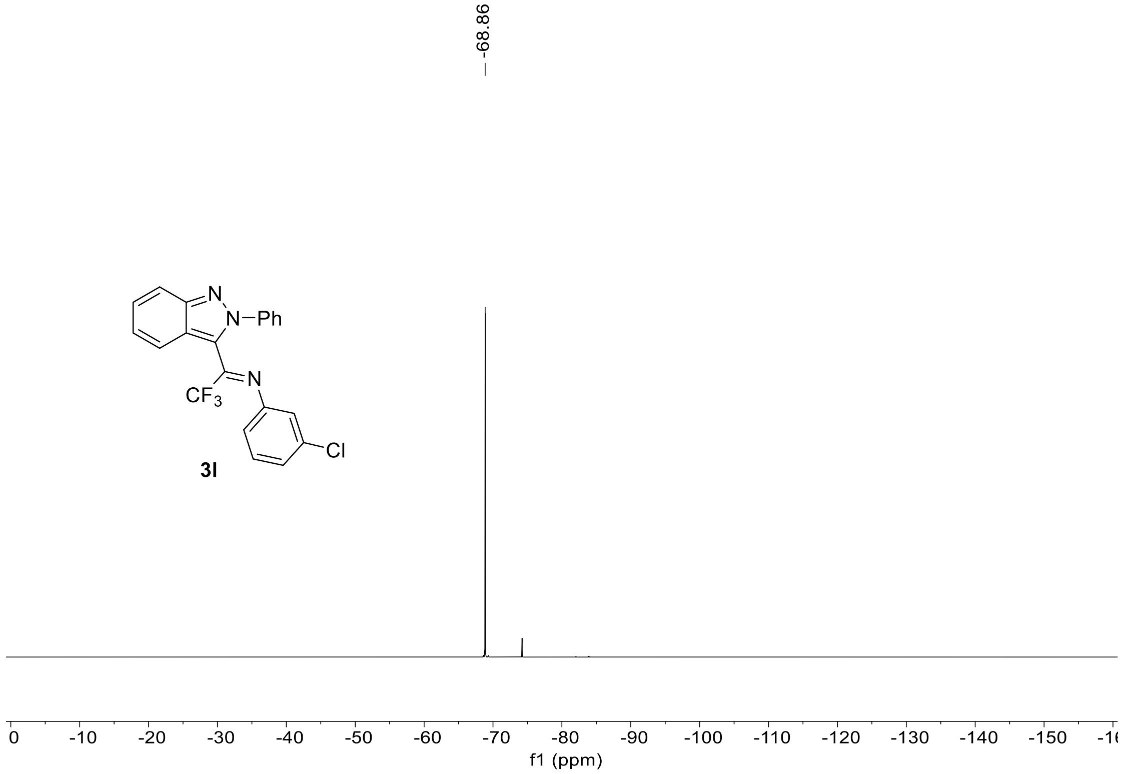
-68.86
N
N−Ph
CF3
N
Cl
3l
0 -10 -20 -30 -40 -50 -60 -70 -80 -90 -100 -110 -120 -130 -140 -150
f1 (ppm)

(13) ^{1}H NMR, ^{13}C NMR and ^{19}F NMR spectrum of **3m** (using $CDCl_3$ as solvent)

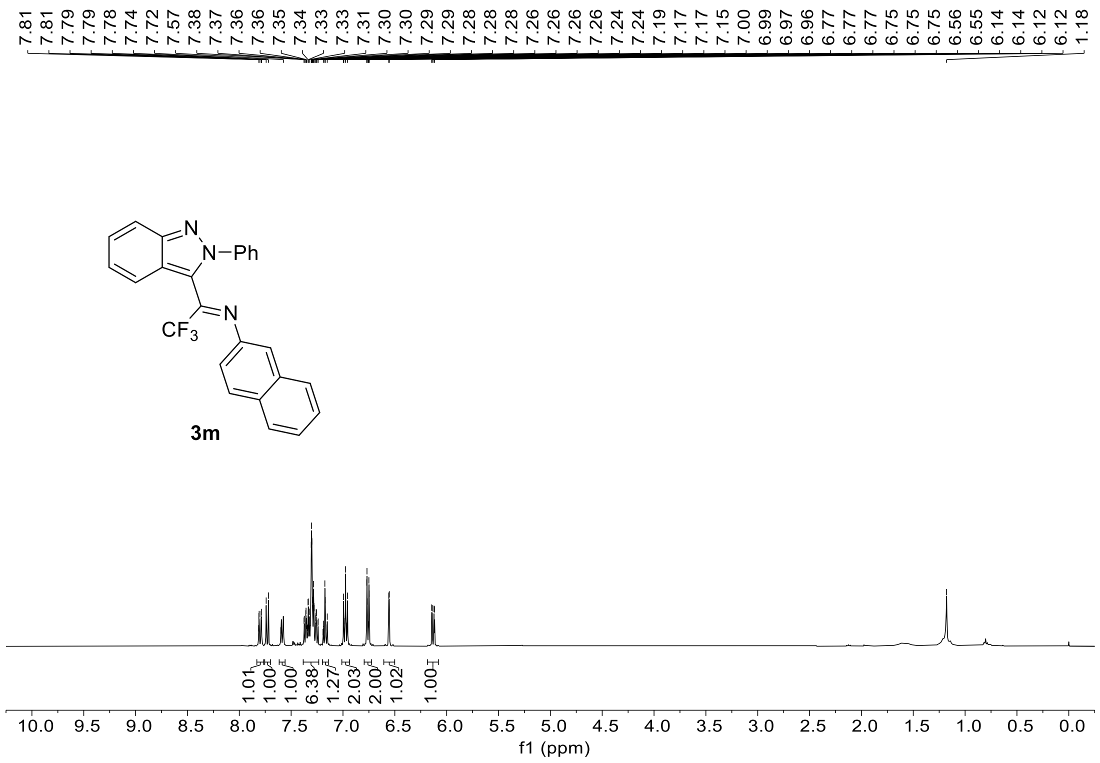

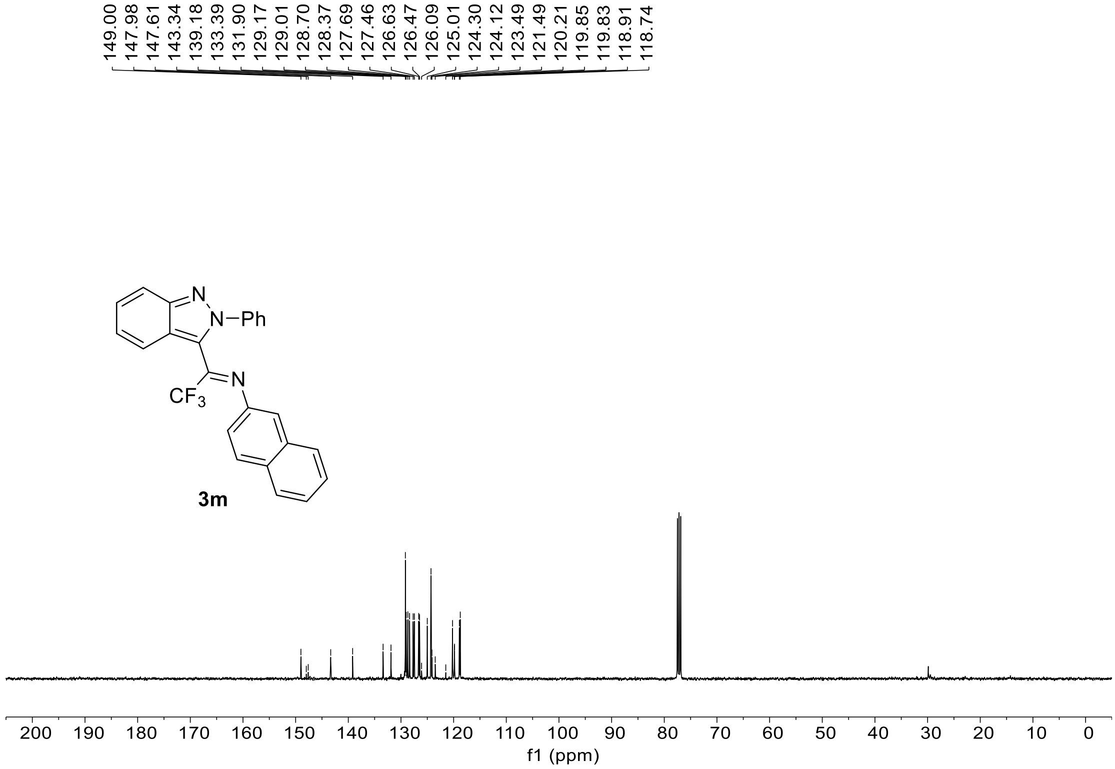

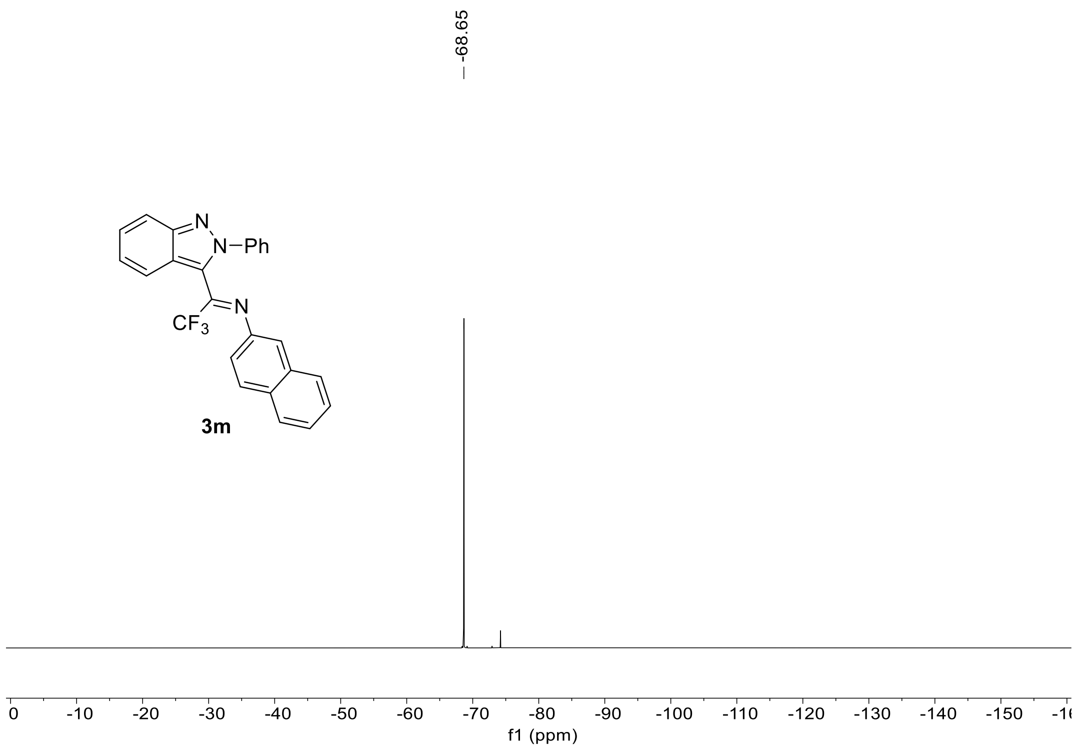

(14) ^{1}H NMR, ^{13}C NMR and ^{19}F NMR spectrum of **3n** (using $CDCl_3$ as solvent)

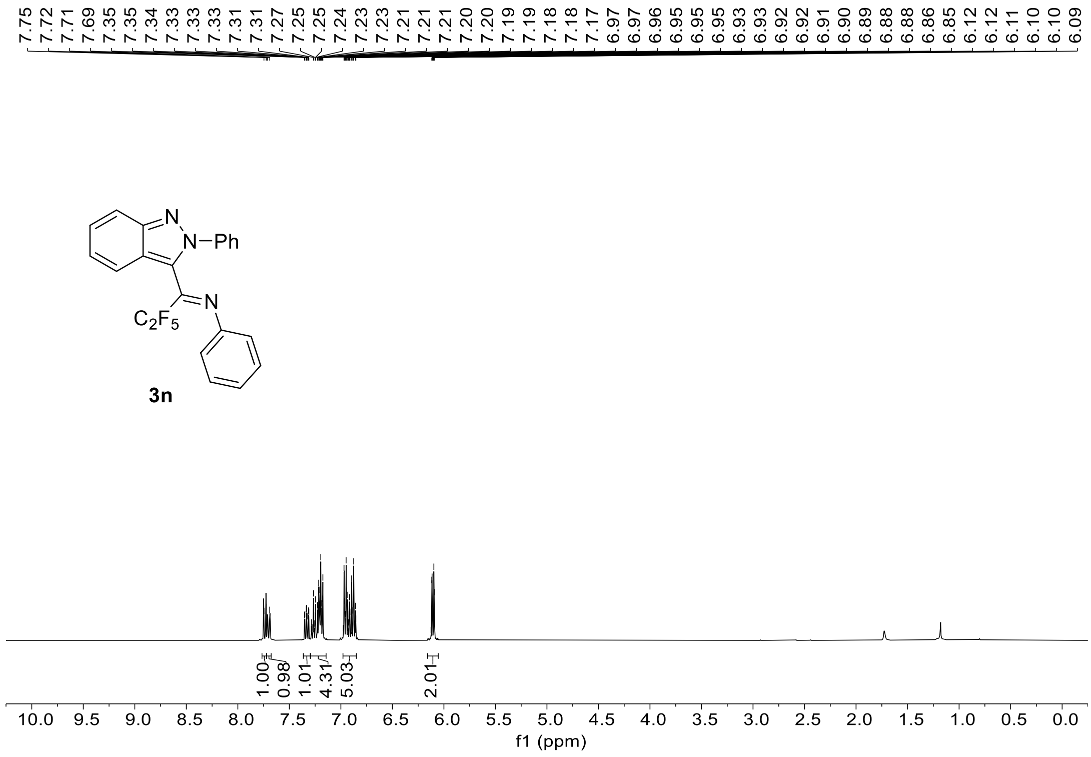

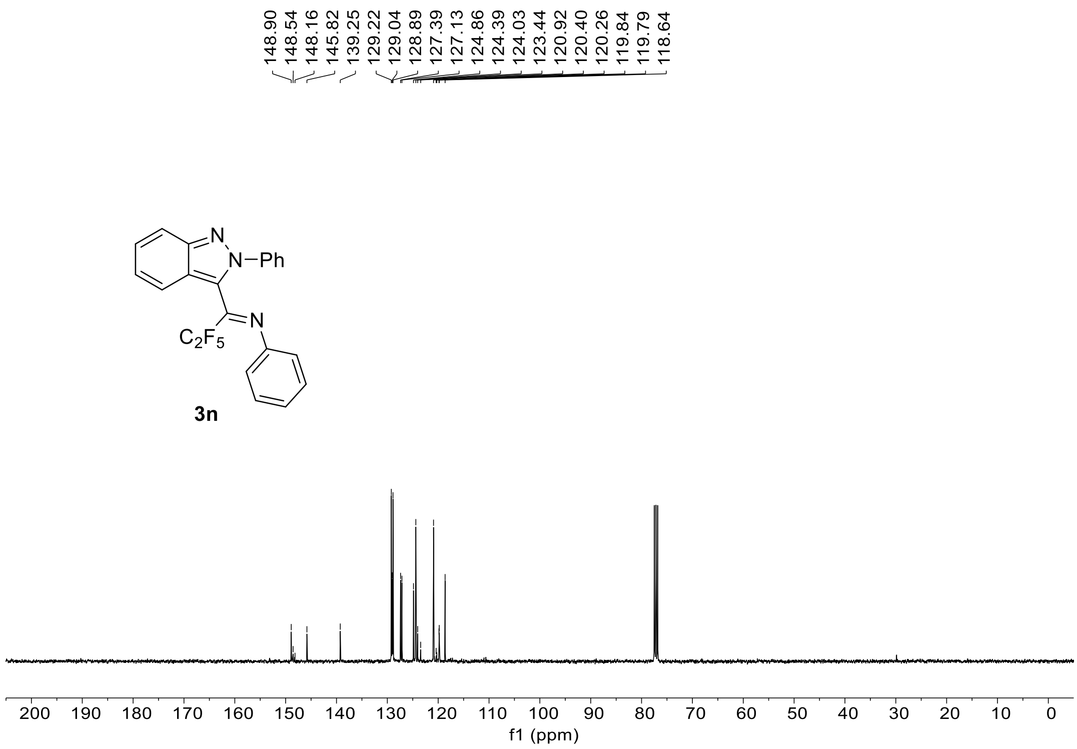
148.90
148.54
148.16
145.82
139.25
129.22
129.04
128.89
127.39
127.13
124.86
124.39
124.03
123.44
120.92
120.40
120.26
119.84
119.79
118.64
N
N−Ph
N
C_2F_5
3n
200 190 180 170 160 150 140 130 120 110 100 90 80 70 60 50 40 30 20 10 0
f1 (ppm)

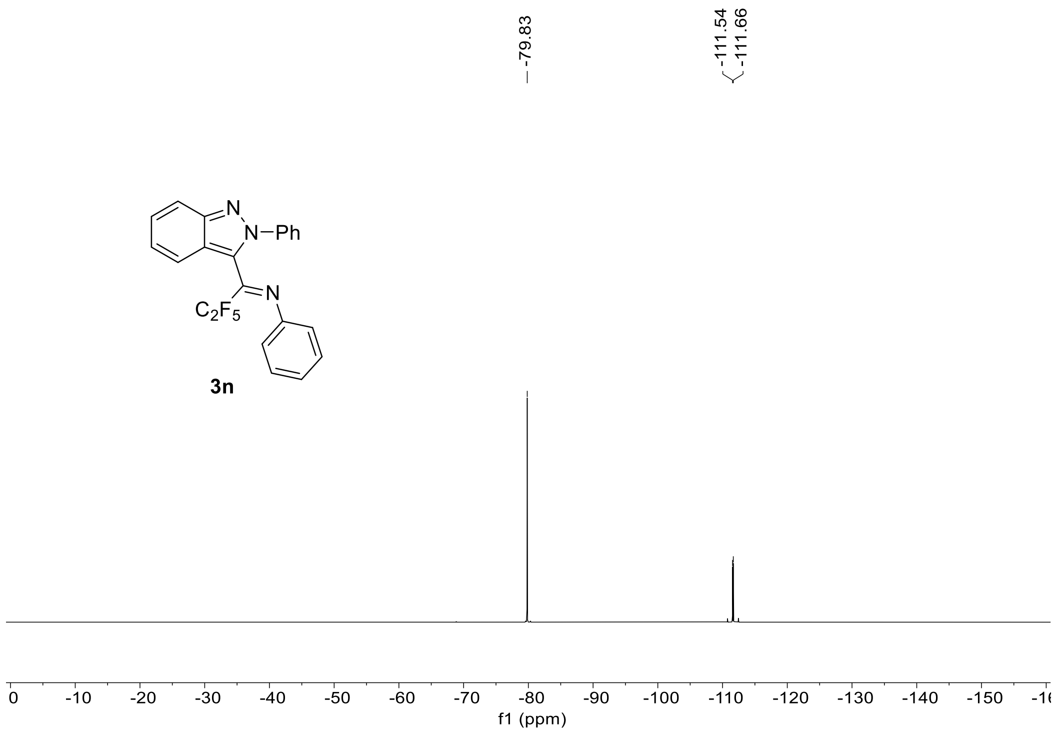
-79.83
-111.54
-111.66
N
N−Ph
N
C_2F_5
3n
0 -10 -20 -30 -40 -50 -60 -70 -80 -90 -100 -110 -120 -130 -140 -150
f1 (ppm)

(15) ^{1}H NMR, ^{13}C NMR and ^{19}F NMR spectrum of **3o** (using $CDCl_3$ as solvent)

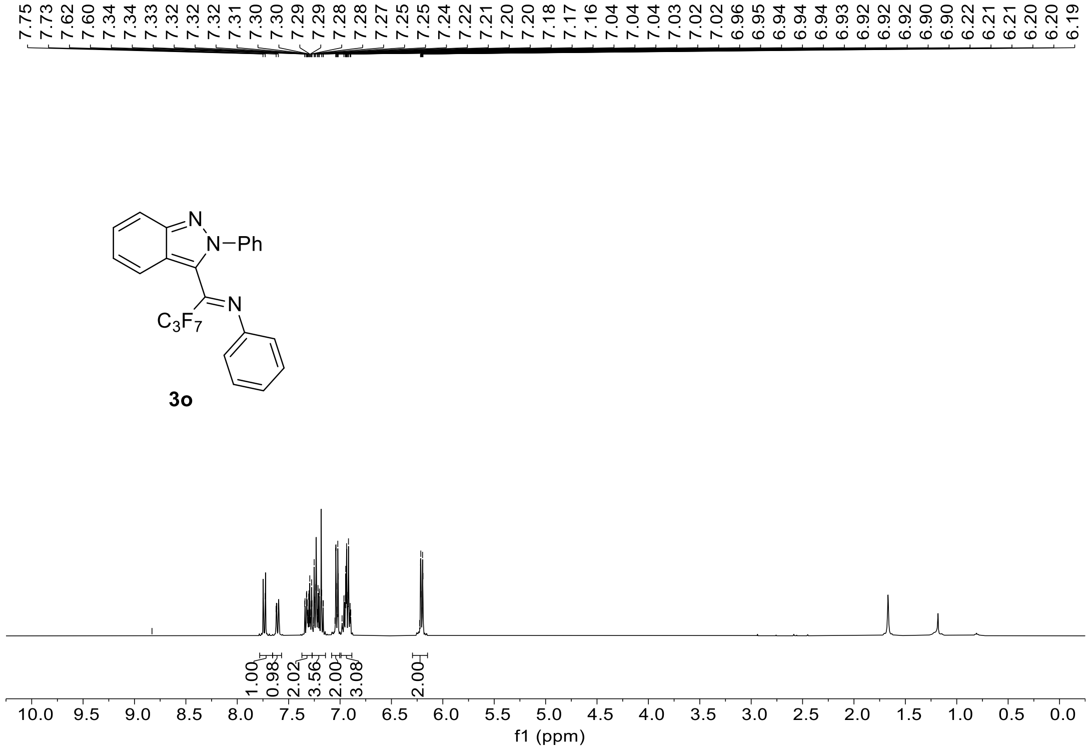

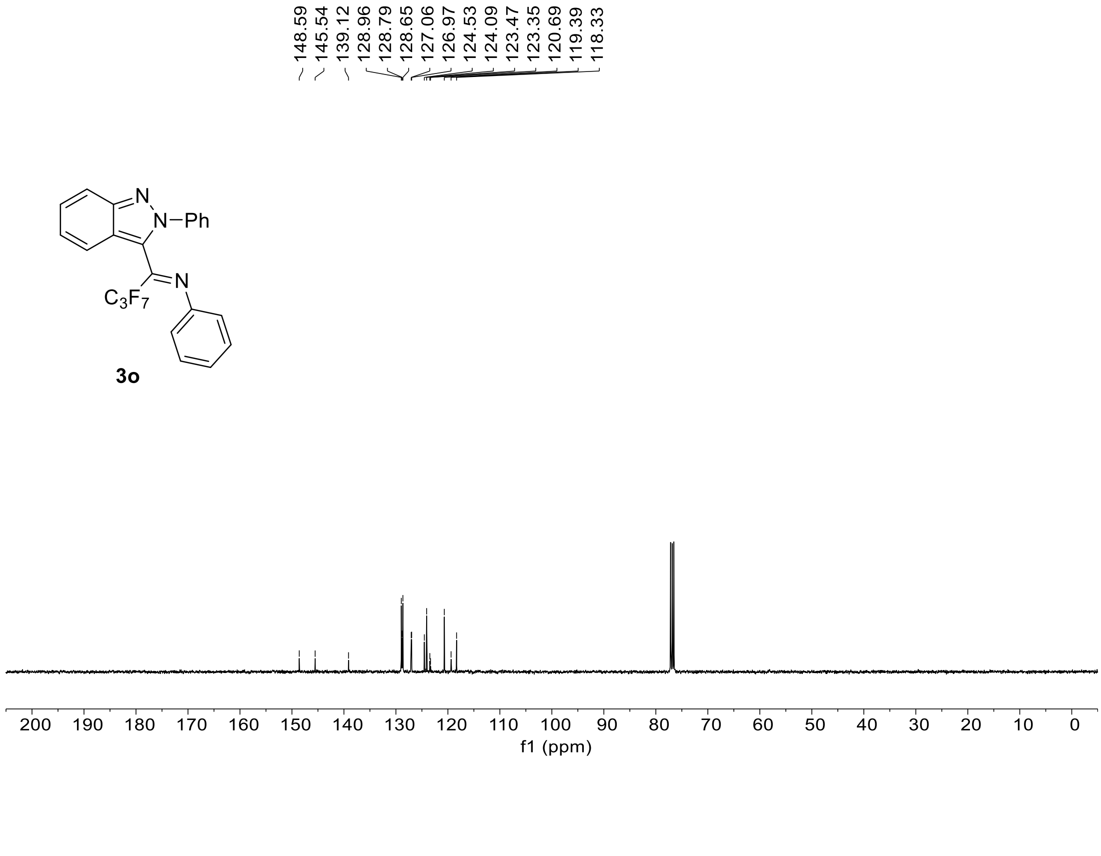

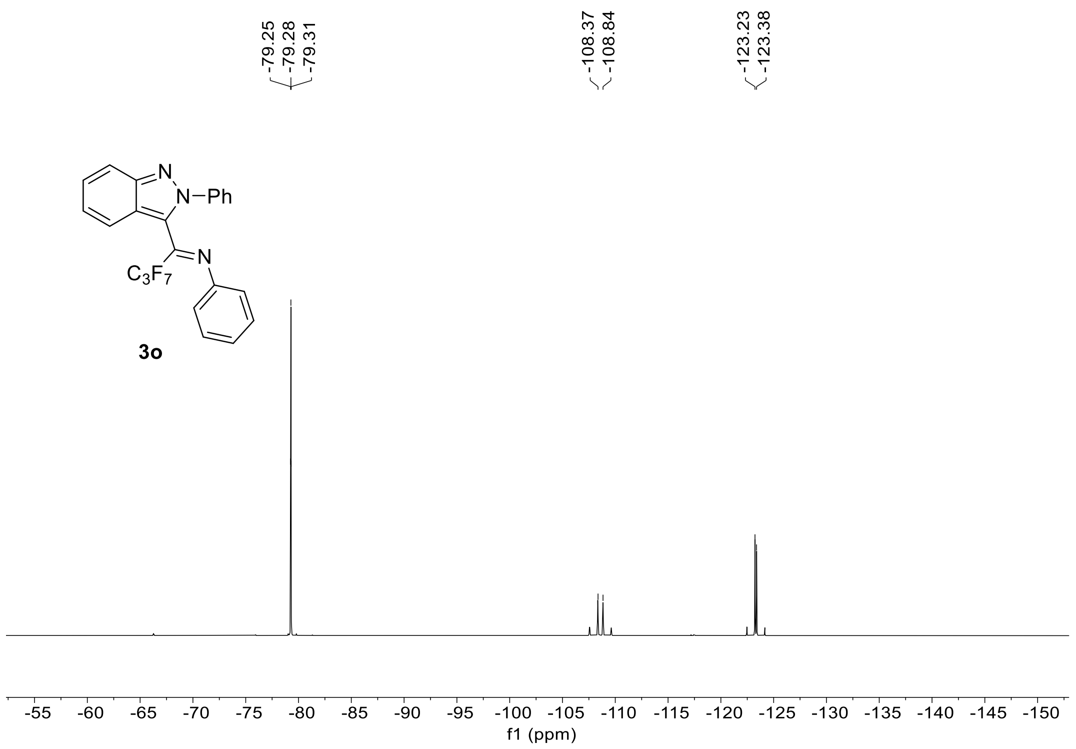

(16) ^{1}H NMR, ^{13}C NMR and ^{19}F NMR spectrum of **3p** (using $CDCl_3$ as solvent)

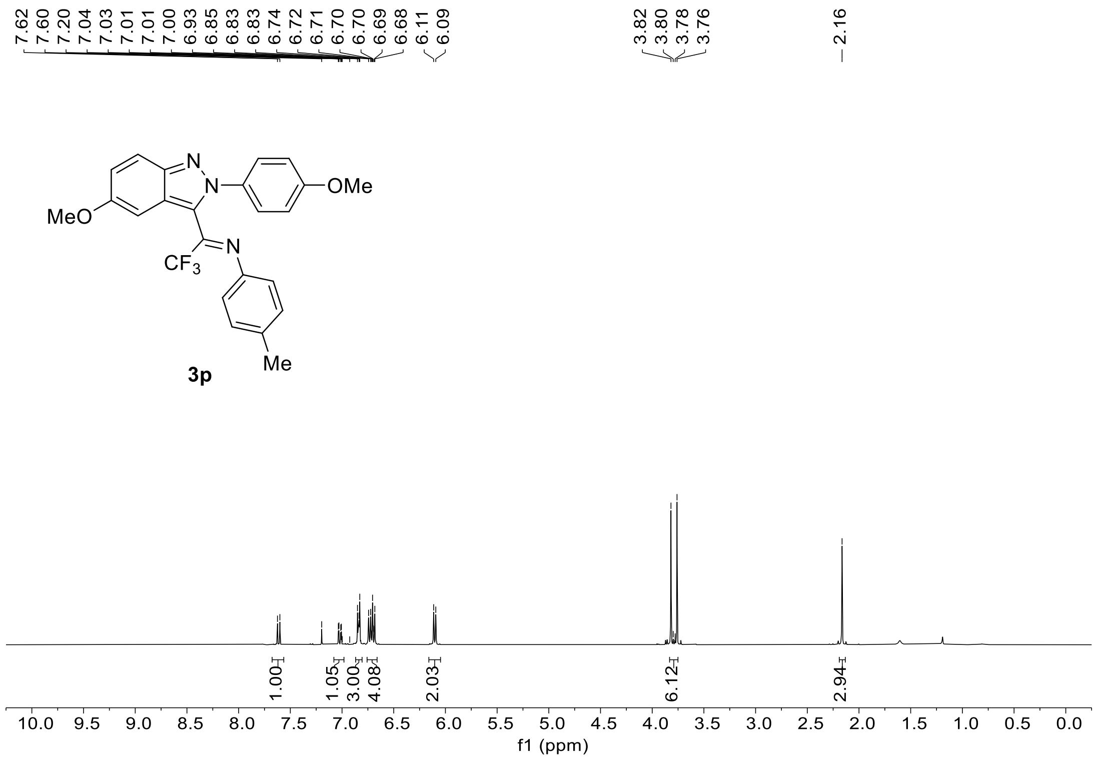

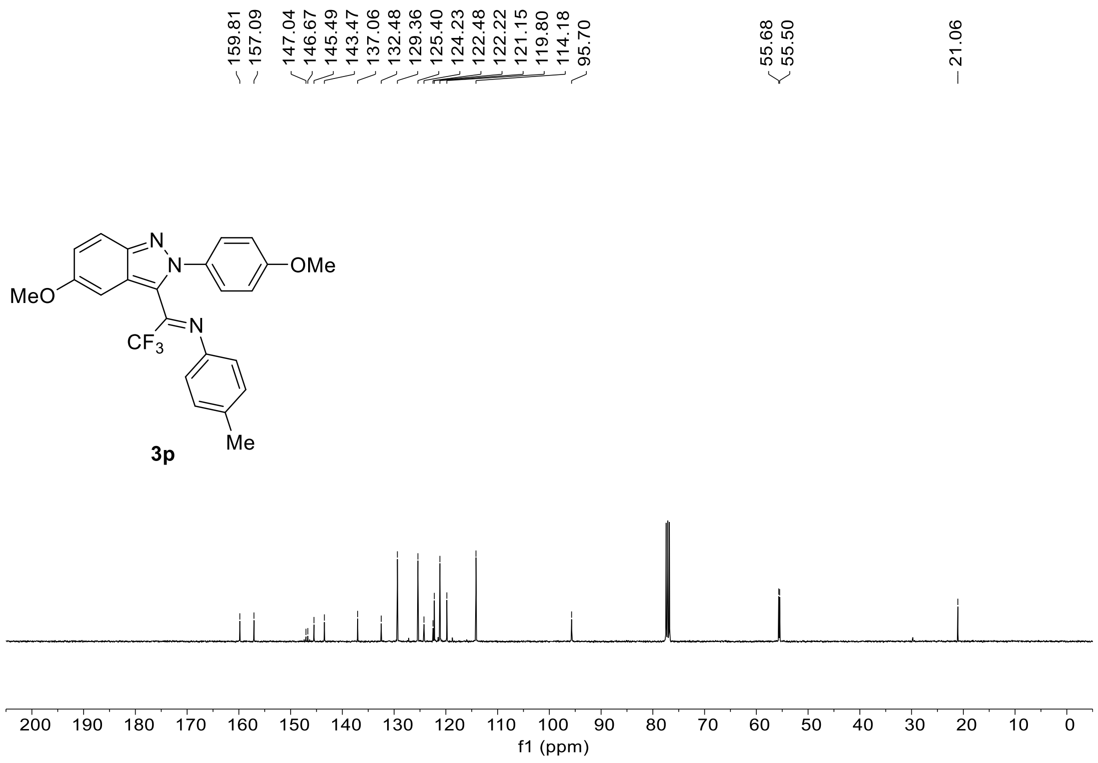
159.81
157.09
147.04
146.67
145.49
143.47
137.06
132.48
129.36
125.40
124.23
122.48
122.22
121.15
119.80
114.18
95.70
55.68
55.50
21.06
MeO
N
N
OMe
CF3
N
Me
3p
200 190 180 170 160 150 140 130 120 110 100 90 80 70 60 50 40 30 20 10 0
f1 (ppm)

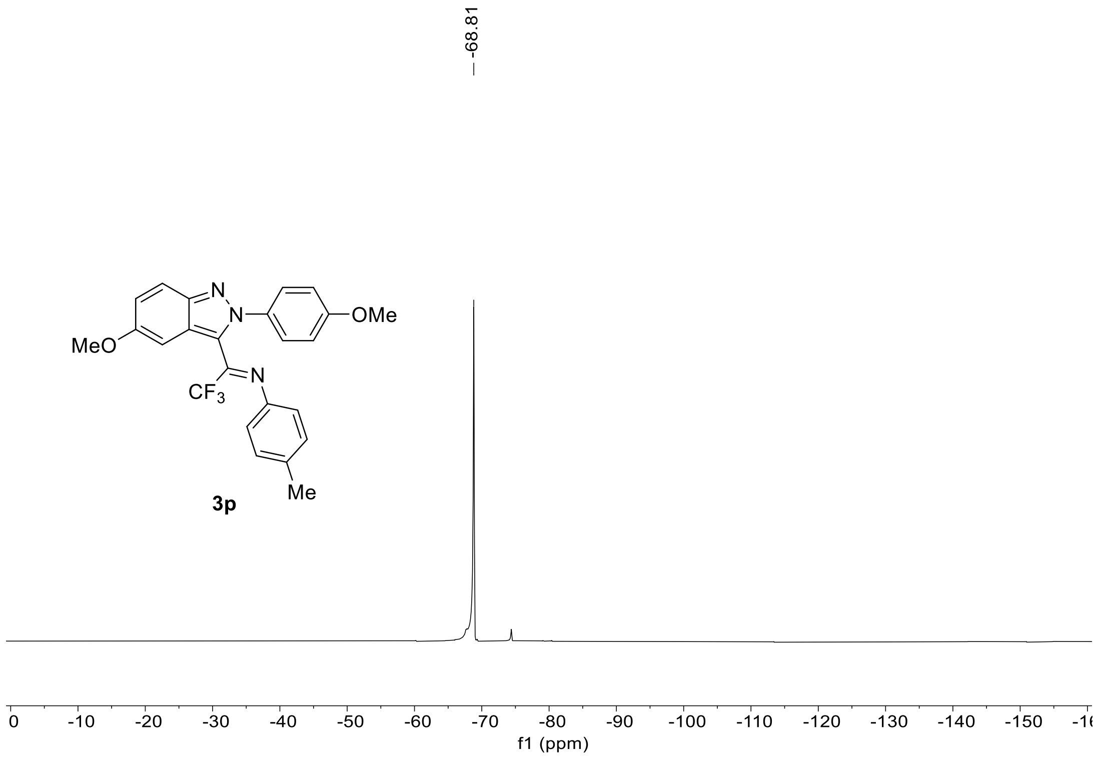
-68.81
MeO
N
N
OMe
CF3
N
Me
3p
0 -10 -20 -30 -40 -50 -60 -70 -80 -90 -100 -110 -120 -130 -140 -150
f1 (ppm)

(17) ^{1}H NMR, ^{13}C NMR and ^{19}F NMR spectrum of **3q** (using $CDCl_3$ as solvent)

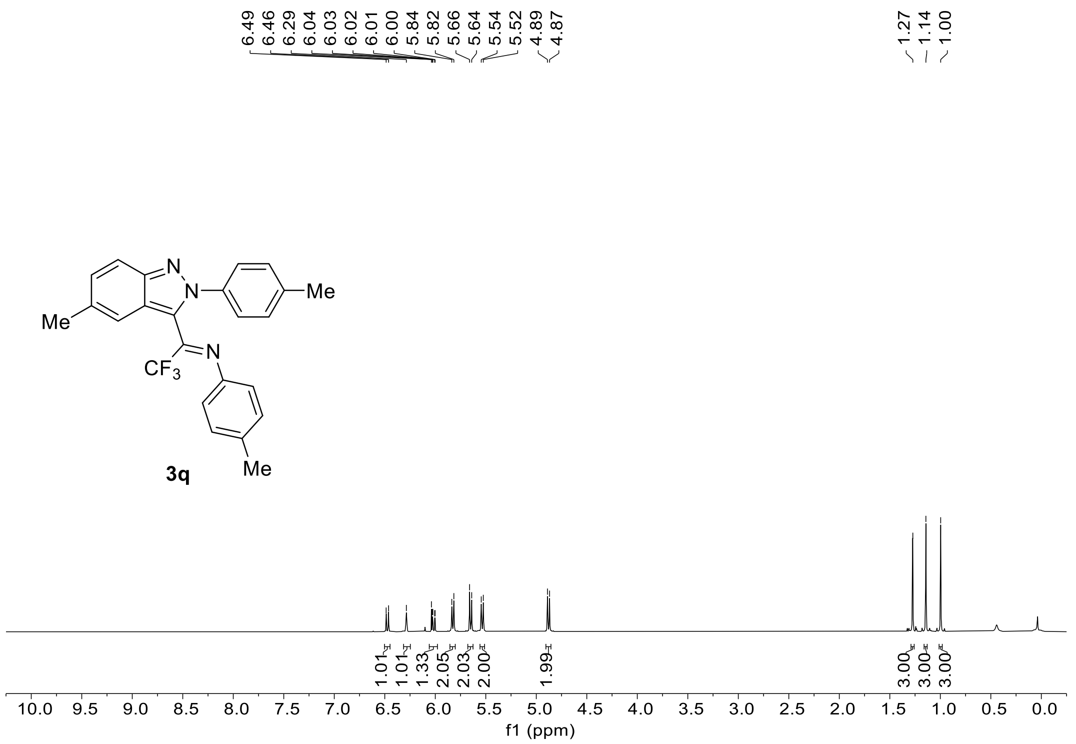

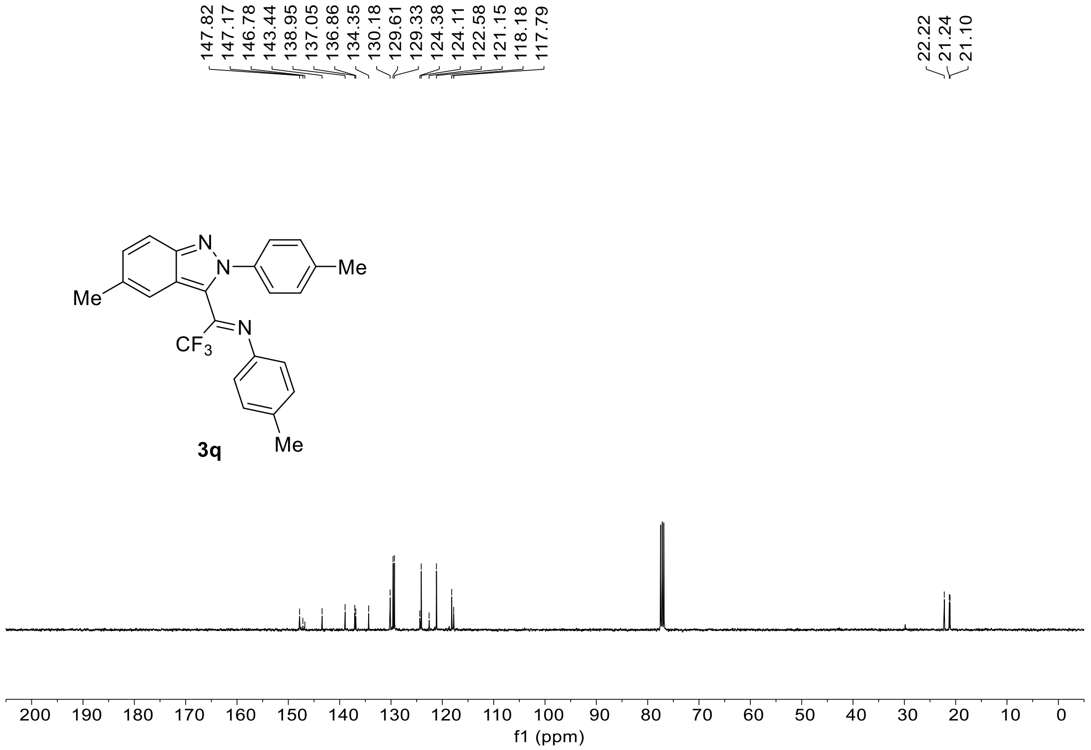

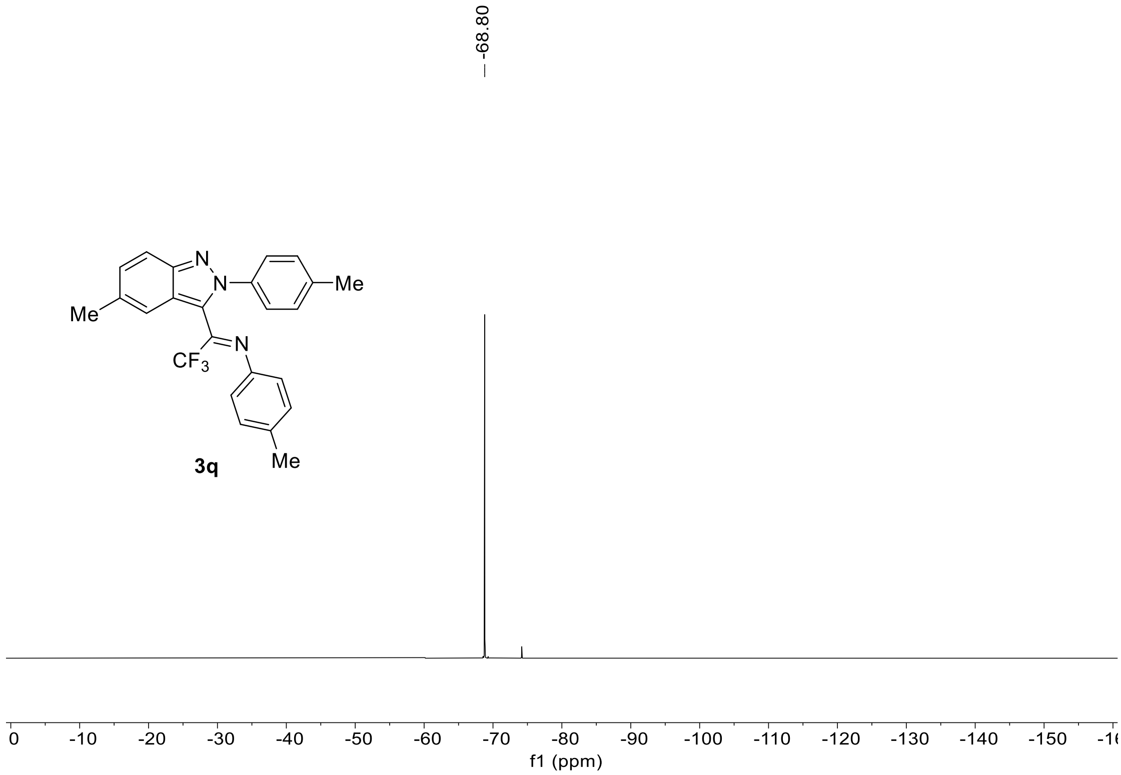

(18) ^{1}H NMR, ^{13}C NMR and ^{19}F NMR spectrum of **3r** (using $CDCl_3$ as solvent)

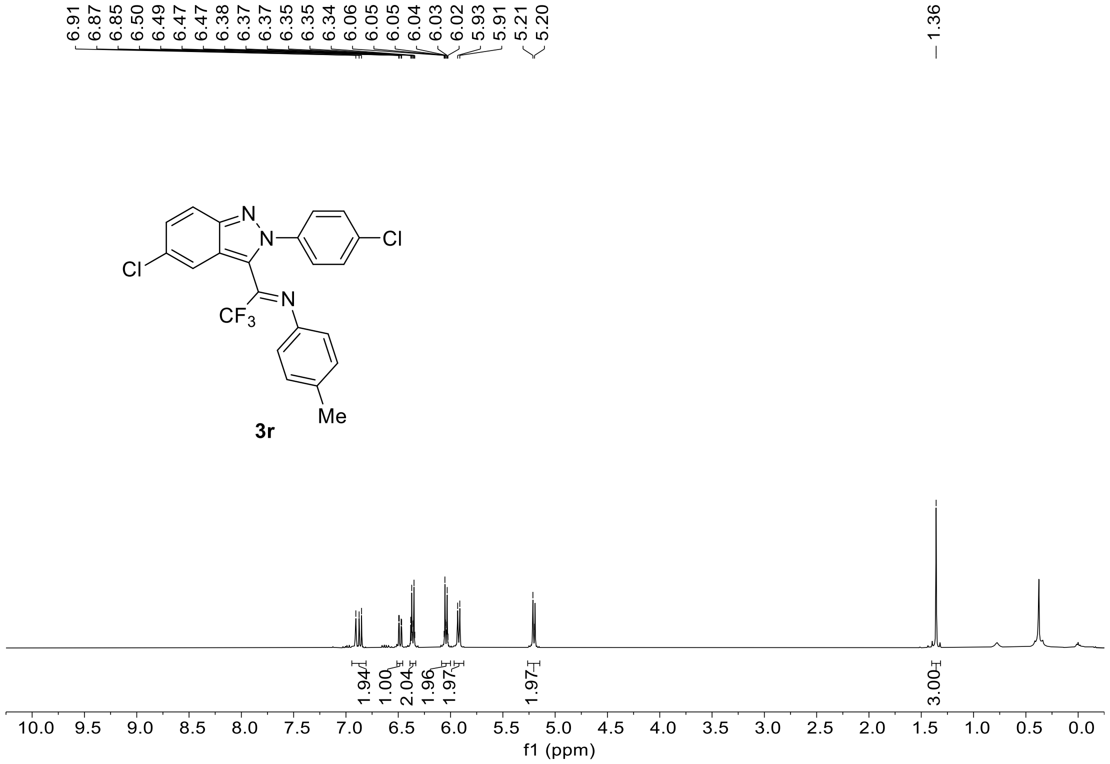

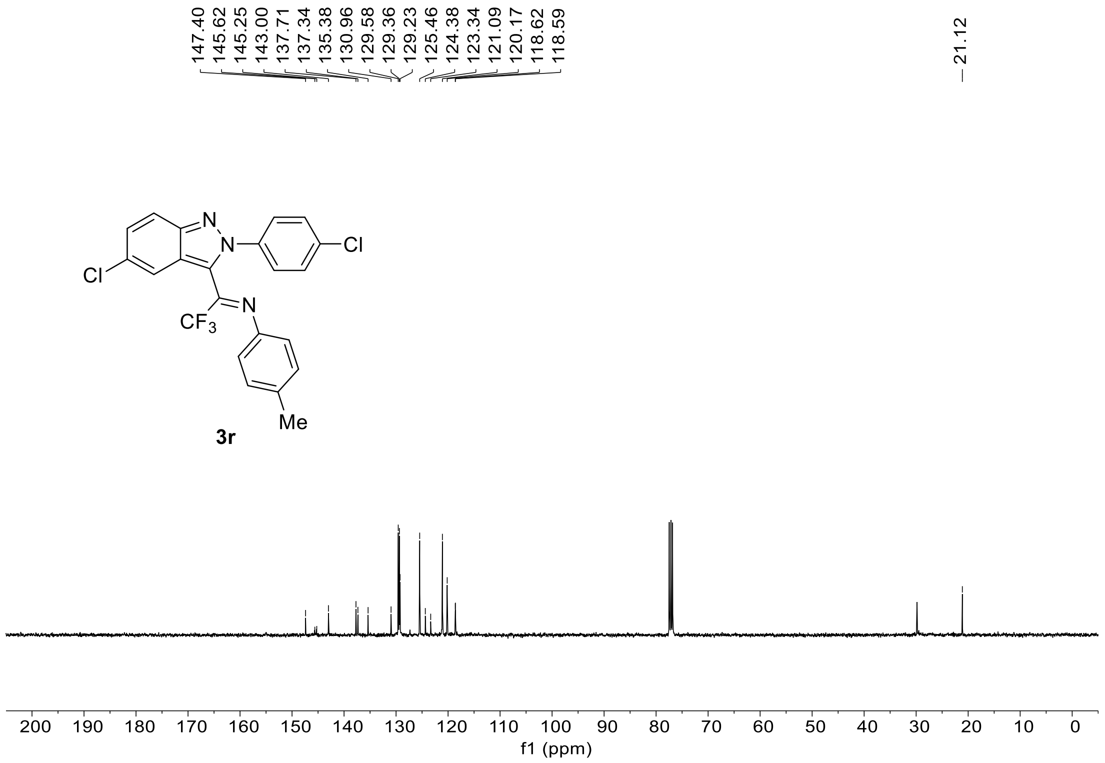
147.40
145.62
145.25
143.00
137.71
137.34
135.38
130.96
129.58
129.36
129.23
125.46
124.38
123.34
121.09
120.17
118.62
118.59
21.12
N
N
Cl
Cl
CF3
N
Me
3r
200
190
180
170
160
150
140
130
120
110
100
90
80
70
60
50
40
30
20
10
0
f1 (ppm)

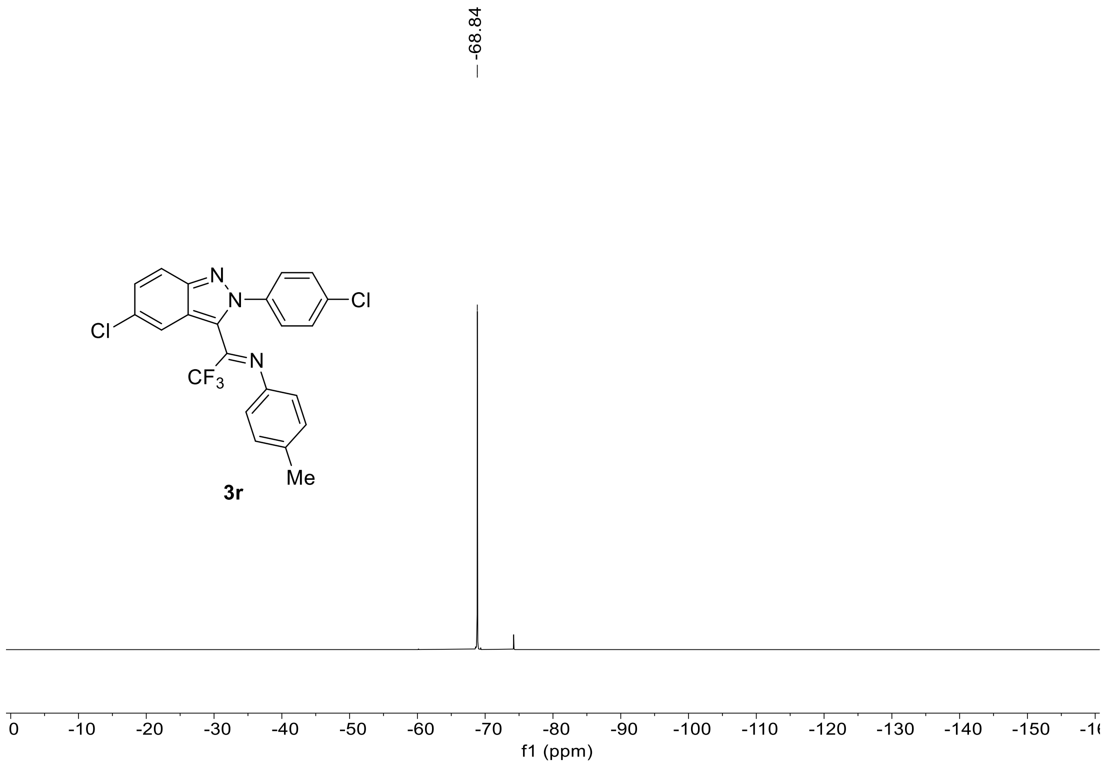
-68.84
N
N
Cl
Cl
CF3
N
Me
3r
0
-10
-20
-30
-40
-50
-60
-70
-80
-90
-100
-110
-120
-130
-140
-150
f1 (ppm)

(19) ^{1}H NMR, ^{13}C NMR and ^{19}F NMR spectrum of **3s** (using $CDCl_3$ as solvent)

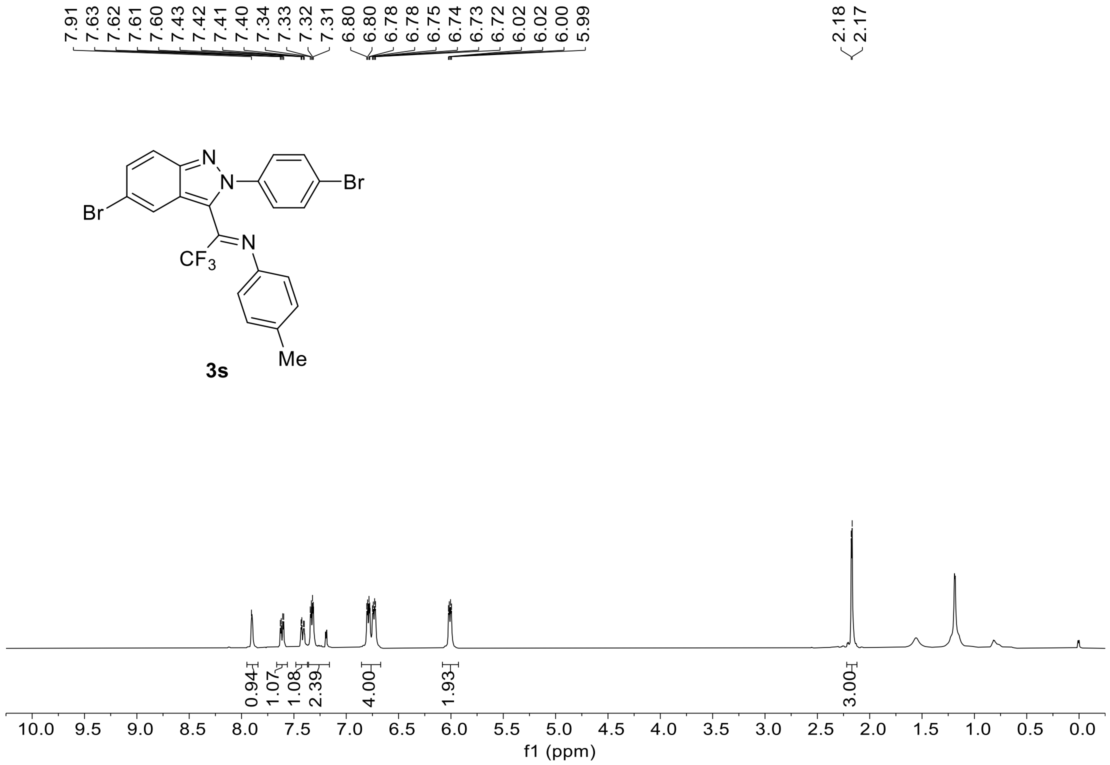

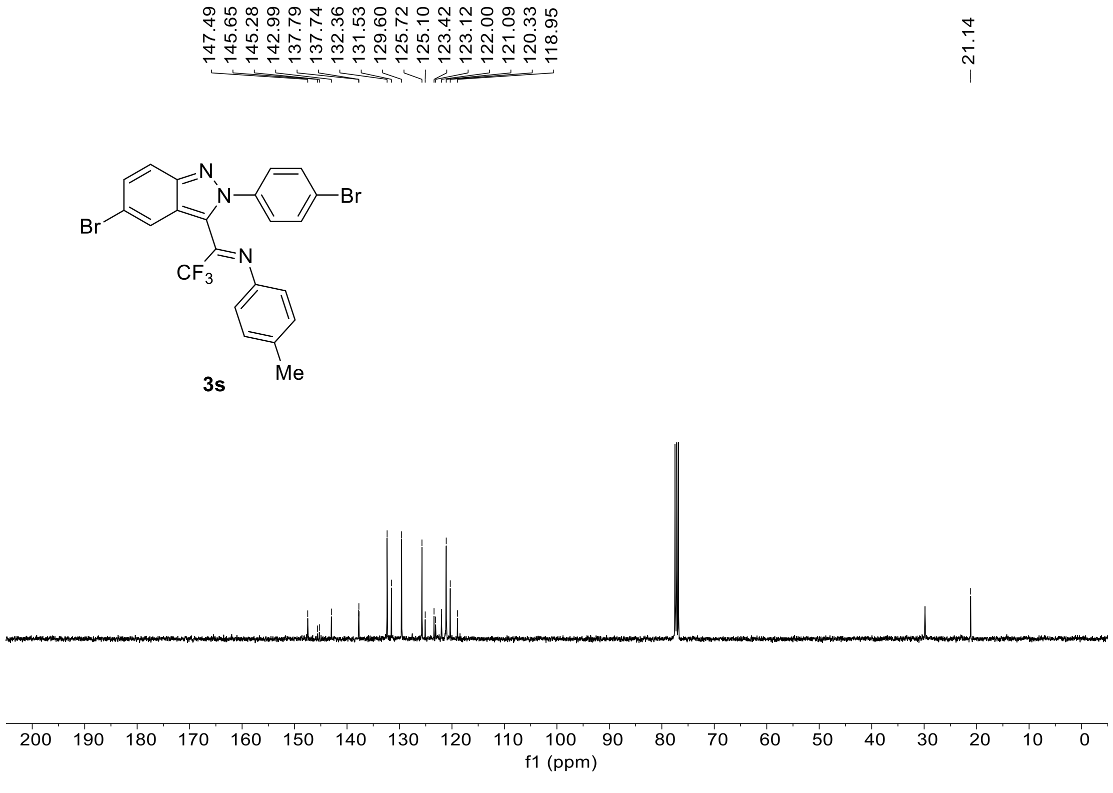

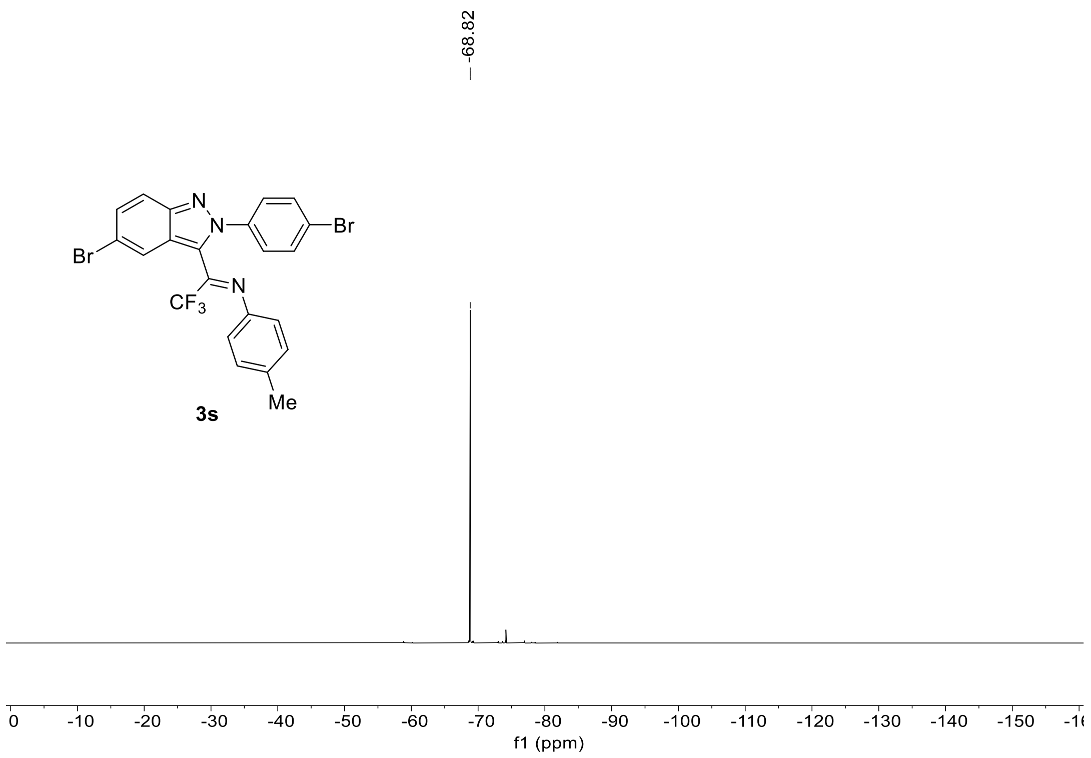

(20) ^{1}H NMR, ^{13}C NMR and ^{19}F NMR spectrum of **3t** (using $CDCl_3$ as solvent)

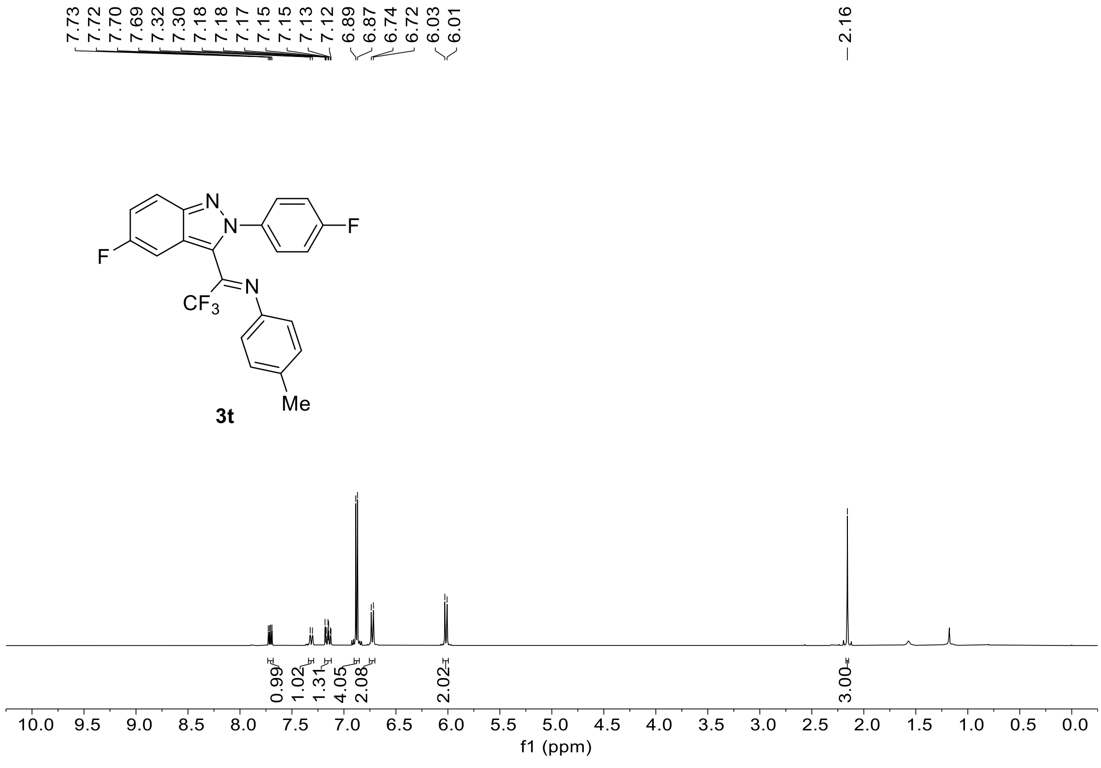

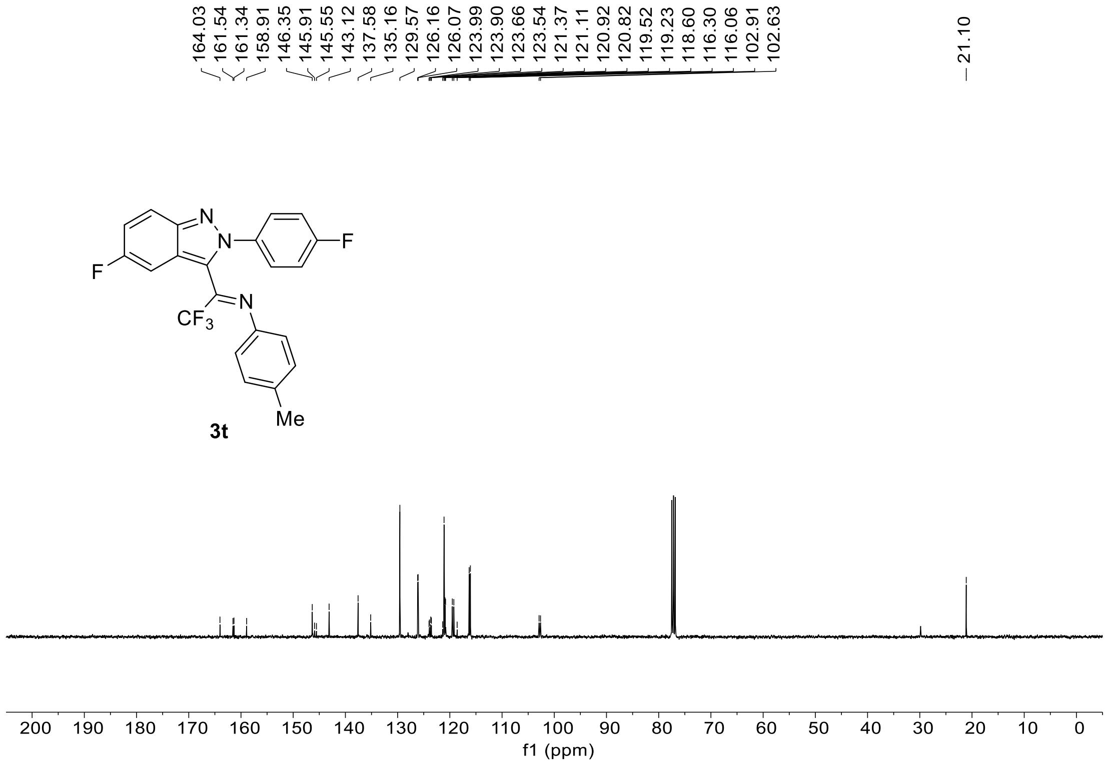
164.03
161.54
161.34
158.91
146.35
145.91
145.55
143.12
137.58
135.16
129.57
126.16
126.07
123.99
123.90
123.66
123.54
121.37
121.11
120.92
120.82
119.52
119.23
118.60
116.30
116.06
102.91
102.63
21.10
3t
f1 (ppm)

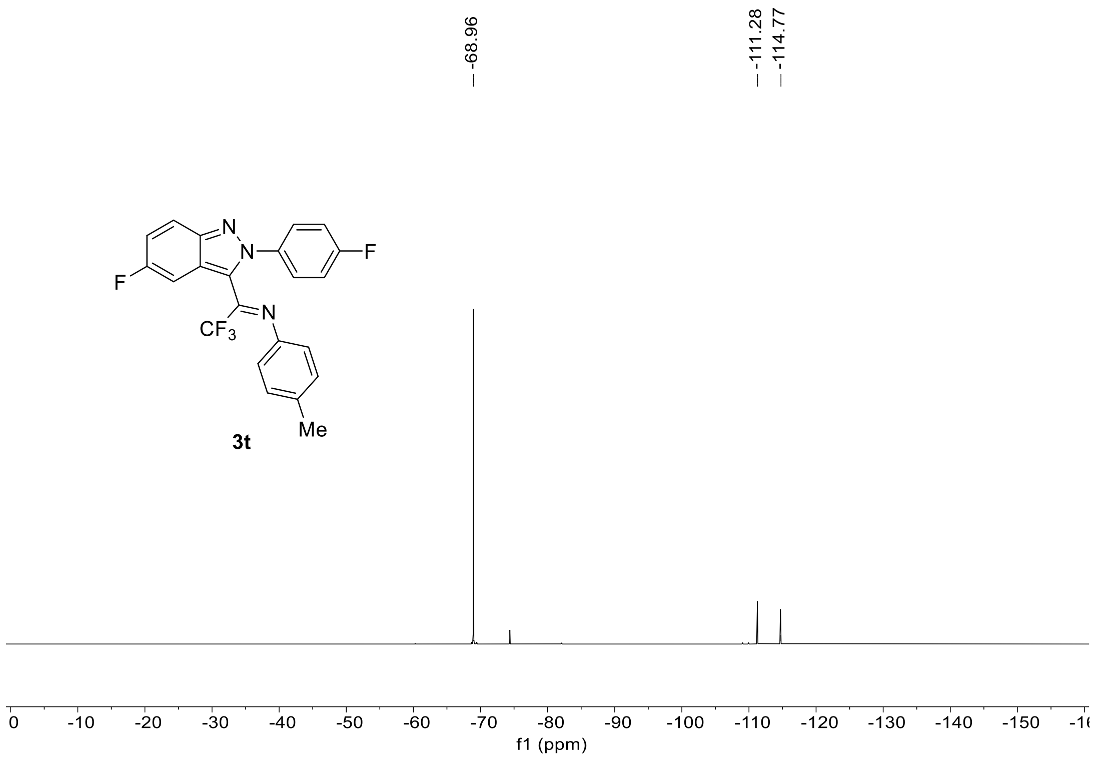
-68.96
-111.28
-114.77
3t
f1 (ppm)

(21) ^{1}H NMR, ^{13}C NMR and ^{19}F NMR spectrum of **3u** (using $CDCl_3$ as solvent)

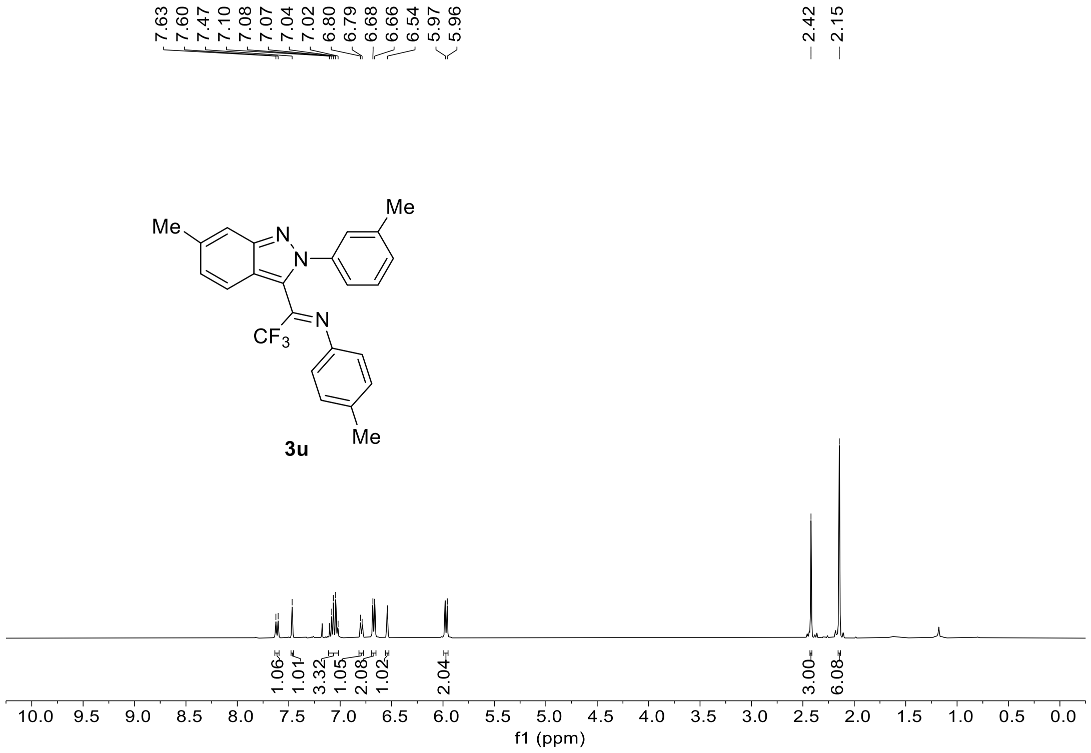

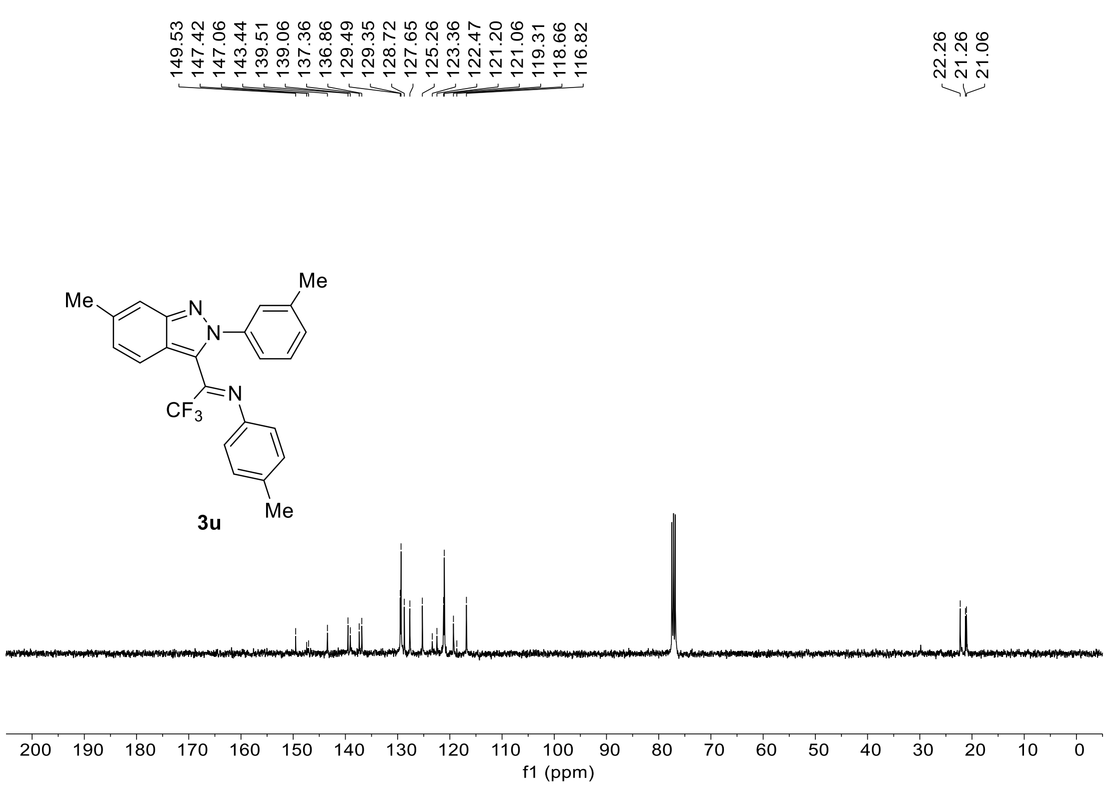

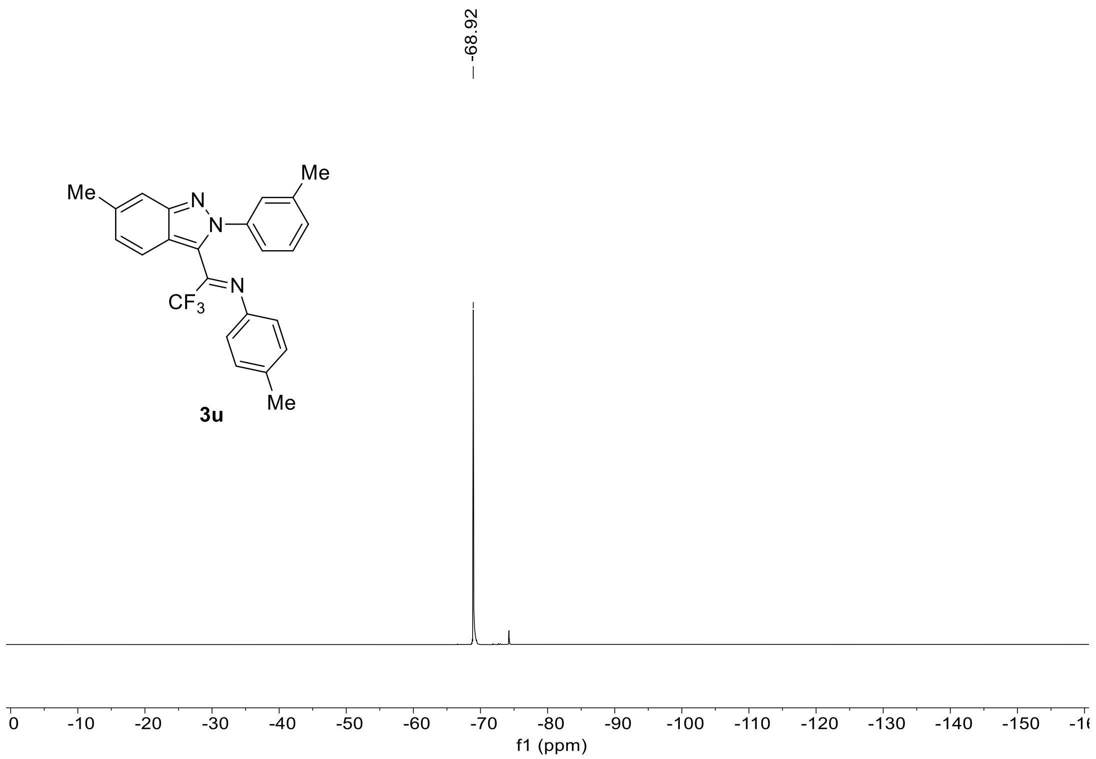

(22) ^{1}H NMR, ^{13}C NMR and ^{19}F NMR spectrum of **3v** (using $CDCl_3$ as solvent)

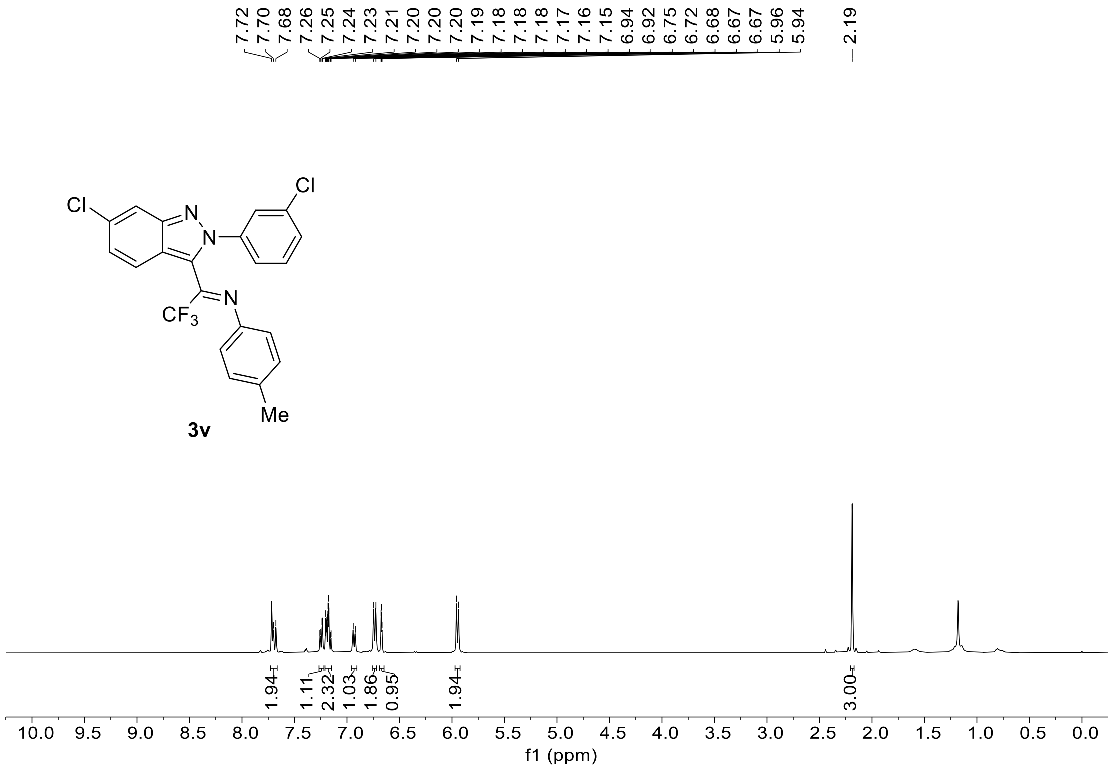

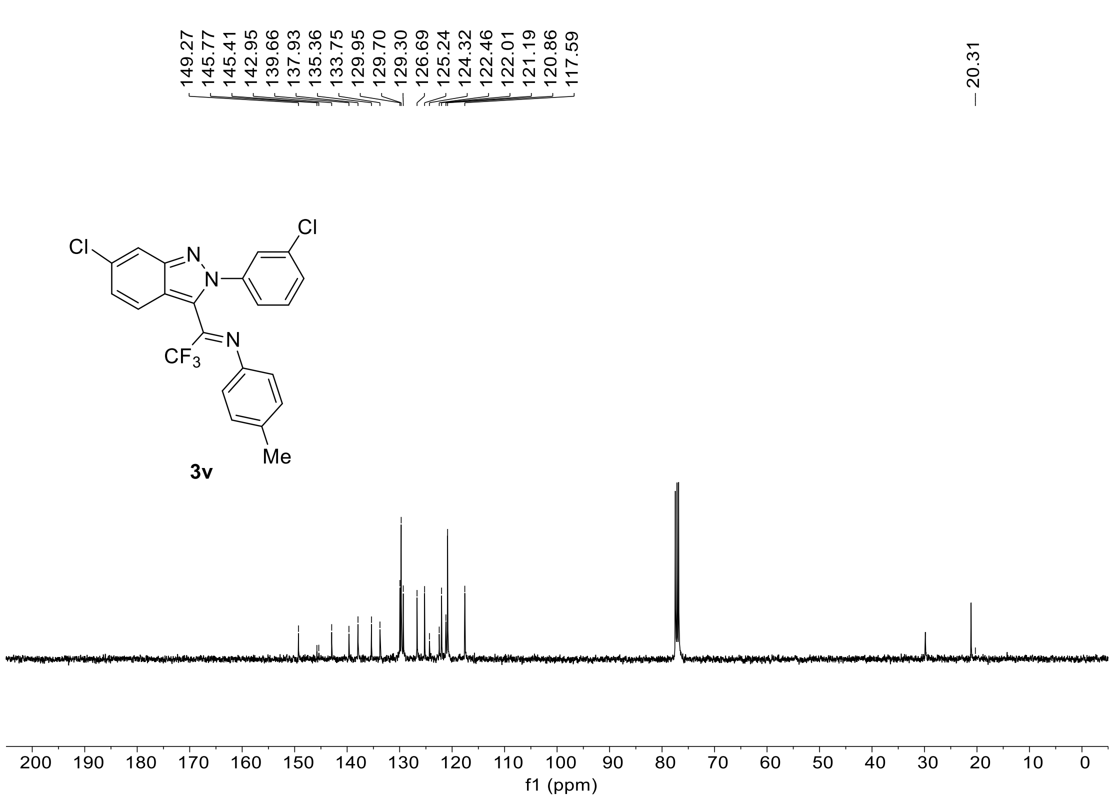

3v

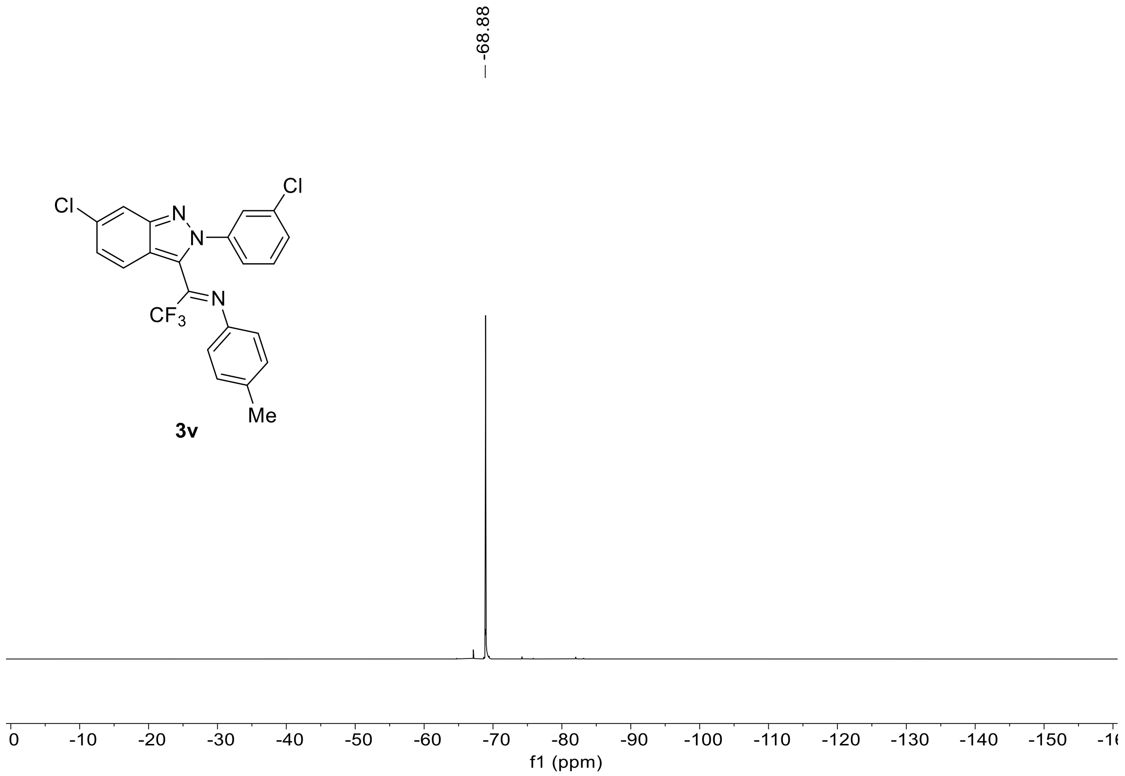

3v

(23) ^{1}H NMR, ^{13}C NMR and ^{19}F NMR spectrum of **3w** (using $CDCl_3$ as solvent)

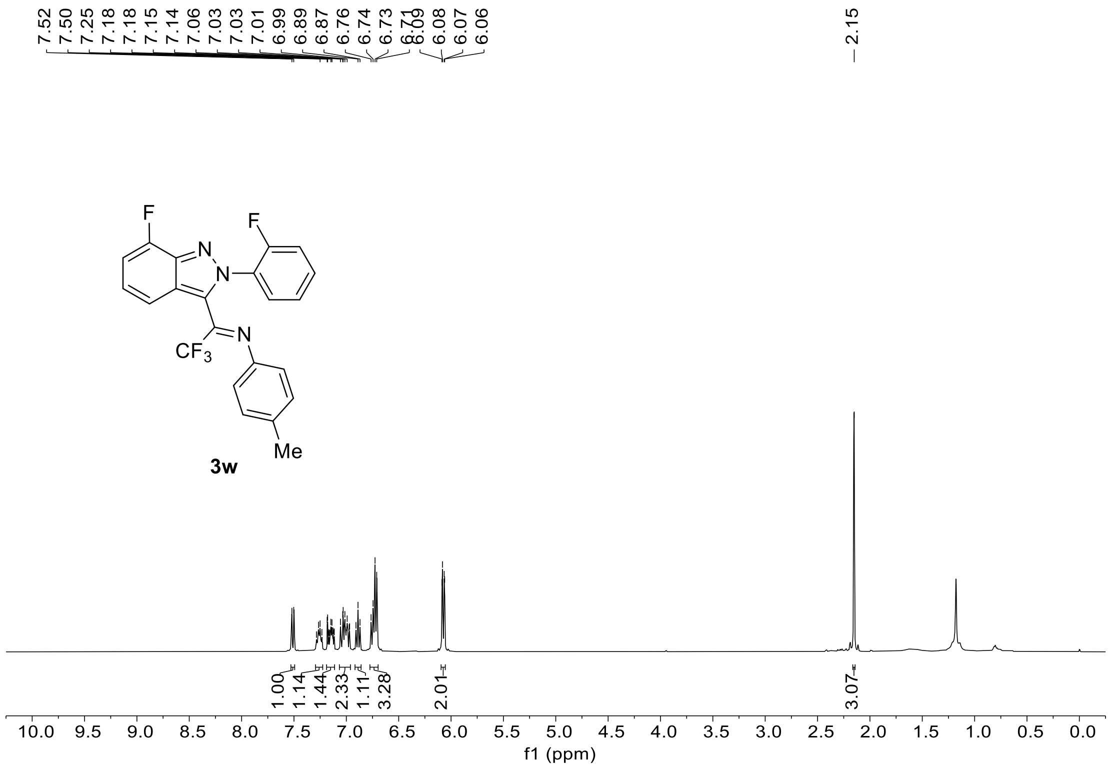

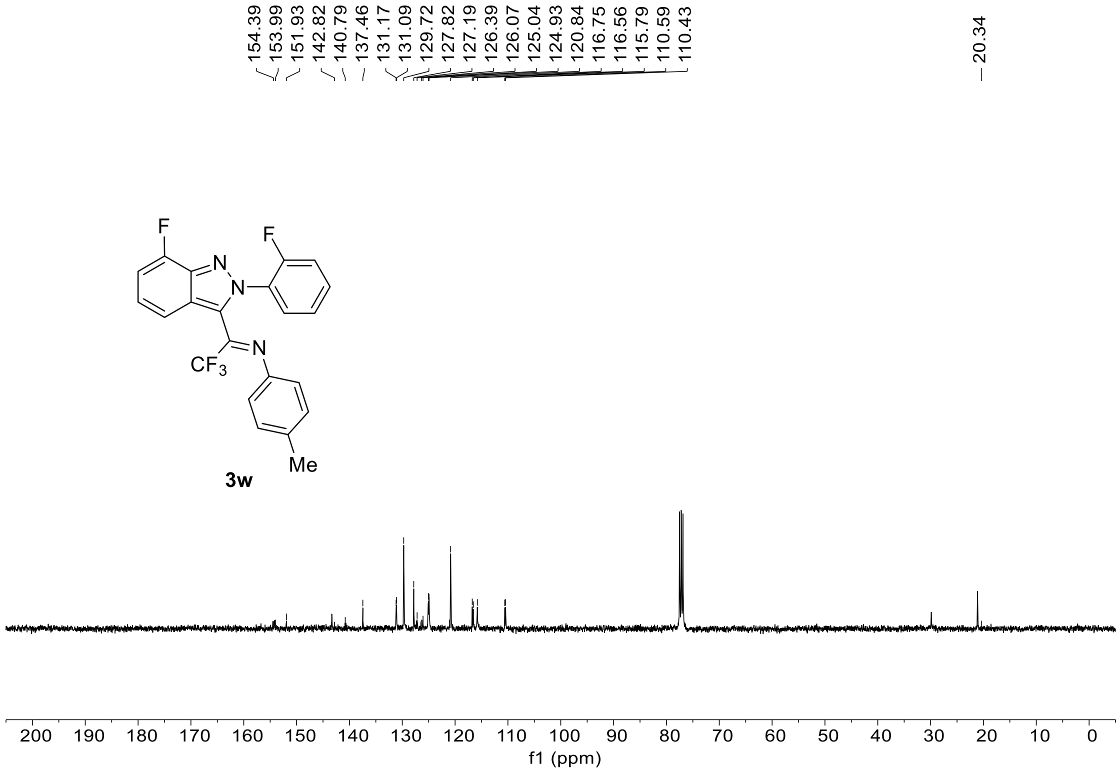

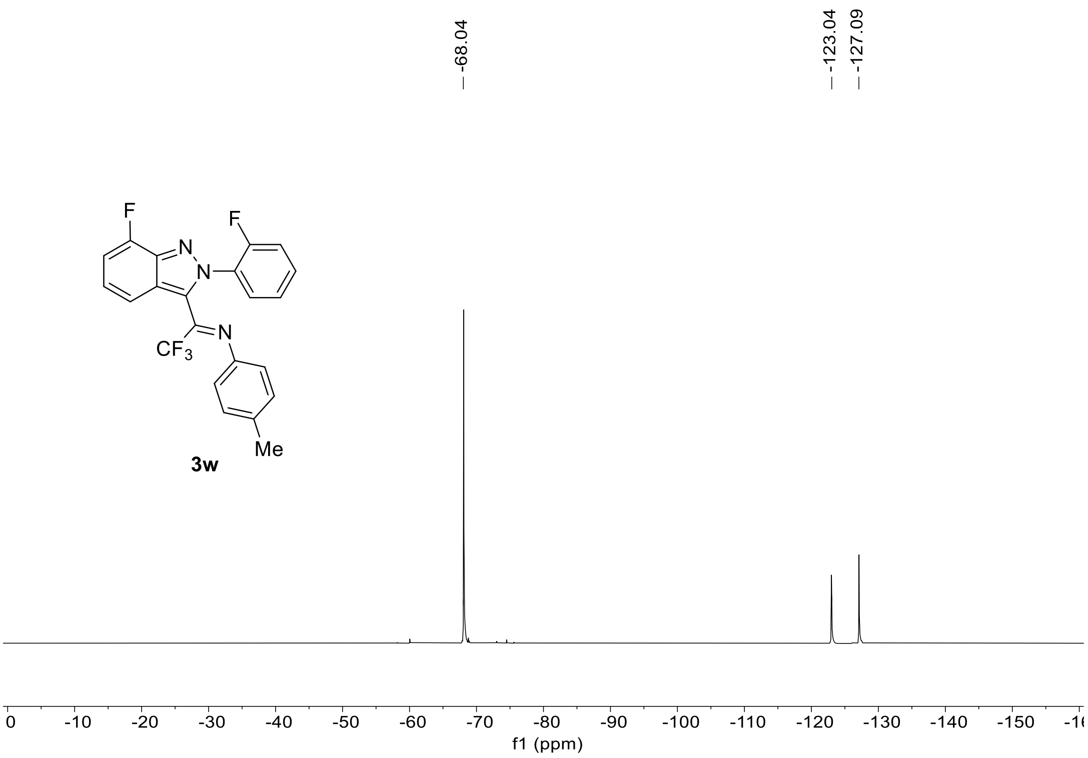

(24) ^{1}H NMR, ^{13}C NMR and ^{19}F NMR spectrum of **3x** (using $CDCl_3$ as solvent)

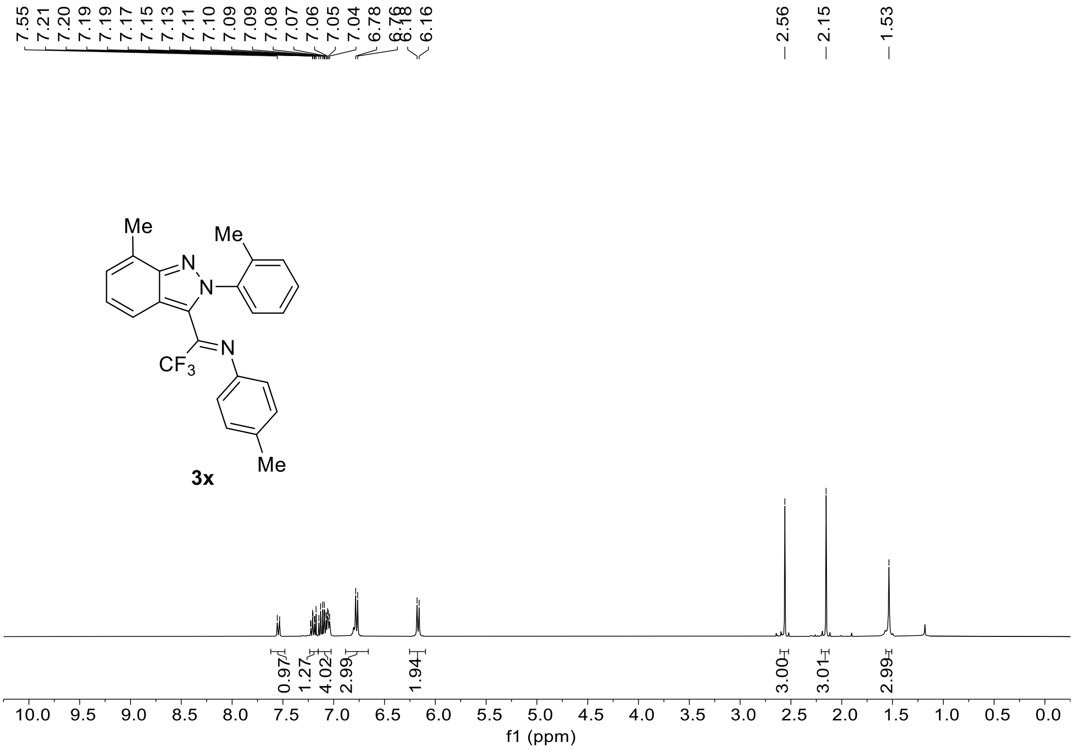

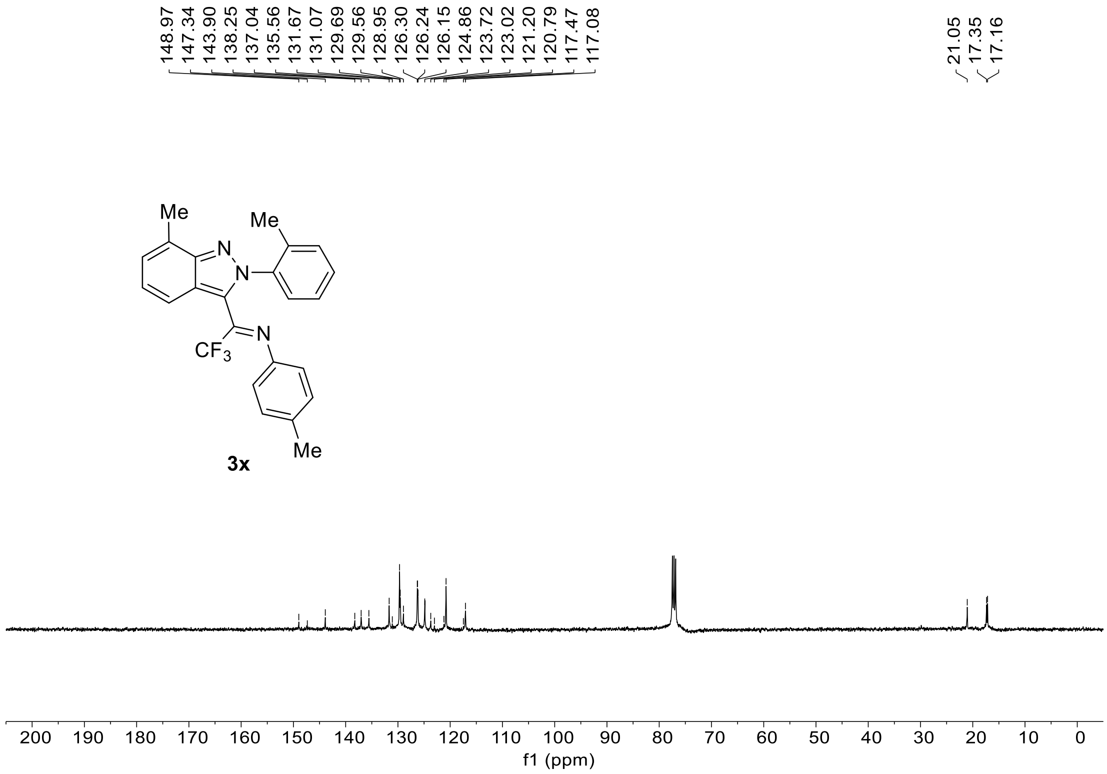
148.97
147.34
143.90
138.25
137.04
135.56
131.67
131.07
129.69
129.56
128.95
126.30
126.24
126.15
124.86
123.72
123.02
121.20
120.79
117.47
117.08
21.05
17.35
17.16
Me
Me
N
N
CF3
N
Me
3x
200 190 180 170 160 150 140 130 120 110 100 90 80 70 60 50 40 30 20 10 0
f1 (ppm)

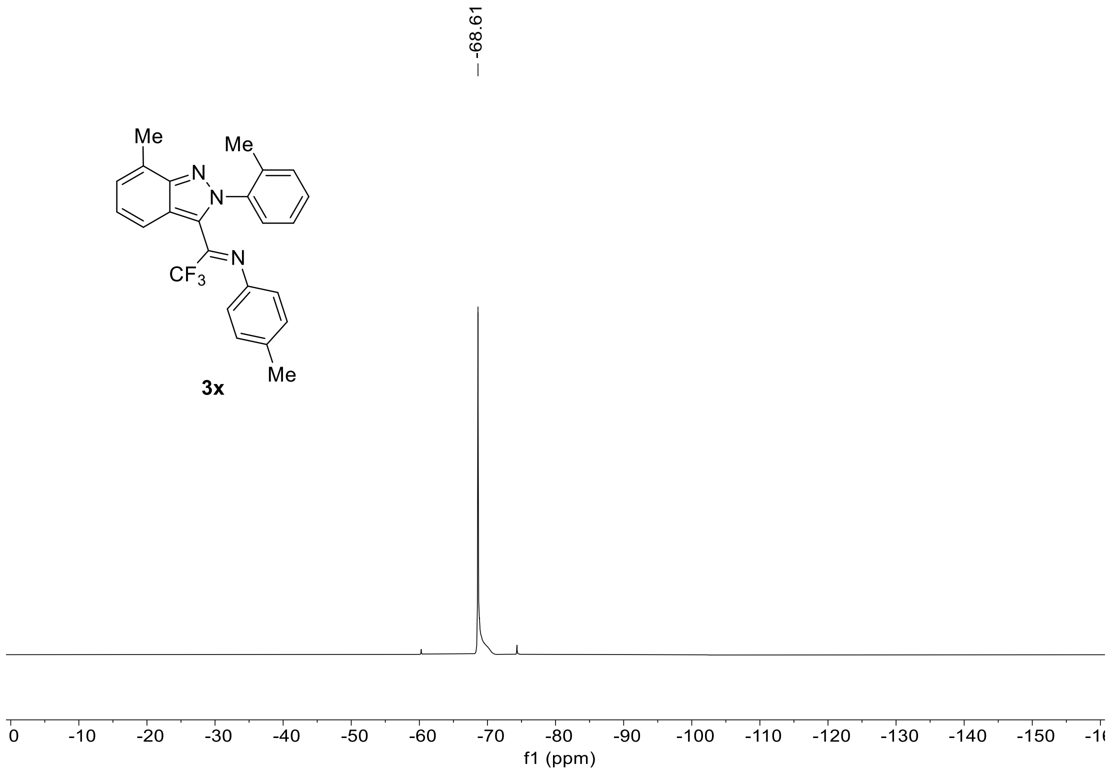
-68.61
Me
Me
N
N
CF3
N
Me
3x
0 -10 -20 -30 -40 -50 -60 -70 -80 -90 -100 -110 -120 -130 -140 -150
f1 (ppm)

(25) ^{1}H NMR and ^{13}C NMR of **Complex I** (using $CDCl_3$ as solvent)

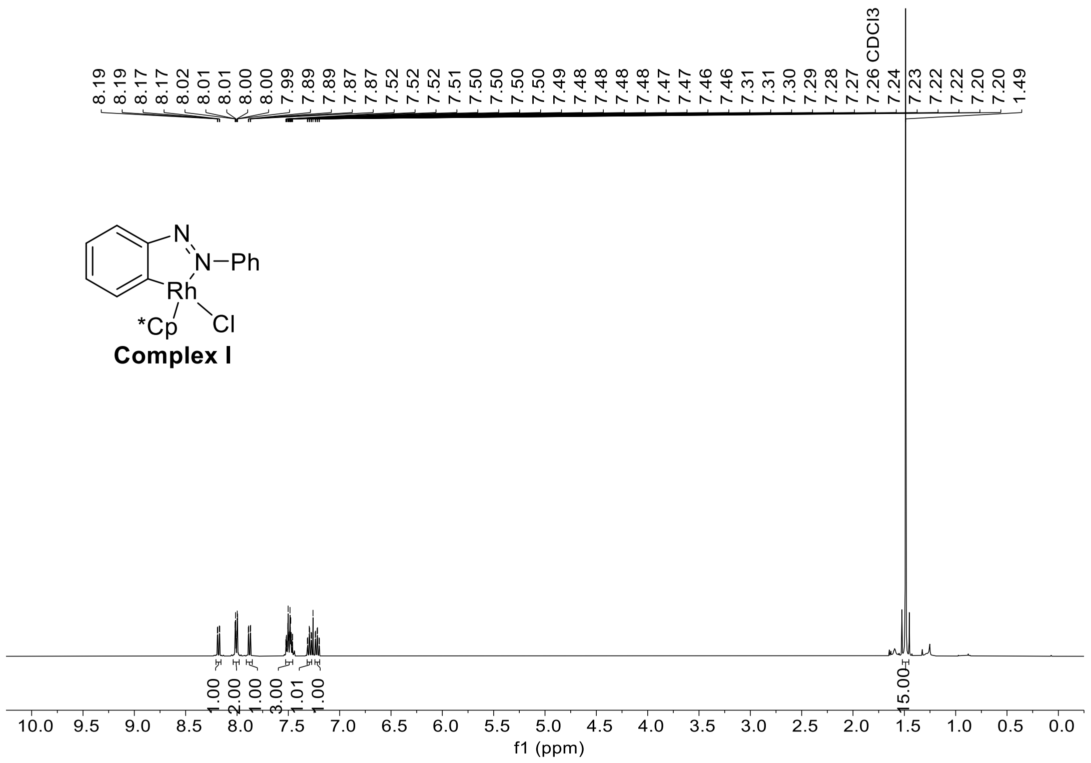

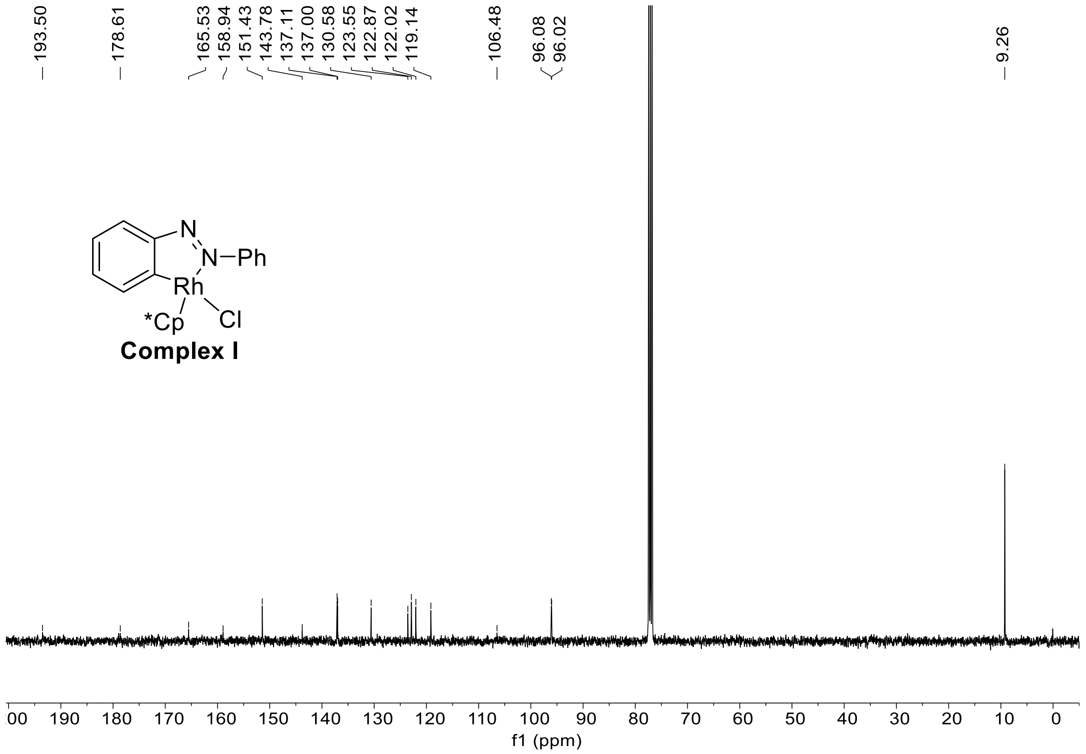

6. HRMS spectrum for all unknown compounds

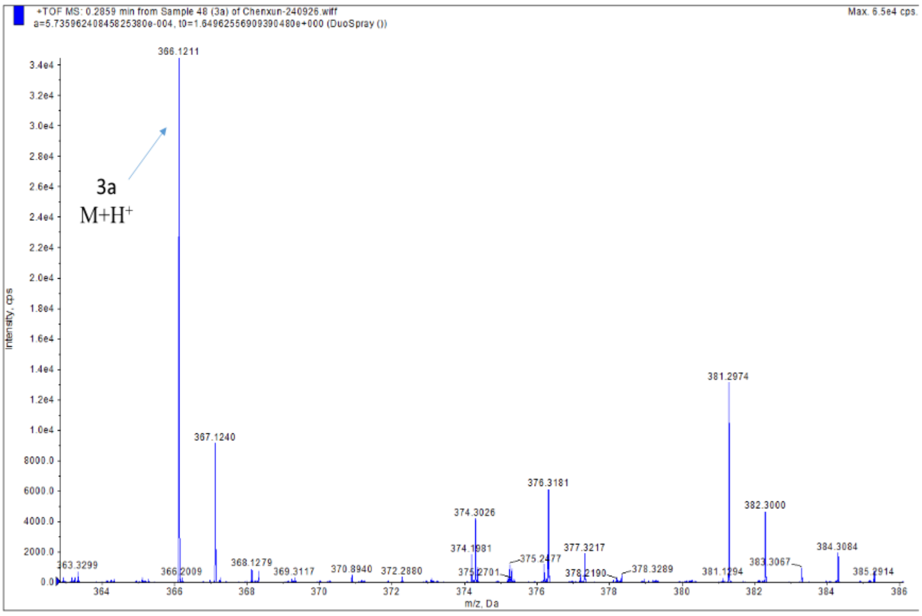

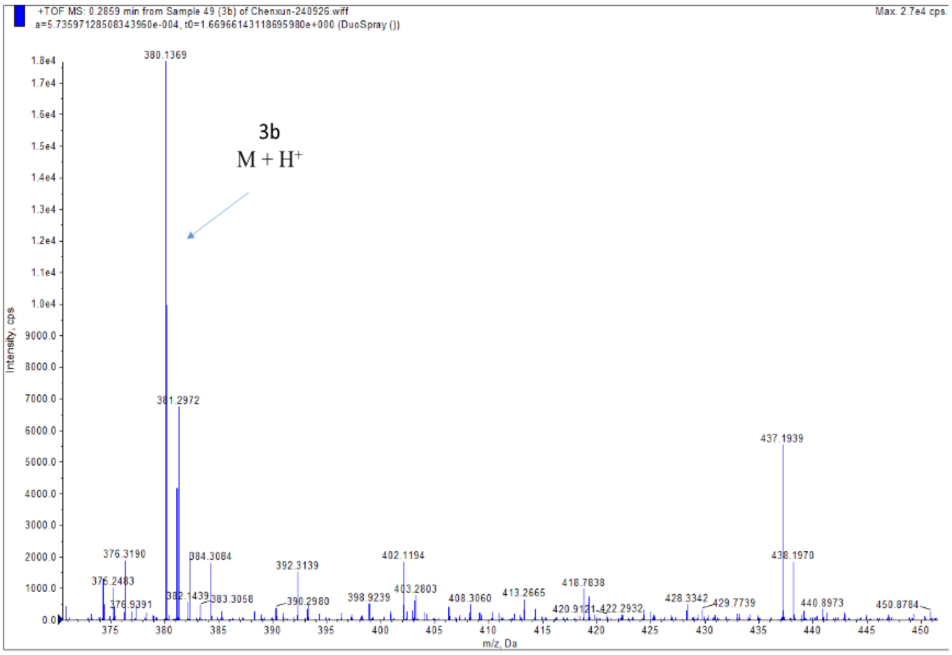

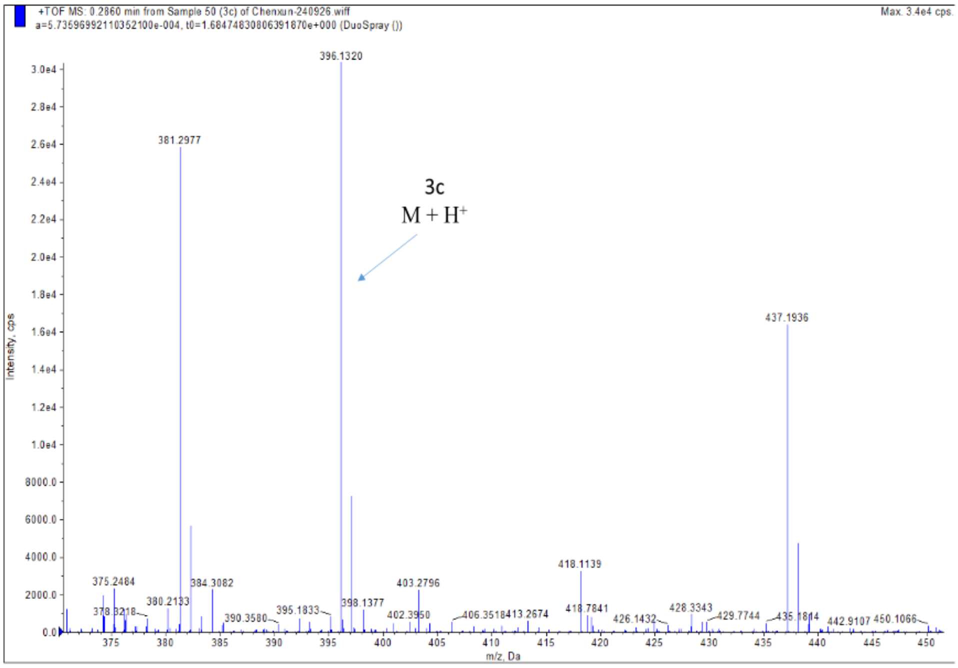
+TOF MS: 0.2860 min from Sample 50 (3c) of Chenxun-240926.wiff
Max. 3.4e4 cps.
3c
M + H+
396.1320
381.2977
437.1936
418.1139
403.2796
384.3082
375.2484
Intensity, cps
m/z, Da

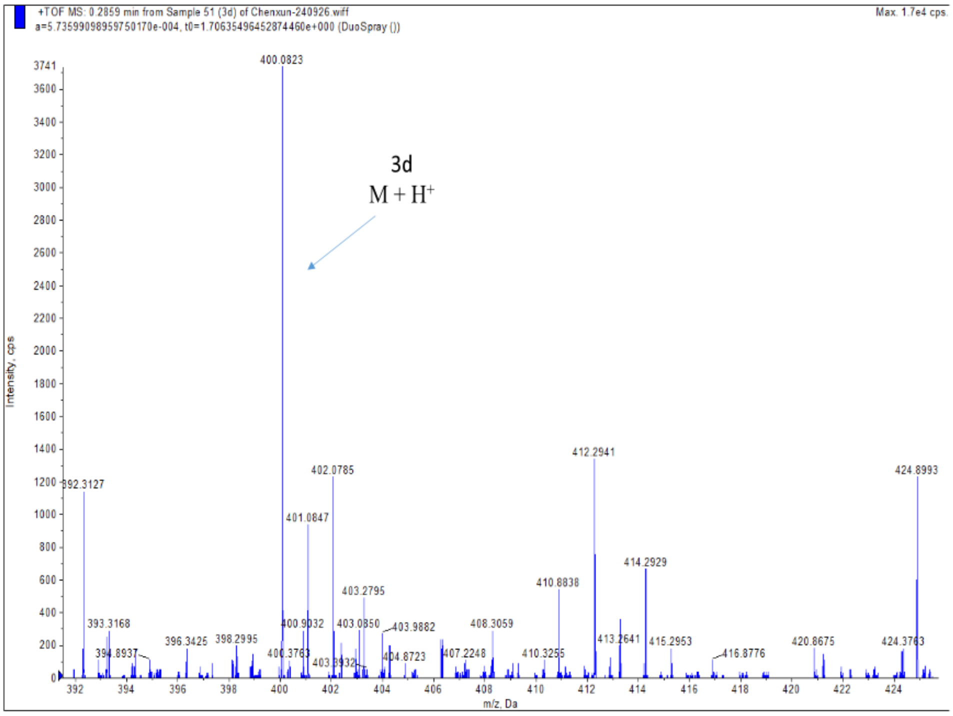
+TOF MS: 0.2859 min from Sample 51 (3d) of Chenxun-240926.wiff
Max. 1.7e4 cps.
3d
M + H+
400.0823
412.2941
424.8993
402.0785
392.3127
401.0847
414.2929
410.8838
403.2795
Intensity, cps
m/z, Da

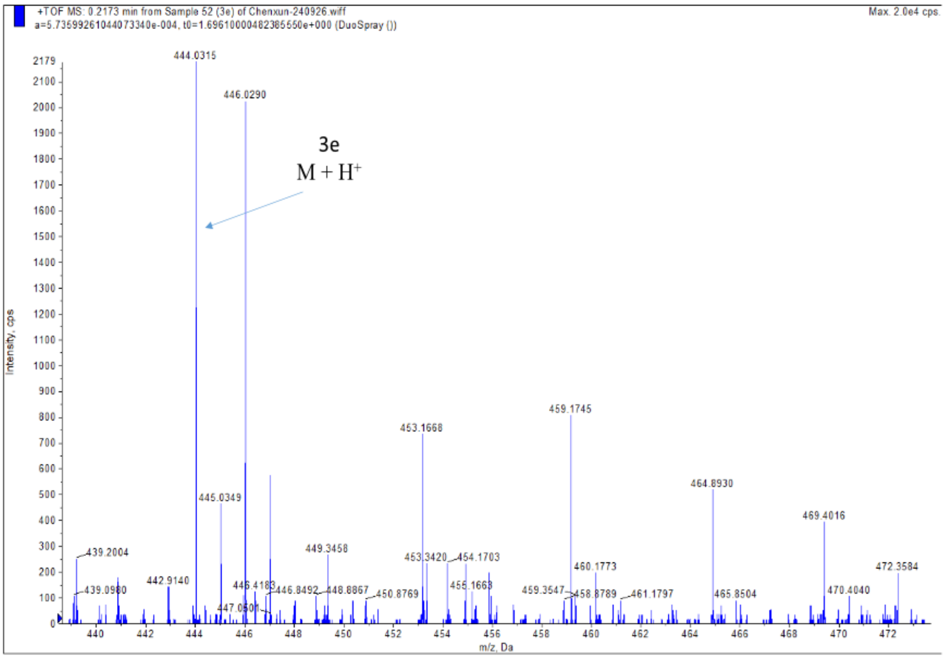

+TOF MS: 0.2173 min from Sample 52 (3e) of Chenxun-240926.wiff
a=5.73599261044073340e-004, t0=1.69610000482385550e+000 (DuoSpray ())
Max. 2.0e4 cps.
Intensity, cps
m/z, Da
444.0315
446.0290
3e
M + H+
453.1668
459.1745
464.8930
469.4016

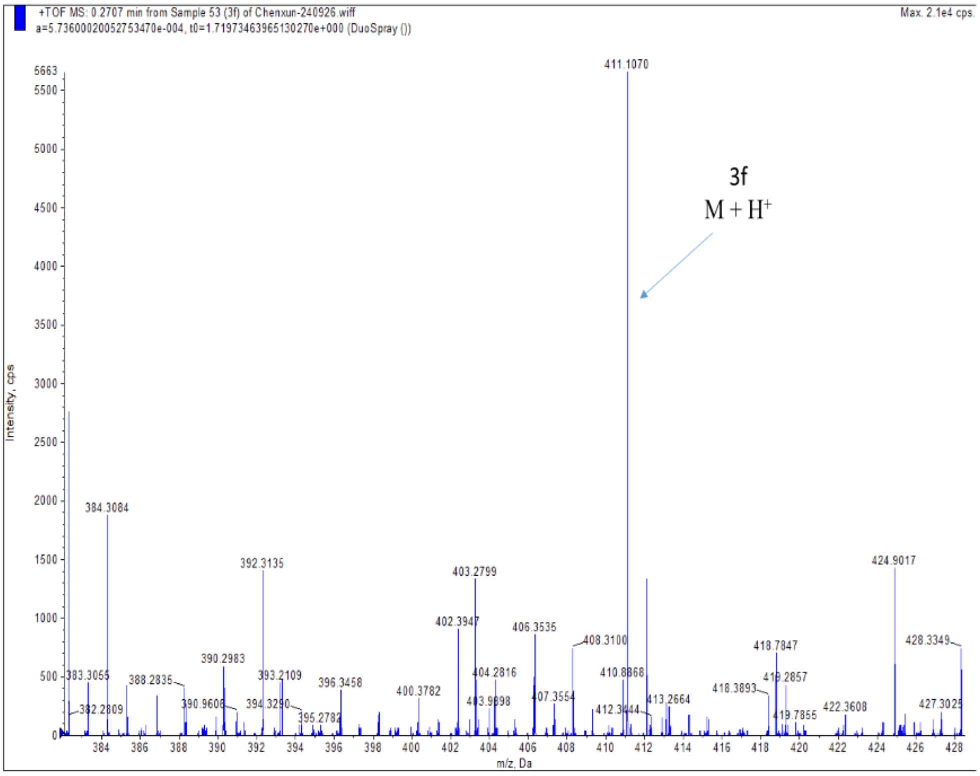

+TOF MS: 0.2707 min from Sample 53 (3f) of Chenxun-240926.wiff
a=5.73600020052753470e-004, t0=1.71973463965130270e+000 (DuoSpray ())
Max. 2.1e4 cps.
Intensity, cps
m/z, Da
411.1070
3f
M + H+
384.3084
392.3135
403.2799
424.9017

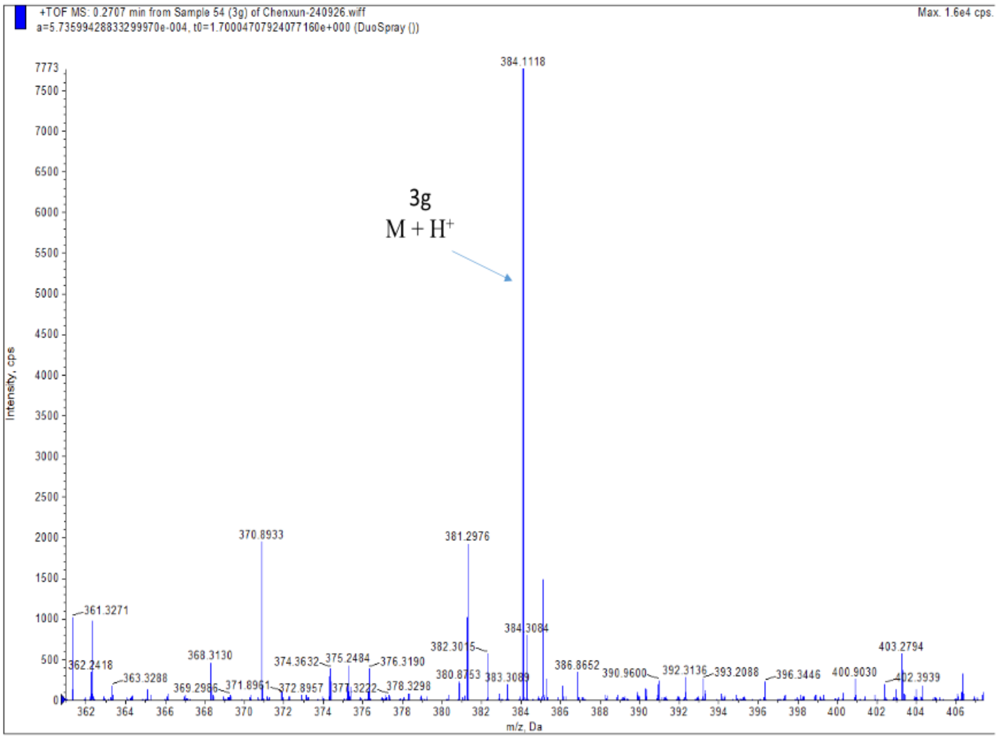
+TOF MS: 0.2707 min from Sample 54 (3g) of Chenxun-240926.wiff
a=5.73599428833299970e-004, t0=1.70004707924077160e+000 (DuoSpray ())
Max. 1.6e4 cps.
3g
M + H+
384.1118
370.8933
381.2976
361.3271
384.3084
403.2794
Intensity, cps
m/z, Da

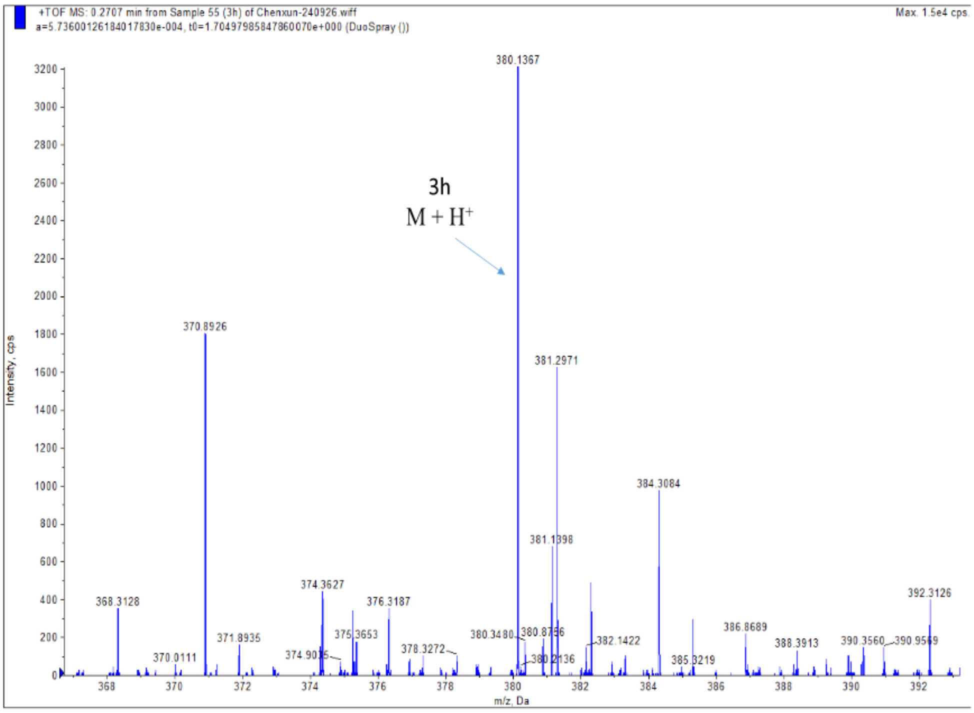
+TOF MS: 0.2707 min from Sample 55 (3h) of Chenxun-240926.wiff
a=5.73600126184017830e-004, t0=1.70497985847860070e+000 (DuoSpray ())
Max. 1.5e4 cps.
3h
M + H+
380.1367
370.8926
381.2971
384.3084
374.3627
392.3126
Intensity, cps
m/z, Da

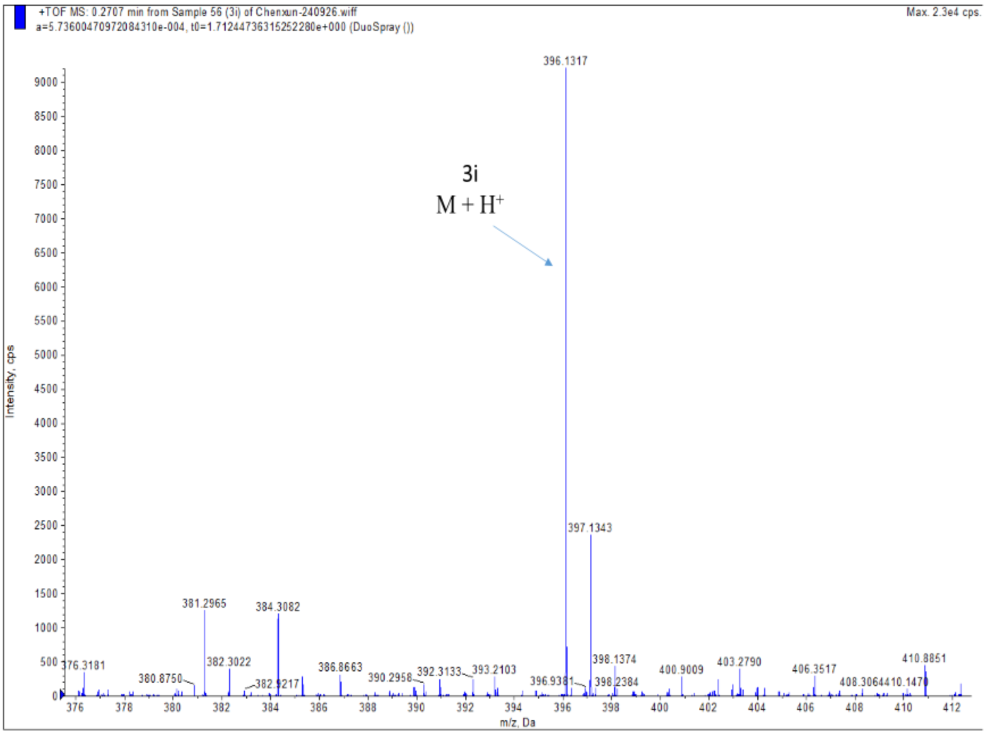
+TOF MS: 0.2707 min from Sample 56 (3i) of Chenxun-240926.wiff
a=5.73600470972084310e-004, t0=1.71244736315252280e+000 (DuoSpray ())
Max. 2.3e4 cps.
396.1317
3i
M + H+
397.1343
381.2965
384.3082
Intensity, cps
m/z, Da

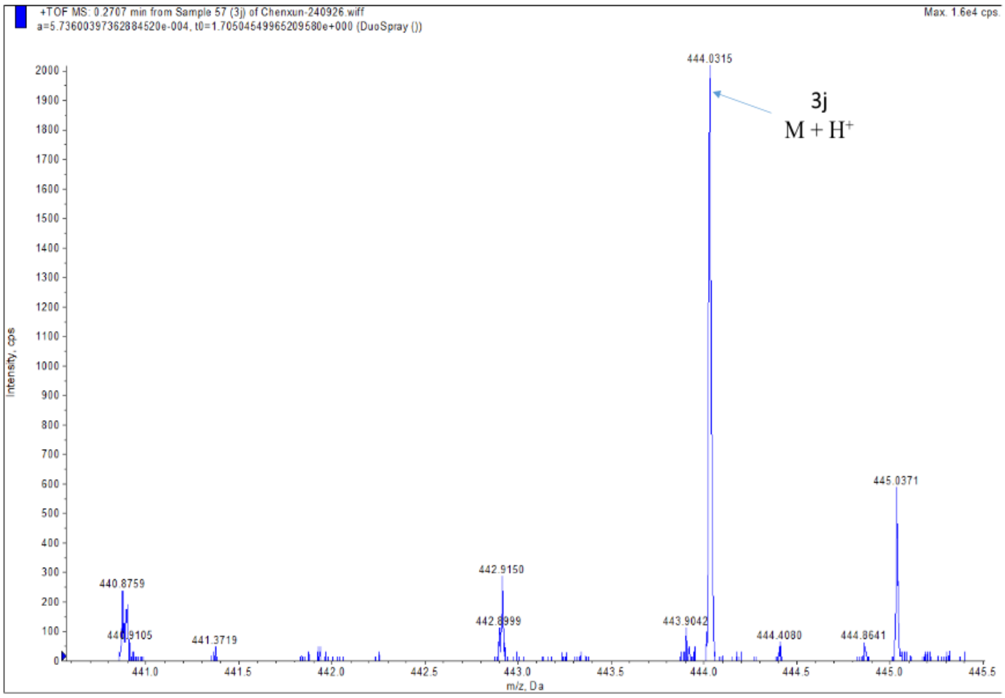
+TOF MS: 0.2707 min from Sample 57 (3j) of Chenxun-240926.wiff
a=5.73600397362884520e-004, t0=1.70504549965209580e+000 (DuoSpray ())
Max. 1.6e4 cps.
444.0315
3j
M + H+
445.0371
442.9150
440.8759
Intensity, cps
m/z, Da

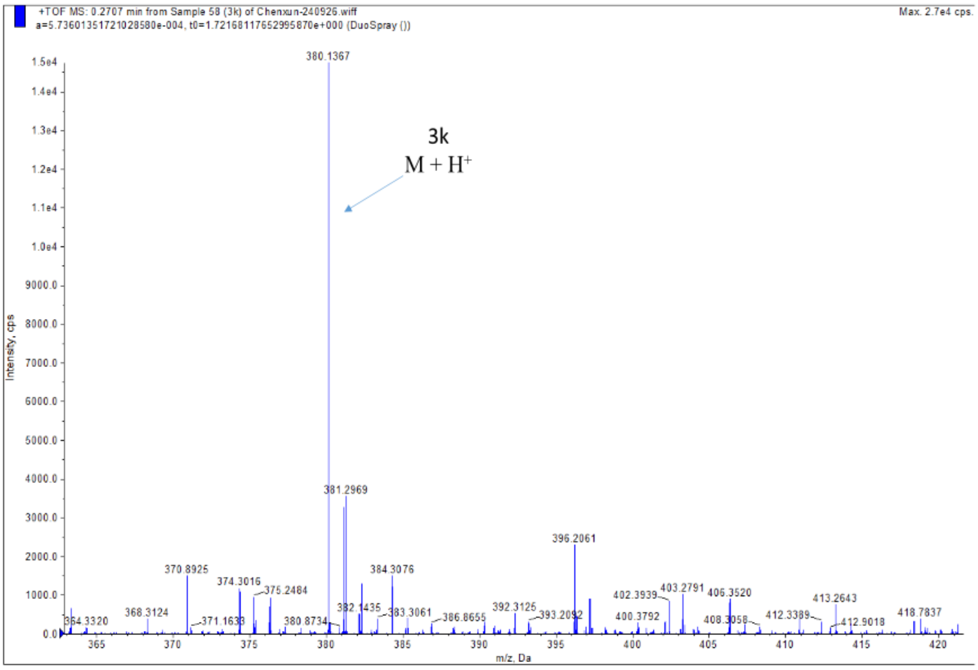

+TOF MS: 0.2707 min from Sample 58 (3k) of Chenxun-240926.wiff
Max. 2.7e4 cps.
3k
M + H+
380.1367
381.2969
396.2061
Intensity, cps
m/z, Da

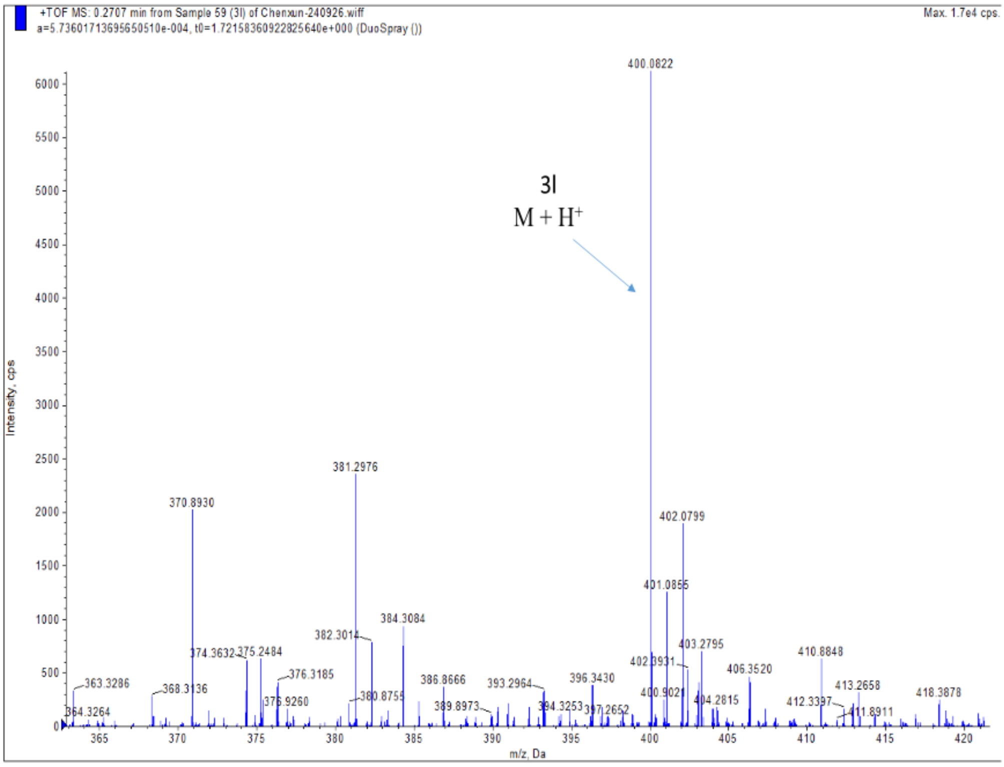

+TOF MS: 0.2707 min from Sample 59 (3l) of Chenxun-240926.wiff
Max. 1.7e4 cps.
3l
M + H+
400.0822
381.2976
370.8930
402.0799
Intensity, cps
m/z, Da

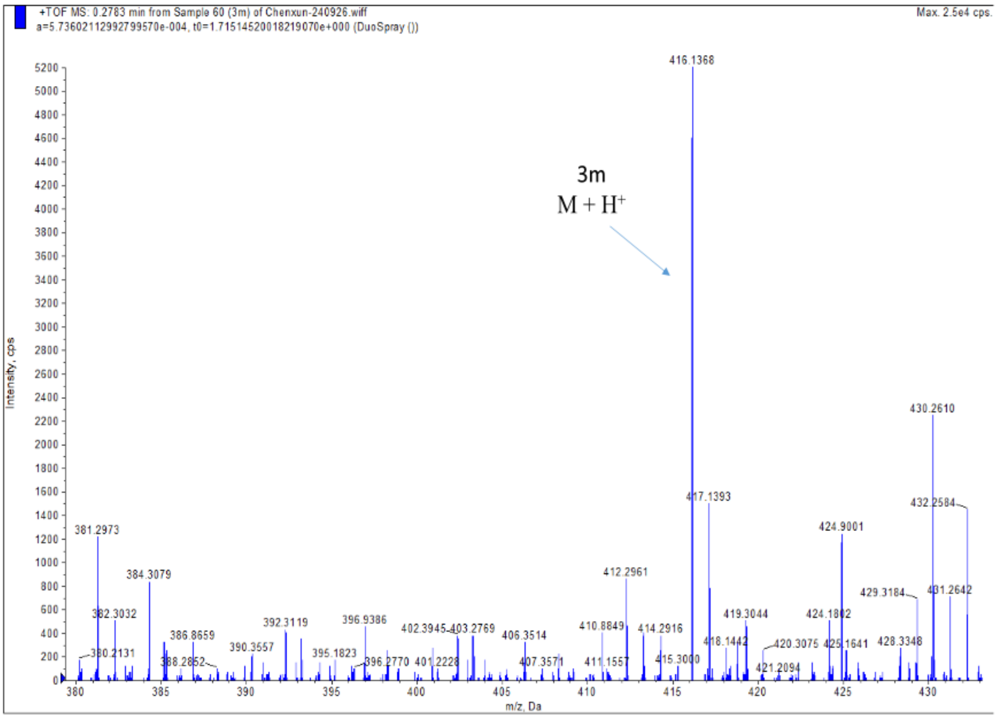
+TOF MS: 0.2783 min from Sample 60 (3m) of Chenxun-240926.wiff
Max. 2.5e4 cps.
3m
M + H+
416.1368
430.2610
381.2973
384.3079
424.9001
417.1393
432.2584
412.2961
Intensity, cps
m/z, Da

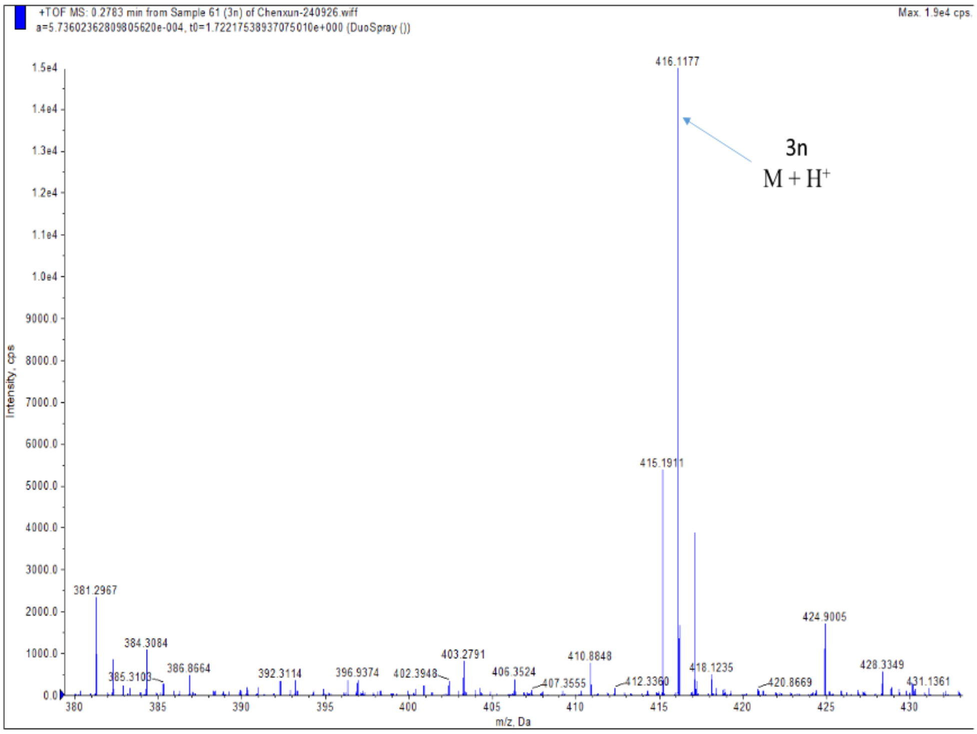
+TOF MS: 0.2783 min from Sample 61 (3n) of Chenxun-240926.wiff
Max. 1.9e4 cps.
3n
M + H+
416.1177
415.1911
381.2967
424.9005
384.3084
403.2791
Intensity, cps
m/z, Da

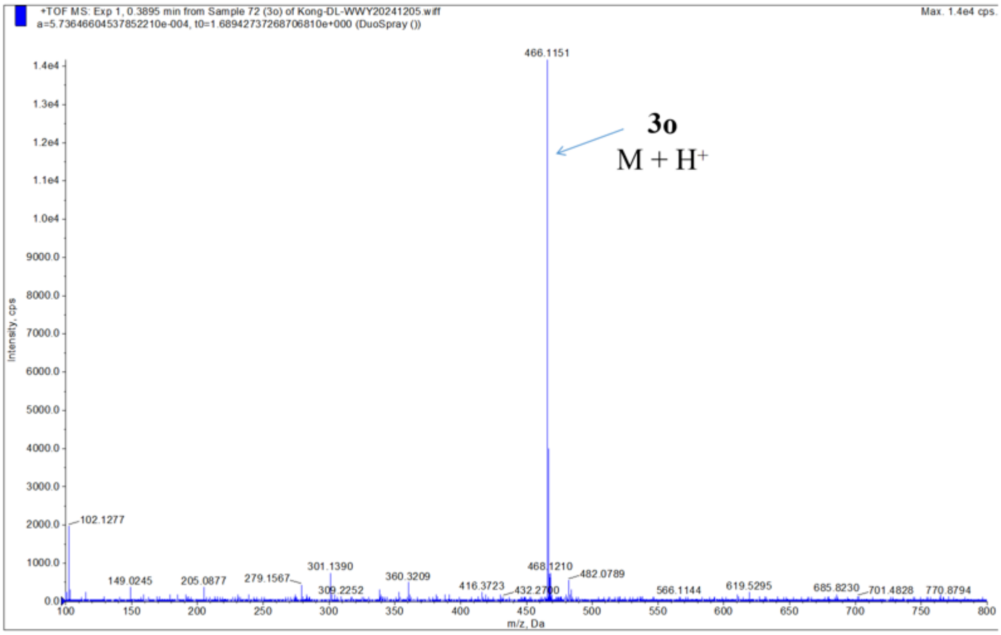
+TOF MS: Exp 1, 0.3895 min from Sample 72 (3o) of Kong-DL-WWY20241205.wiff
a=5.73646604537852210e-004, t0=1.68942737268706810e+000 (DuoSpray ())
Max. 1.4e4 cps.
466.1151
3o
M + H+
Intensity, cps
m/z, Da

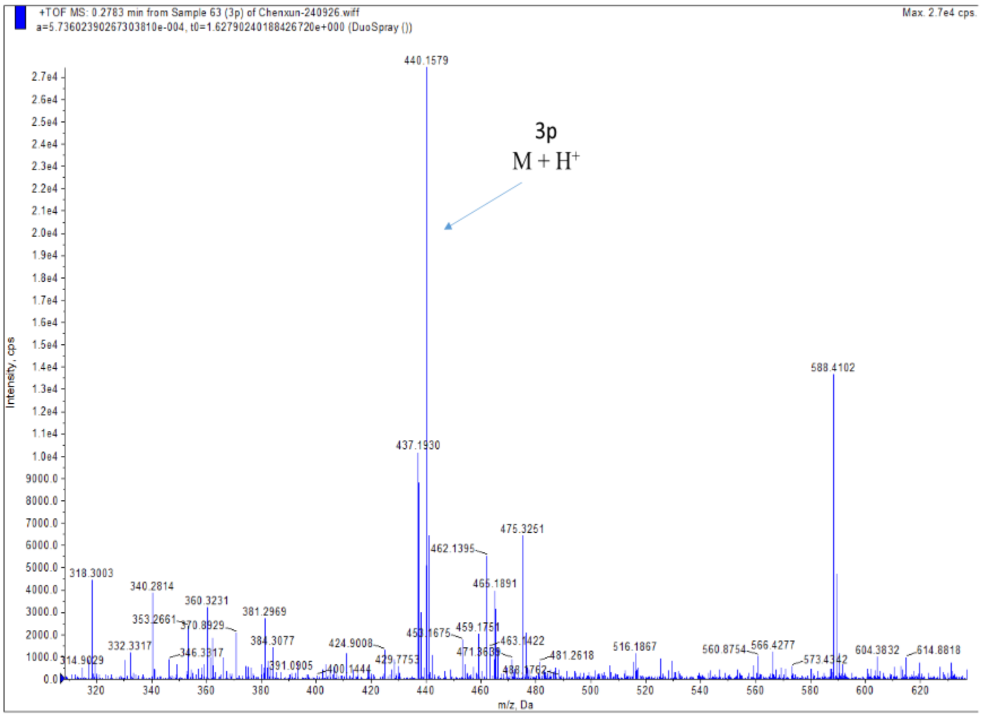
+TOF MS: 0.2783 min from Sample 63 (3p) of Chenxun-240926.wiff
a=5.73602390267303810e-004, t0=1.62790240188426720e+000 (DuoSpray ())
Max. 2.7e4 cps.
440.1579
3p
M + H+
588.4102
Intensity, cps
m/z, Da

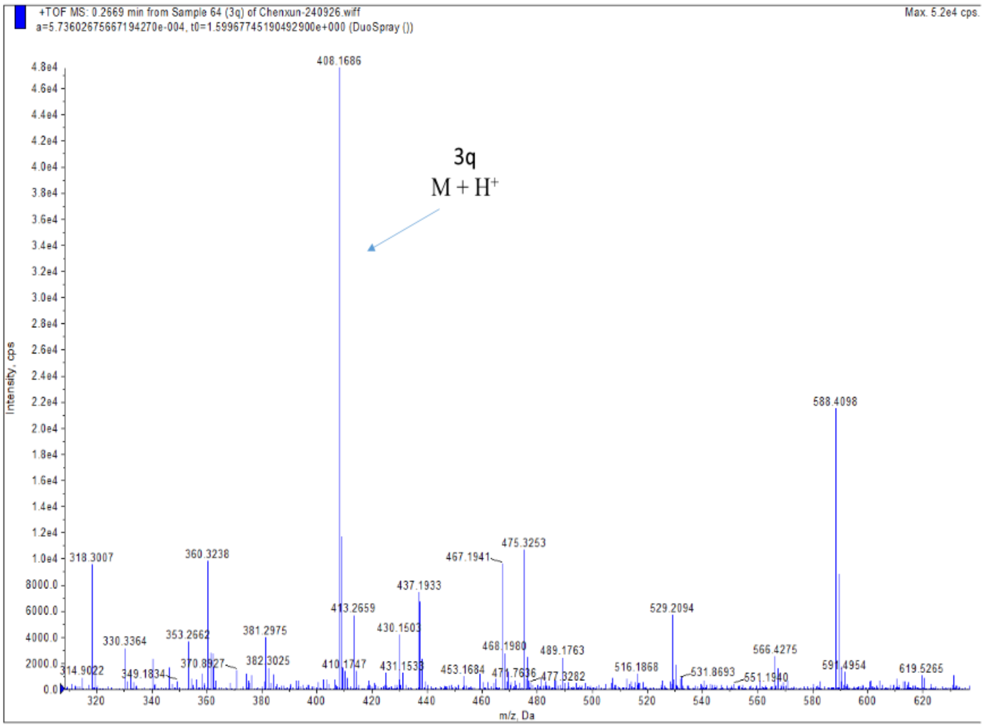
+TOF MS: 0.2669 min from Sample 64 (3q) of Chenxun-240926.wiff
Max. 5.2e4 cps.
408.1686
3q
M + H+
588.4098
475.3253
467.1941
360.3238
318.3007
437.1933
529.2094
Intensity, cps
m/z, Da

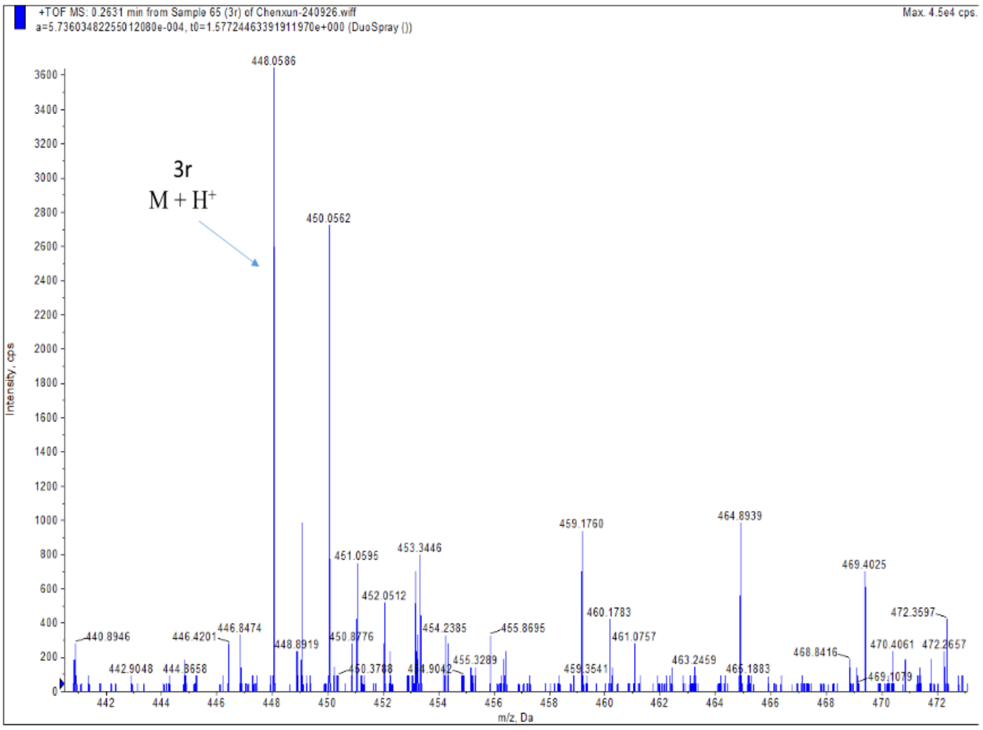
+TOF MS: 0.2631 min from Sample 65 (3r) of Chenxun-240926.wiff
Max. 4.5e4 cps.
448.0586
3r
M + H+
450.0562
459.1760
464.8939
469.4025
Intensity, cps
m/z, Da

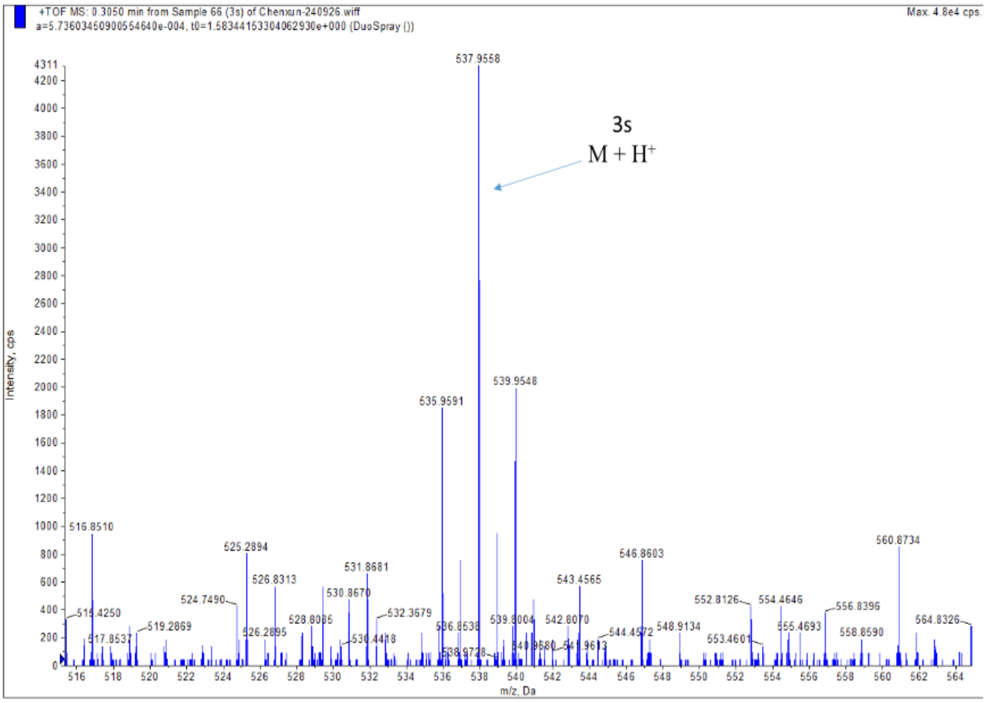
+TOF MS: 0.3050 min from Sample 66 (3s) of Chenxun-240926.wiff
a=5.73603450900554640e-004, t0=1.58344153304062930e+000 (DuoSpray ())
Max. 4.8e4 cps.
537.9558
3s
M + H+
539.9548
535.9591
516.8510
525.2894
560.8734
546.8603
531.8681
526.8313
543.4565
530.8670
524.7490
552.8126
554.4646
556.8396
532.3679
564.8326
Intensity, cps
m/z, Da

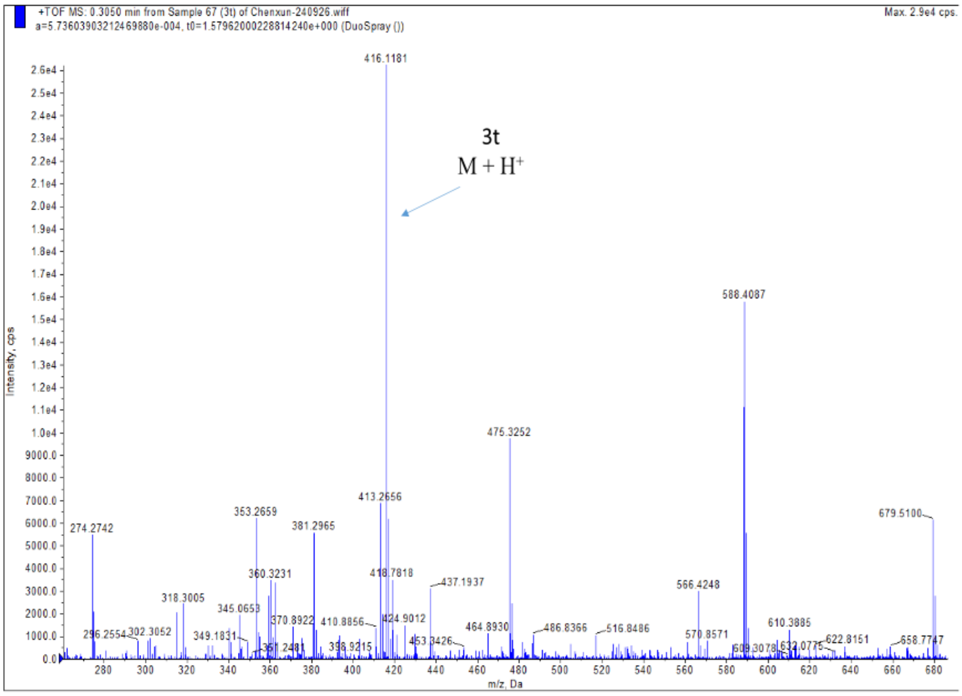
+TOF MS: 0.3050 min from Sample 67 (3t) of Chenxun-240926.wiff
a=5.73603903212469880e-004, t0=1.57962000228814240e+000 (DuoSpray ())
Max. 2.9e4 cps.
416.1181
3t
M + H+
588.4087
475.3252
413.2656
353.2659
679.5100
381.2965
274.2742
360.3231
418.7818
437.1937
566.4248
318.3005
345.0653
610.3885
Intensity, cps
m/z, Da

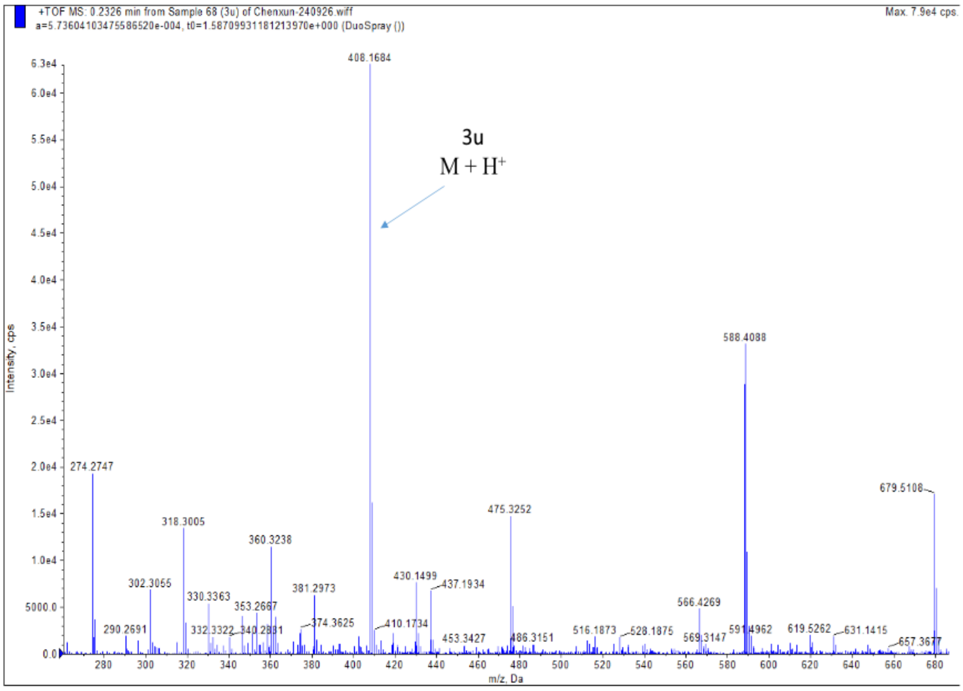
+TOF MS: 0.2326 min from Sample 68 (3u) of Chenxun-240926.wiff
a=5.73604103475586520e-004, t0=1.58709931181213970e+000 (DuoSpray ())
Max. 7.9e4 cps.
3u
M + H+
408.1684
588.4088
274.2747
679.5108
475.3252
318.3005
360.3238
Intensity, cps
m/z, Da

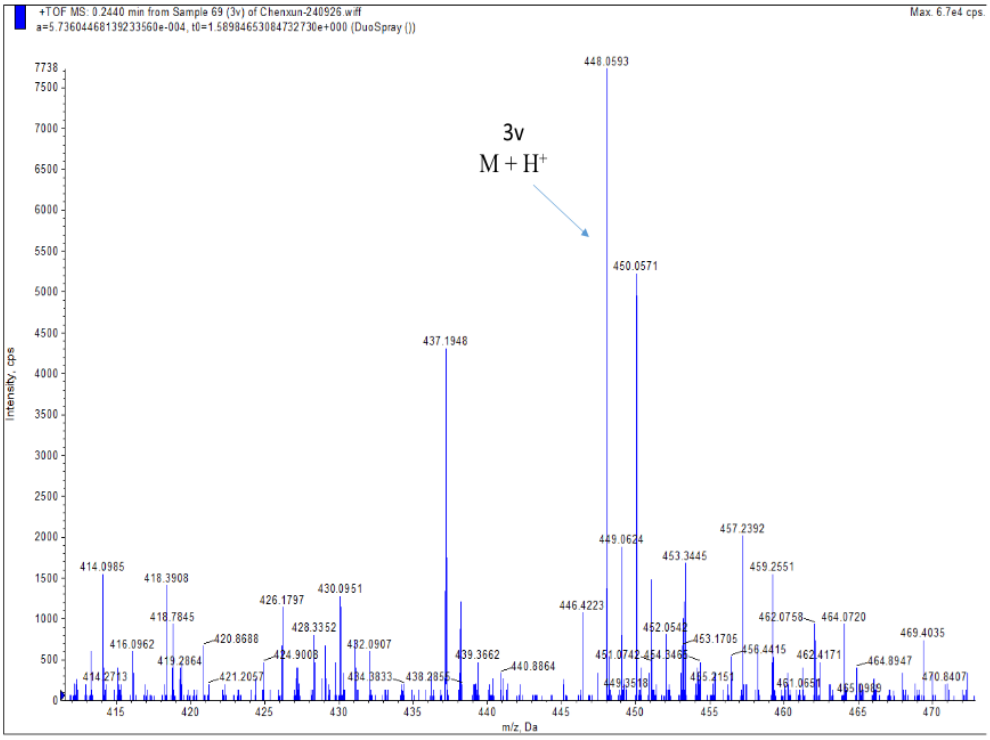
+TOF MS: 0.2440 min from Sample 69 (3v) of Chenxun-240926.wiff
a=5.73604468139233560e-004, t0=1.58984653084732730e+000 (DuoSpray ())
Max. 6.7e4 cps.
3v
M + H+
448.0593
450.0571
437.1948
457.2392
449.0624
453.3445
459.2551
414.0985
418.3908
430.0951
Intensity, cps
m/z, Da

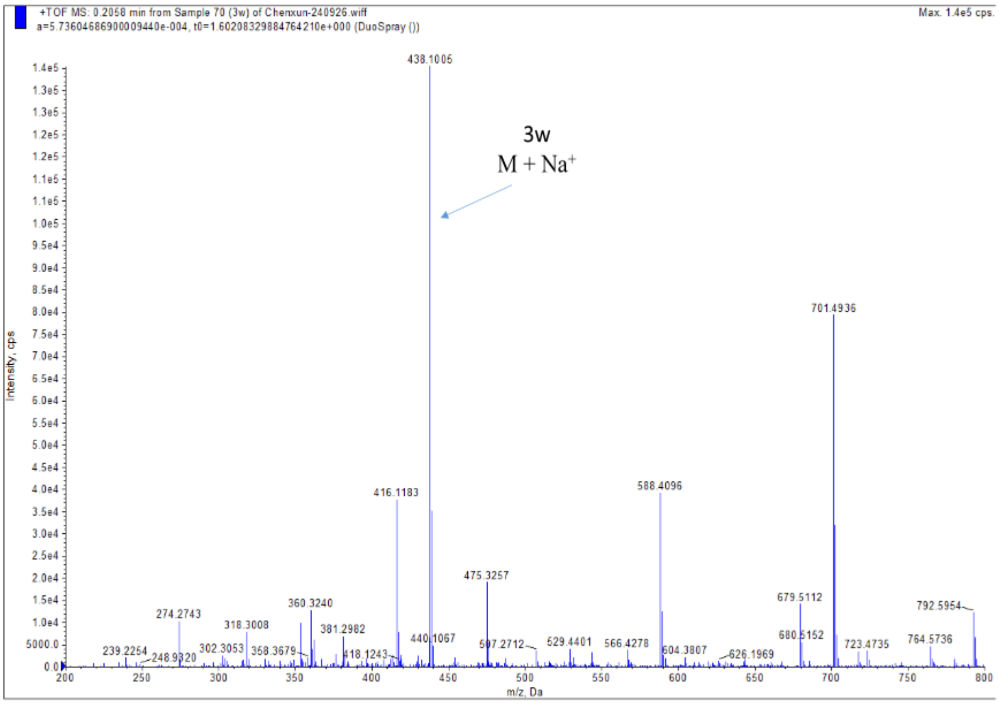
+TOF MS: 0.2058 min from Sample 70 (3w) of Chenxun-240926.wiff
Max. 1.4e5 cps.
3w
M + Na+
438.1006
701.4936
588.4096
416.1183
475.3257
Intensity, cps
m/z, Da

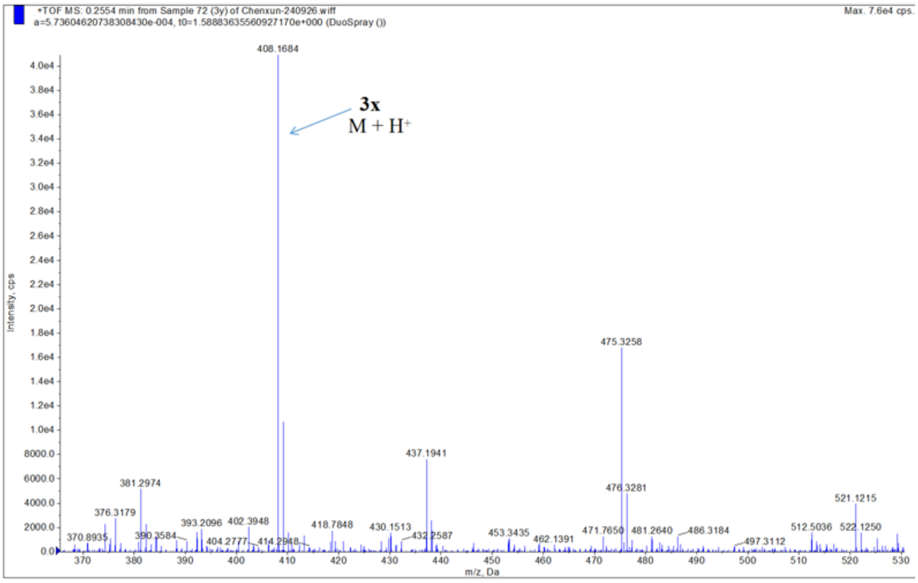
+TOF MS: 0.2554 min from Sample 72 (3y) of Chenxun-240926.wiff
Max. 7.6e4 cps.
3x
M + H+
408.1684
475.3258
437.1941
381.2974
Intensity, cps
m/z, Da

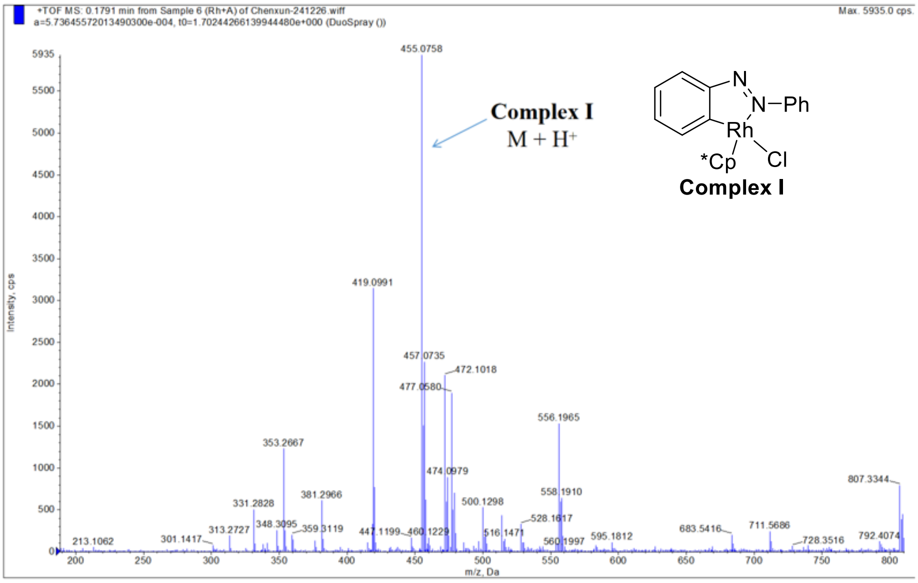
+TOF MS: 0.1791 min from Sample 6 (Rh+A) of Chenxun-241226.wiff
a=5.73645572013490300e-004, t0=1.70244266139944480e+000 (DuoSpray ())
Max. 5935.0 cps.
Intensity, cps
m/z, Da
455.0758
Complex I
M + H+
419.0991
457.0735
472.1018
477.0580
556.1965
353.2667
474.0979
558.1910
807.3344
500.1298
381.2966
331.2828
528.1617
348.3095
359.3119
447.1199
460.1229
516.1471
711.5686
683.5416
595.1812
560.1997
313.2727
301.1417
213.1062
728.3516
792.4074
N
N−Ph
Rh
*Cp
Cl
Complex I
